# Unveiling KuQuinone Redox Species: An Electrochemical and Computational Cross Study

**DOI:** 10.1021/acs.joc.1c00165

**Published:** 2021-04-07

**Authors:** Francesca Valentini, Federica Sabuzi, Valeria Conte, Victor N. Nemykin, Pierluca Galloni

**Affiliations:** † Department of Chemical Science and Technologies, University of Rome Tor Vergata, Via della Ricerca Scientifica, Rome 00133, Italy; ‡ Department of Chemistry, University of Tennessee, Knoxville, Tennessee 37996, United States; § Department of Chemistry, University of Manitoba, Winnipeg, Manitoba R3T 2N2, Canada

## Abstract

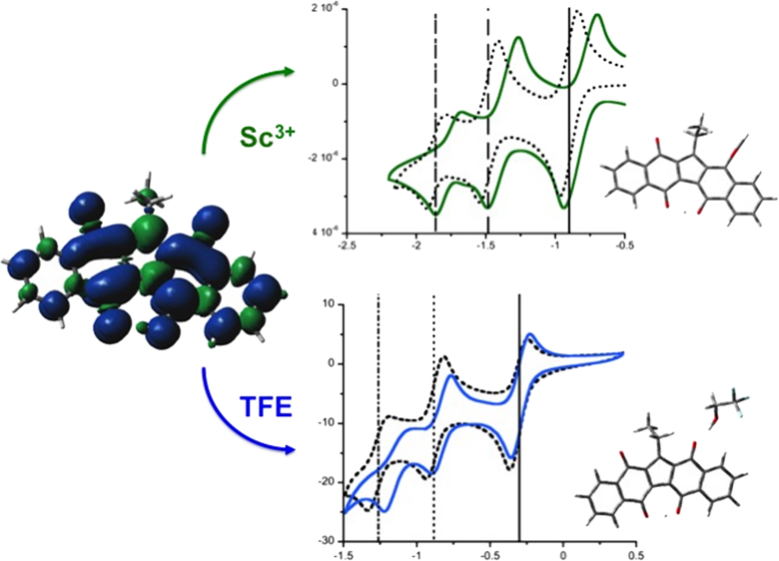

The study of the electrochemical properties of variegated quinones is a fascinating topic in chemistry. In fact, redox reactions occurring with quinoid scaffolds are essential for most of their applications in biological systems, in photoelectrochemical devices, and in many other fields. In this paper, a detailed investigation of KuQuinones’ redox behavior is presented. The distinctiveness of such molecules is the presence in the structure of two condensed naphthoquinone units, which implies the possibility to undergo multiple one-electron reduction processes. Solvent, supporting electrolyte, and hydrogen bond donor species effects have been elucidated. Changing the experimental parameters provoked significant shift of the redox potential for each reduction process. In particular, additions of 2,2,2-trifluoroethanol as a hydrogen bond donor in solution as well as Lewis acid coordination were crucial to obtain important shifts of the redox potentials toward more favorable values. UV–vis–NIR spectroelectrochemical experiments and DFT calculations are also presented to clarify the nature of the reduced species in solution.

## Introduction

Nowadays, the application of quinone-based molecules as efficient electron carriers in biological processes,^1^ in energy storage,^2^ and in CO_2_ reduction^3−5^ devices and their pharmacological activity in different diseases^6^ are extensively investigated. Quinones’ key role in the aforementioned applications is mainly due to their facile and highly tailorable redox chemistry.

Over the years, several electrochemical studies have been carried out to better understand the reactivity and the stability of the electro-generated species. In particular, in aqueous buffer, quinones are characterized by a reversible one-step two-electron reduction process that leads to hydroquinone species. The half-wave potential of such a process linearly changes with pH, according to the Nernst equation.^7^ Under acidic conditions, the reduction is a two-proton two-electron process, while a two-electron process occurs at alkaline pH levels. Conversely, in aprotic organic media, quinones undergo two subsequent one-electron reductions to give the semiquinone (Q^·^^–^) and the hydroquinone (Q^2–^) species.^8^ The first process is always reversible, while the second one is at least quasi-reversible, depending on the scan speed.

Several factors, namely, solvent polarity, nature of the supporting electrolyte, intra- or intermolecular hydrogen bonding, and the presence of acidic additives in solution, may stabilize quinone reduced species, thus causing a remarkable half-wave potential shift. In particular, over the past decades, much attention has been paid to the deep comprehension of quinones’ electrochemistry in the presence of weak or strong hydrogen bond donors (HBDs). Also, it has been recognized that hydrogen bonds may control the structure and the functionality of some biologically active quinones.^1,9^ In fact, HBDs can positively shift the half-wave potential of quinones, and this shift cannot be attributed to the proton couple electron transfer mechanism.^10^ Accordingly, different models have been developed to estimate the stoichiometry of the hydrogen-bonded complexes and to calculate their thermodynamic constant (*K*_eq_).^10,11^ In such representation, *n* and *m* are commonly used to indicate the number of HBD molecules interacting with the electroactive quinoid species, i.e., Q^·^^–^[HBD]*_n_* and Q^2–^[HBD]*_m_*. In particular, the model of single global equilibrium^10^ and the model of equilibria for *n* and *m* successive stages^11^ have been developed. Both models adopt the approach of the ion–ion association, and they can be used only if the redox species have a great interaction with the HBD. The first model considers the association between the quinone and HBD occurring in a single step, while the second one assumes that the association takes place by successive equilibria.

Due to their well-known electrochemical properties, quinones have been also taken as references for estimating the strength of various hydrogen bonding donor species in organic solvents^12^ and to quantify the moisture content.^7,13^

**Figure 1:**
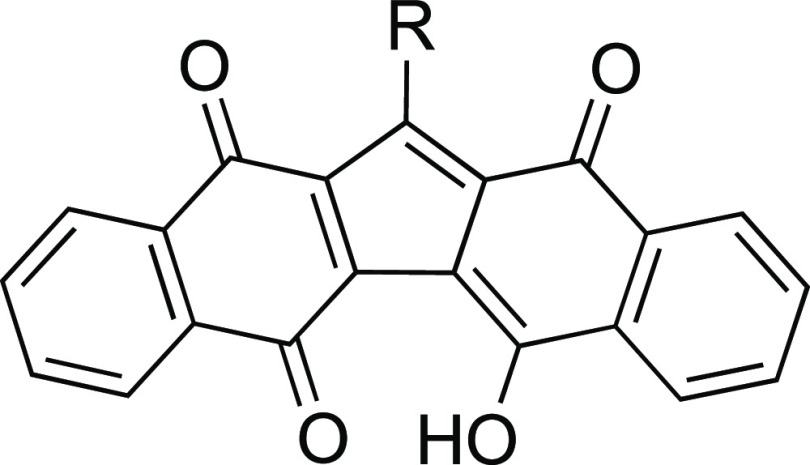
General structure of KuQuinones.

Our interest in quinone chemistry started in 2012, when we first reported the synthesis of a new family of quinoid compounds called KuQuinones (KuQs)^14−19^ (Figure 1).

To the best of our knowledge, KuQ synthesis was the first published one-pot reaction to obtain a polycyclic quinone.^20^ The planar fully conjugated skeleton of KuQs confers them peculiar spectroscopic^18^ and electrochemical properties. In fact, two distinctive absorption bands are usually detected in the visible region of the spectrum of KuQs, while the electrochemical profile is characterized by three processes, and the first reduction potential, to give KuQ^·^^–^, is very close to 0 V. The combination of such intriguing properties made KuQuinones suitable molecules as sensitizers in different photoelectrochemical cells.^16,17^ More recently, an appropriate KuQuinone derivative coupled with a ruthenium polyoxometalate catalyst has been anchored on nanostructured tin oxide and it has been used as a harvesting material for a new photoanode in water oxidation. Such application demonstrated KuQ’s capability to manage proton-coupled electron transfer, thus leading to very good results in terms of faradaic efficiency of the cell.^19^

KuQs’ application in the photoelectrochemical field prompted us to investigate the features of such compounds in order to further understand the nature of redox species involved in their electron transfer processes as well as to increase their efficiency as photosensitizers. Therefore, in this paper, the electrochemical and spectroelectrochemical behavior of a model KuQ derivative, such as 1-ethylKuQuinone (KuQEt), is reported as a function of the solvent polarity, the nature of the supporting electrolyte, and the presence of different additives in the system.

## Results and Discussion

### Electrochemical Study

**Figure 2:**
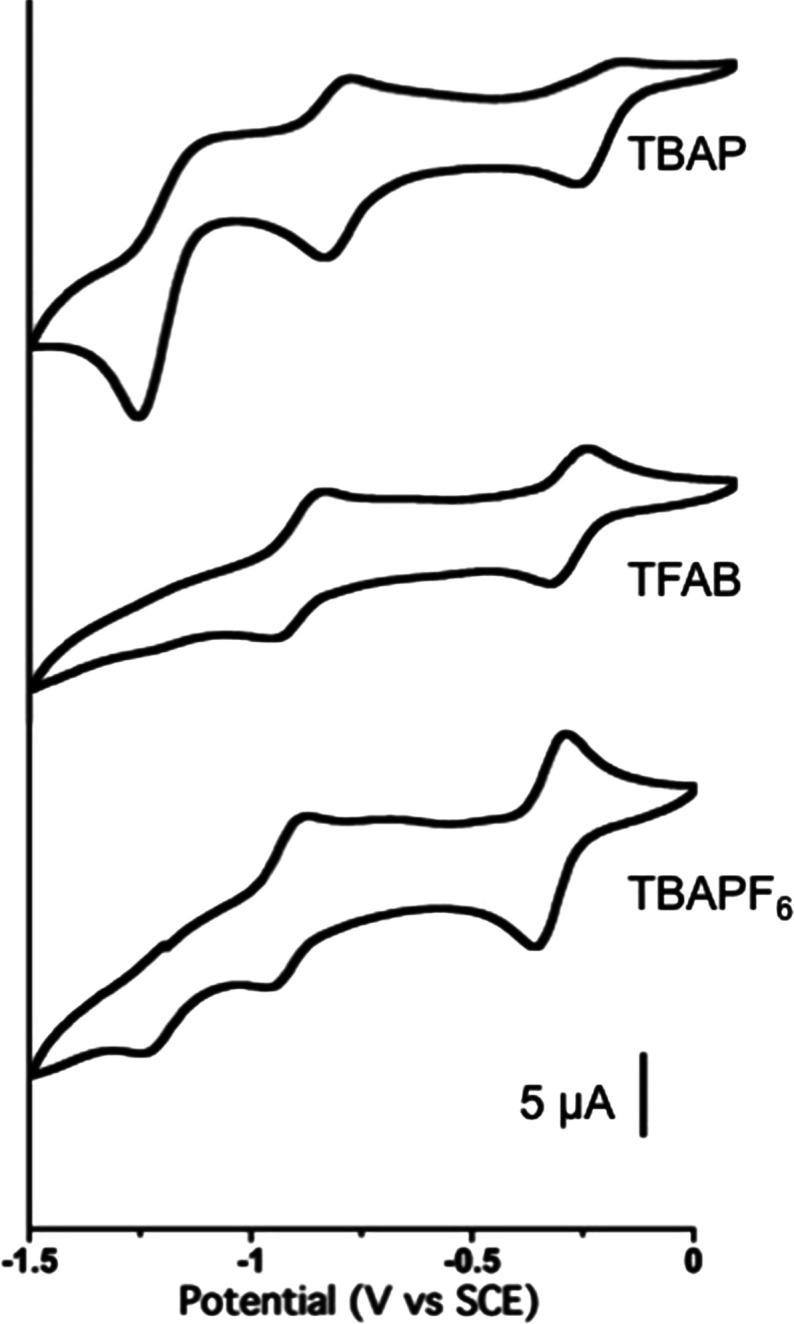
Cyclic voltammetry of KuQEt in CH_2_Cl_2_ and in different supporting electrolytes (0.1 M): TBAP (top), TFAB (center), and TBAPF_6_ (bottom). Platinum disk as a working electrode and SCE as a reference.

**Scheme 1:**
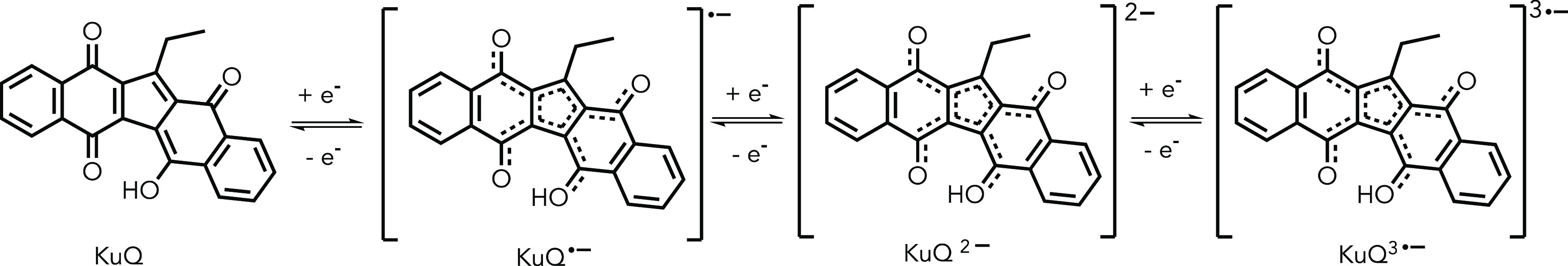
KuQEt Reduction Steps

The electrochemical profile of KuQEt in aprotic organic solvents, like CH_2_Cl_2_, is presented in Figure 2. The voltammogram is characterized by three quasi-reversible cathodic waves (Δ*E*_p_ > 59 mV), corresponding to the formation at the electrode surface of the radical anion, the dianion, and the radical trianion species, according to the reaction scheme depicted in Scheme 1.

The half-wave potentials for the three processes are, respectively, *E*^(1)^_1/2_ = −0.3 V, *E*^(2)^_1/2_ = −0.8 V, and *E*^(3)^_1/2_ = −1.2 V vs SCE in CH_2_Cl_2_ with TBAP (0.1 M) as the supporting electrolyte, a platinum disk as the working electrode, and SCE as the reference.

Interestingly, the first reduction peak is positively shifted with respect to other biologically important molecules, such as vitamin K_1_^7,13,14^ and Bis-coenzyme Q_0_,^14^ thus making KuQuinones excellent electron acceptor molecules.

As a matter of fact, the intramolecular hydrogen bond occurring between the enol proton and the vicinal carbonyl oxygen in KuQs is responsible for the shift of the reduction potential with respect to other simpler quinones.^21^ Notably, the X-ray structure of KuQuinones^14^ confirmed the presence of such an intramolecular H-bond, which is also maintained in slightly polar solvents, such as dichloromethane.^18^ Due to the absence of oxidizable groups, no anodic waves have been detected.

Voltammograms in Figure 2 indicate that, in the presence of weakly coordinating supporting electrolytes, significant changes in the third reduction process occur. In fact, with tetrabutylammonium hexafluorophosphate (TBAPF_6_), the three characteristic reductions can be detected, but the third one becomes irreversible, while in the presence of the even less coordinating tetrabutylammonium tetrakis(pentafluorophenyl)borate (TFAB), only two processes are observed. It can be assumed that in CH_2_Cl_2_/TFAB, an intimate ion pair between KuQ^2–^ and tetrabutylammonium ions forms, leading to stable species, which do not undergo further reduction. Similarly, in CH_2_Cl_2_/TBAPF_6_, ion pairing between KuQ^3^^·^^–^ and supporting electrolyte cations occurs, thus inhibiting reversibility.^22,23^

Therefore, tetrabutylammonium perchlorate (TBAP) has been chosen as the supporting electrolyte for further experiments. Previous works demonstrated that solvent polarity affects the acid–base equilibrium in KuQs.^18^ In fact, because of the peculiar acidity of the enol proton (measured p*K*a = 4.7^18^), equilibrium between the enol and the enolate forms of KuQs can occur in solution, leading to significant variations in the UV–vis spectra. Accordingly, acid–base equilibrium also affects KuQuinones’ redox properties. Hence, in this study, CH_2_Cl_2_ and DMF were used as solvents.

**Figure 3:**
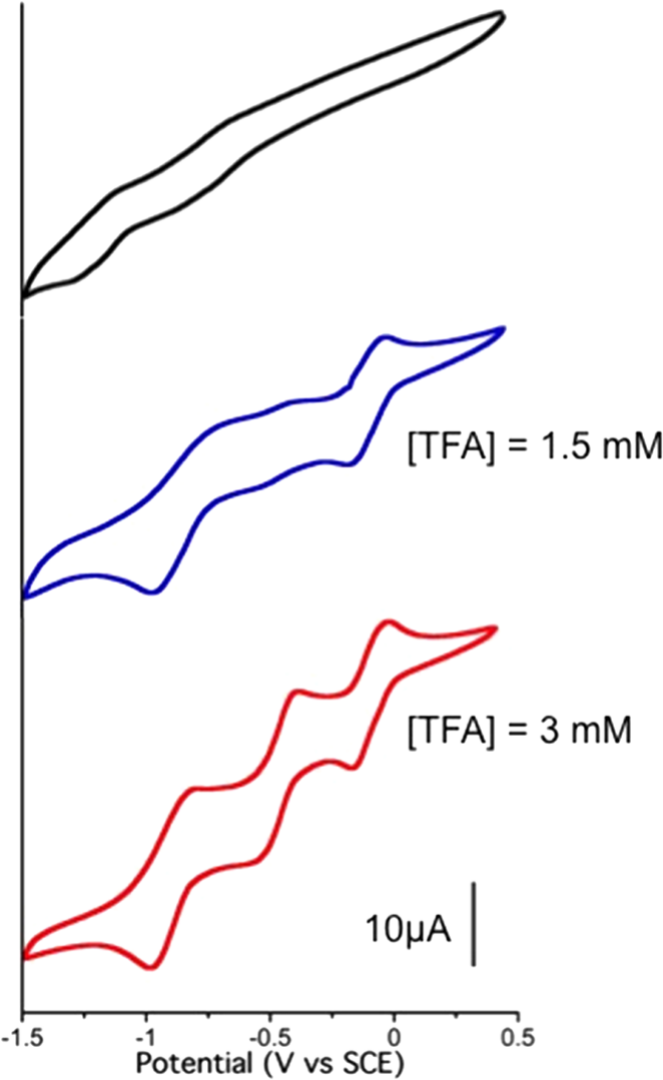
Cyclic voltammetry of KuQEt (1 mM) in 1 day aged DMF solution with stepwise addition of TFA. Glassy carbon as a working electrode and SCE as a reference electrode; supporting electrolyte: TBAP (0.1 M).

As indicated by the UV–vis spectra in CH_2_Cl_2_ (Figure S2), KuQ enol, i.e., the protonated form, is the only species in solution. On the contrary, in aprotic polar solvents, such as DMF, KuQuinone may be found in a mixture of enol/enolate or in its completely deprotonated form.^18^ Specifically, in freshly prepared solutions of KuQ in anhydrous DMF, the enol/enolate mixture is detected, as proved by the three intense bands at 370, 520, and 560 nm in the UV–vis spectrum. Conversely, in 1 day aged DMF solution, deprotonation occurs, likely due to the presence of moisture traces.^18^ In fact, the enolate form is the only one observed (as indicated by the blue shift of the spectrum). To note, the enol species alone is not observed in DMF. Thus, the electrochemical profile of a 1 day aged solution of KuQEt in DMF (which exclusively contains the deprotonated KuQEt) is presented in Figure 3 (black trace).

In such a solvent, two very broad reduction processes are observed, and the first peak is negatively shifted about 500 mV with respect to the same process in CH_2_Cl_2_. Such evidence supports the fact that the enolate species is present in solution since the reduction of a negatively charged molecule reasonably occurs at a more negative potential with respect to the neutral one. Moreover, the electron transfer rate is much slower with respect to that of the enol form in CH_2_Cl_2_.

Afterward, trifluoroacetic acid (TFA) has been added stepwise in a solution of KuQEt (1 mM) in DMF to protonate the enolate. By adding 1.5 equiv of TFA, both KuQ enol and enolate are present in solution, as shown in the UV–vis spectrum (Figure S3). Consequently, the obtained voltammogram is not well defined yet. Increasing the TFA amount up to 3 equiv leads to a well-resolved electrochemical profile, showing three quasi-reversible reduction processes, as for CH_2_Cl_2_. At higher TFA concentrations, H^+^ reduction is the main observed process (Figure S4). Importantly, the half-wave potentials of the three reduction processes in DMF in the presence of 3 equiv of TFA are significantly lower than those in CH_2_Cl_2_, indicating that KuQEt reduction is preferred in aprotic polar solvents. Such an effect may be also due to the presence of TFA excess in solution, which could establish hydrogen bond interactions with KuQEt carbonyl groups, likely favoring reduction processes. All electrochemical data are listed in Table 1. Notably, such experiments have been performed using glassy carbon (GC) as a working electrode. In fact, with GC, the obtained voltammogram shows three well-defined reduction processes and, in particular, the first one is reversible; therefore, better electrochemical behavior is observed with respect to the platinum working electrode (Figure S5).

**Table 1:** Redox Potentials of KuQEt (1 mM) in Different Solvents with TBAP (0.1 M) vs SCE^a^

solvents	*E*^(1)^_1/2_ (V)	Δ*E*^(1)^_p_ (mV)	*E*^(2)^_1/2_ (V)	Δ*E*^(2)^_p_ (mV)	*E*^(3)^_1/2_ (V)	Δ*E*^(3)^_p_ (mV)
KuQEt (CH_2_Cl_2_)	–0.30	120	–0.87	120	–1.26	140
KuQEt (DMF + 3 equiv TFA)	–0.095	130	–0.47	140	–0.90	190
KuQEt (DMF) 1 day aged solution	–0.76	160	–1.18	90		

a Glassy carbon as a working electrode and SCE as a reference.

**Figure 4:**
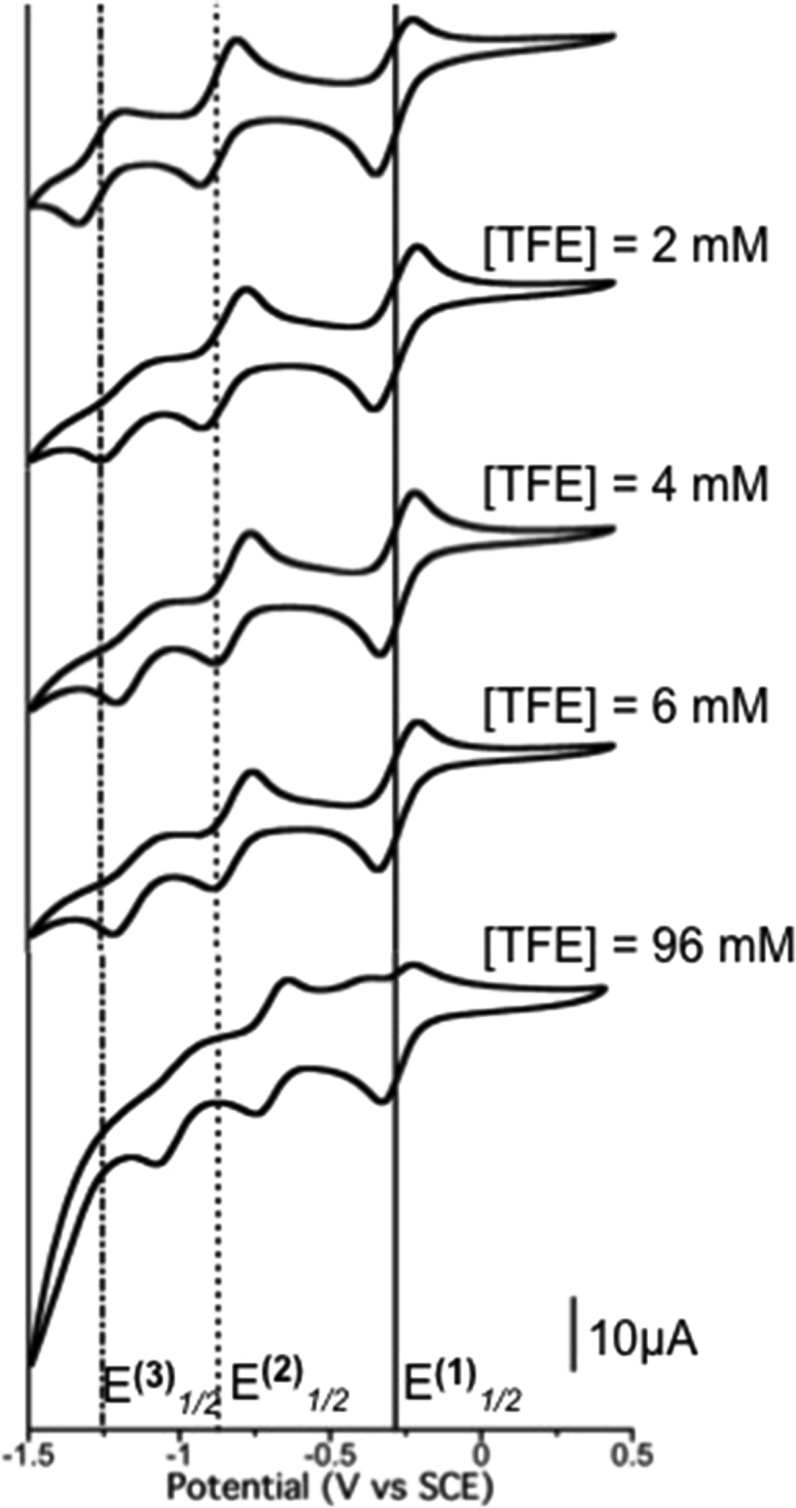
Cyclic voltammetry of KuQEt (2 mM) in CH_2_Cl_2_/TBAP (0.1 M), with increasing TFE concentration. Glassy carbon as a working electrode and SCE as a reference.

To better elucidate the hydrogen bond effect on the electrochemical profile of KuQuinones, cyclic voltammetry experiments with stepwise addition of 2,2,2-trifluorothanol (TFE), a non-acidic hydrogen bond donor additive,^24^ have been carried out (Figure 4).

Increasing the TFE amount from 1 to 3 equiv in CH_2_Cl_2_ leads to a shift of the second and third reduction processes toward more positive potentials. It is important to point out that at the same TFE concentration, the *E*^(3)^_1/2_ potential shift is higher than that of *E*^(2)^_1/2_ while, in order to achieve an appreciable *E*^(1)^_1/2_ shift, it is necessary to further increase the TFE amount up to 48 equiv. This effect is probably due to an increase in the electron density on the carbonyl oxygens after each reduction, which may lead to stronger hydrogen bond interactions with TFE. Consequently, a greater stabilization of the complex KuQ^3^^·^^–^[TFE]*_p_* with respect to the more oxidized species is expected. Similarly, the KuQ^2–^[TFE]*_m_* complex is reasonably more stable than the KuQ^·^^–^[TFE]*_n_* adduct.^12^ Nevertheless, the third quasi-reversible process becomes even more irreversible, increasing the TFE concentration. This fact may be explained from the high hydrogen bond stabilization in KuQ^3^^·^^–^[TFE]*_p_* species, which points to a more difficult re-oxidation process. Also, at [TFE] = 96 mM, the split of the first reduction process in two peaks can be clearly observed during the anodic scan. This is likely due to the co-existence in equilibrium of different hydrogen-bonded complexes, which are formed because of the large excess of TFE in solution.

Hydrogen bonding effects on electrochemical potentials of KuQuinone can be qualitatively described by the Δ*E*_obs_, which is the difference of the half-wave potential between two peaks in the resulting voltammogram.^12^ Accordingly, in this electrochemical system, *E*^(1)^_1/2_, i.e., the first reduction of KuQ to KuQ^·^^–^, has been taken as the reference because it is almost unaffected at low TFE concentrations. Unexpectedly, the Δ*E*_obs_ values of the second and third reduction processes at different TFE concentrations follow the same trend (Figure S6), strongly suggesting that in KuQ^3^^·^^–^[TFE]*_p_* and KuQ^2–^[TFE]*_m_*, similar hydrogen-bonding interactions have been established.

Quantitative analysis gives a deep comprehension of KuQuinone/TFE interactions in order to estimate the stoichiometry of the hydrogen-bonded complexes and the global equilibrium constant. Thus, the model for a single global equilibrium has been employed (eqs 1 and 2):^10^
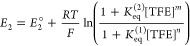
1
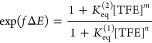
2

In such a model, the association between the electroactive species, i.e., KuQuinone, and the hydrogen bond donor, i.e., TFE, is assumed to occur in a single step. To note, no interaction between KuQ and TFE is expected since negligible changes in the absorption spectra were detected upon stepwise addition of TFE (Figure S7). Hence, the complexes under investigation are KuQ^·^^–^[TFE]*_n_*, KuQ^2–^[TFE]*_m_*, and KuQ^3^^·^^–^[TFE]*_p_*. Such a model is not successfully applied for the first and third reduction processes. In fact, as discussed above, *E*^(1)^_1/2_ was only slightly affected by the stepwise addition of TFE. Therefore, Δ*E* is too small to give meaningful quantitative results.^10^ Likewise, for the third reductive process, it is possible to model the hydrogen bond interactions for the KuQ^3^^·^^–^[TFE]*_p_* complex. However, the *E*^(3)^_1/2_ vs log[TFE] plot leads to a linear regression with *R*^2^ < 0.98, so the single global equilibrium model cannot be properly applied.^10,11^ Such a low *R*^2^ value is related to the gradual loss of reversibility of the third reduction process during the TFE additions. Consequently, quantitative analysis has been carried out for the KuQ^2–^[TFE]*_m_* complex. From eq 1,^10^ the slope of the plot *E*^(2)^_1/2_ vs log[TFE] gave a fractional *m* value of 1.5. Obviously, this value represents the average coordination number. Thus, one or two TFE molecules are likely hydrogen bonded to the KuQuinone dianion. Such a model allows also to estimate the binding constant *K*^(2)^_eq_, according to eq 2. Considering that *n* and *K*^(1)^_eq_ are negligible, a binding constant value of about 4 × 10^5^ M^–*m*^ was obtained, with *m* = 1.5. Such an estimated value is higher than the ones of other reported quinones, with the same *m* value, such as 2,5-dimethyl-1,4-benzoquinone (*m* = 1.4; *K*^(1)^_eq_ = 30) and 2,5-dimethoxy-1,4-benzoquinone (*m* = 2.0; K^(1)^_eq_ = 370).^10^ Probably, this is due to the extended conjugation of the KuQuinone skeleton that implies strong and stable interactions with one or two TFE molecules.

To clarify KuQs/TFE interactions, DFT calculations have been performed. The KuQuinone structure has been optimized in the vacuum and in dichloromethane using the polarizable continuum model for including the solvent effect, with the B3LYP functional and 6-31G+(d,p) basis set. Also, KuQuinone interacting with one and two TFE molecules has been modeled.

**Figure 5:**
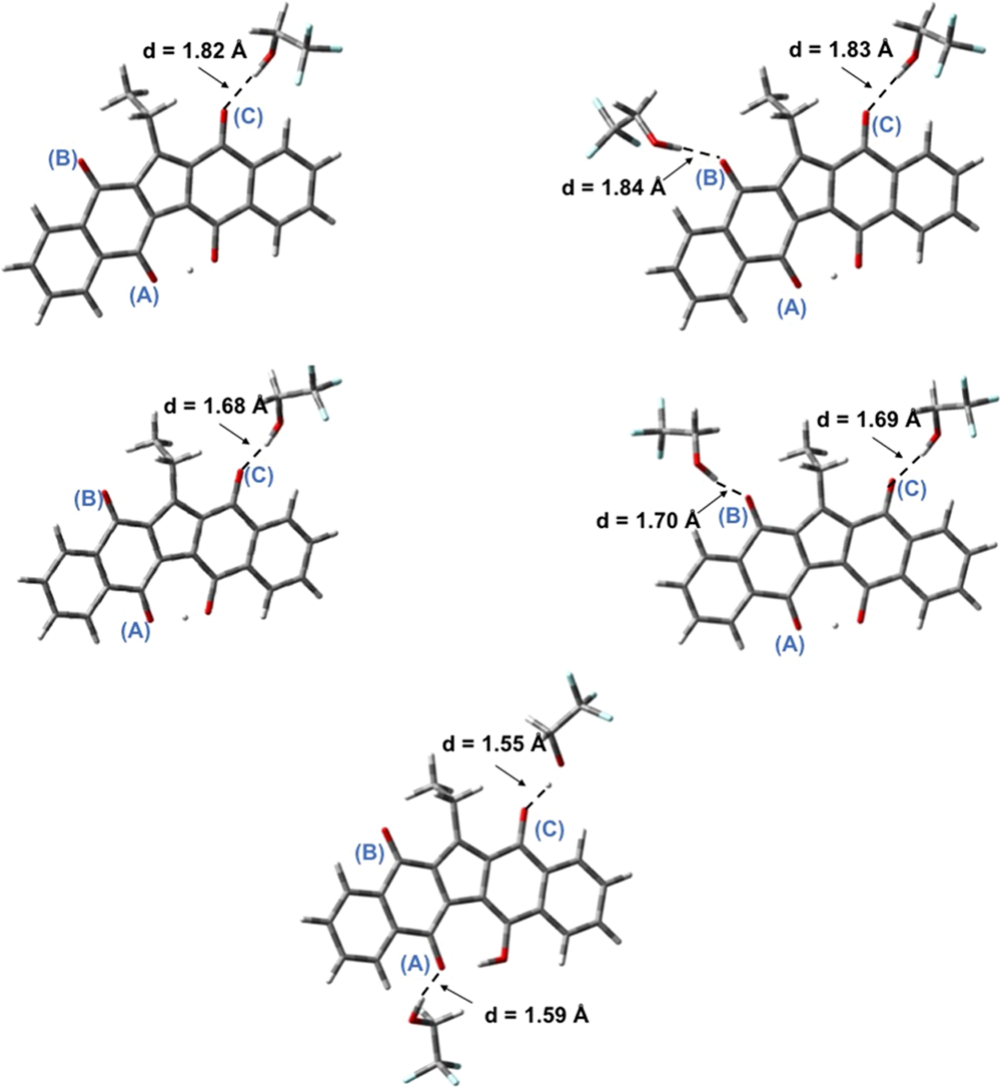
Optimized geometries in the vacuum of KuQ[TFE] and KuQ[TFE]_2_ species (top), KuQ^·^^–^[TFE] and KuQ^·^^–^[TFE]_2_ species (center), and KuQ^2–^[TFE]_2_ (bottom), with the B3LYP functional and 6-31G+(d,p) basis set. Color legend: gray, C; white, H; red, O; light blue, F.

In the case of the hydrogen-bonded complexes, correction for basis set superposition error (BSSE) has been applied using the counterpoise. Initially, the KuQ[TFE] complex has been modeled in three different geometries, with TFE interacting with the three carbonyl groups (defined as A, B, and C; Figure 5) in order to find the most stable structure.

**Scheme 2:**
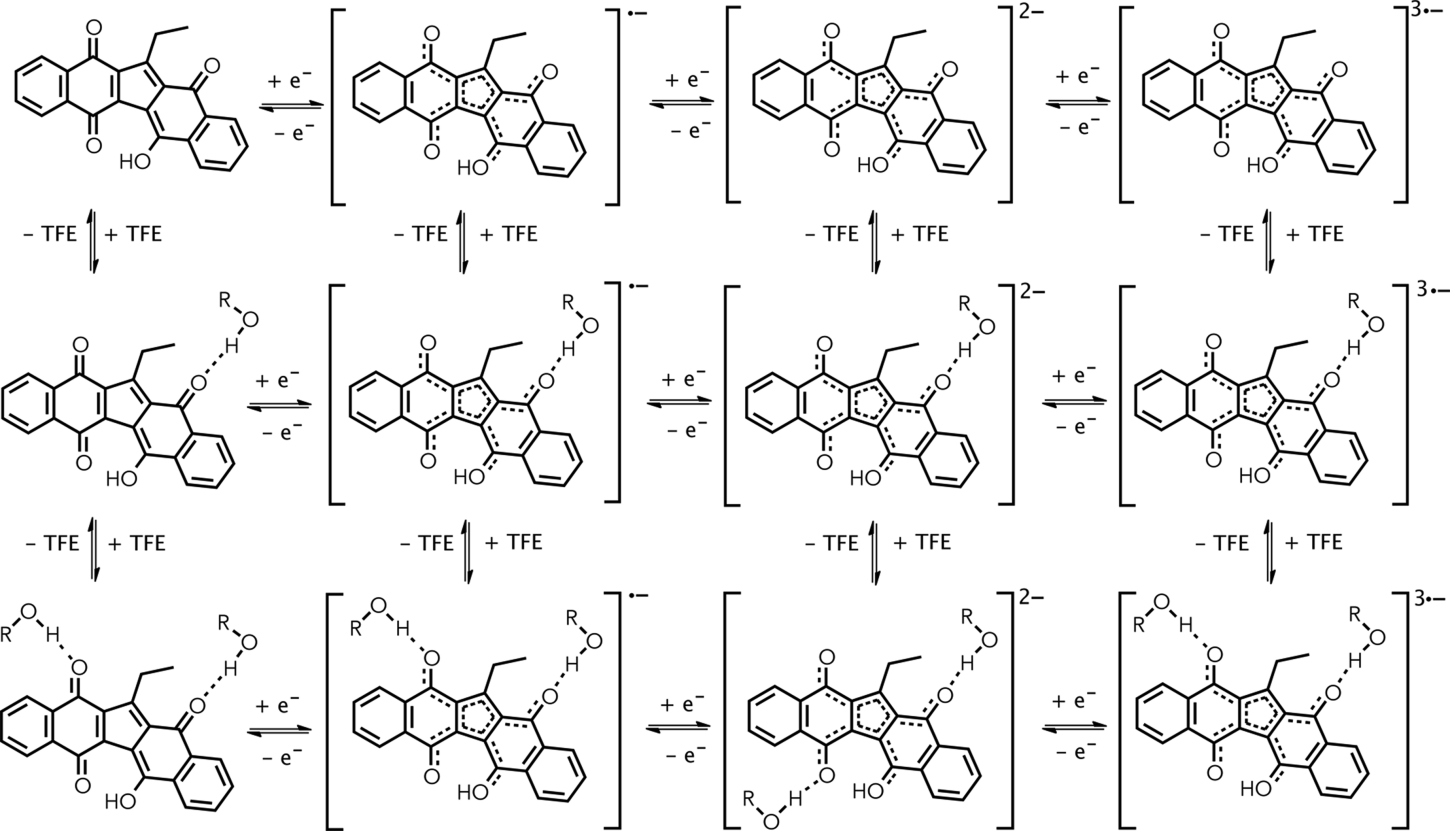
Electron Transfer Reactions and Hydrogen-Bonding Equilibria for KuQEt

As shown in Scheme 2, results indicate that the hydrogen bond is preferentially established with the carbonyl group opposite to the enol group functionality (indicated as carbonyl C) with a bond length of 1.82 Å. Calculated hydrogen bond lengths and O–H—O angles are in line with those typically observed for intermolecular hydrogen bonds occurring in small molecules.^25^ Notably, the TFE interaction with carbonyl oxygen (B) is just 0.15 kcal/mol higher in energy than that with (C), so probably the two KuQ[TFE] complexes are in equilibrium in solution. Considering KuQ[TFE]_2_ species, carbonyl oxygens (C) and (B) likely interact with TFE through H-bonds since the carbonyl (A)–TFE interaction is still disfavored (Table S1).

The same calculations have been performed for the monoanionic and the dianionic species to study TFE interactions during the entire electrochemical process. Indeed, one or two negative charges are added on KuQEt, respectively.

Again, KuQ^·^^–^ coordination through carbonyl (B) and (C) is preferred to (A). Also, shorter bond lengths are measured (1.68–1.70 Å), probably because of the electronically enriched KuQEt core (Figure S8).^25^

Conversely, carbonyl (C) and (A) interactions with TFE molecules are preferred to (C) and (B) in the case of the dianionic KuQEt species, likely because of a strong increase in the negative charge density on carbonyl oxygen (A), upon the second reduction on the KuQEt core.

The H-bonded complexes are represented in Scheme 2. Here, electron transfer reactions are depicted horizontally, while the hydrogen-bonding equilibria are drawn vertically.

At this stage, it is still difficult to precisely assign each reduction process. Considering the charge distribution on the KuQEt skeleton (Figure S8), carbonyl oxygen (A) in the neutral species presents the highest negative charge density, so it can be speculated that the first reduction on carbonyl (B) or (C) is preferred. TFE interaction sites in KuQ^·^^–^ is in favor of such hypothesis.

Importantly, electron-spin density calculations on KuQ^·^^–^ species showed spin delocalization over the entire KuQuinone molecule (Figure S9). Such a feature is correlated with the general increase in the charge density on all the carbonyl oxygens upon reduction. However, carbonyl oxygen (A) being the one with the highest charge density, it can be assumed that also the second reduction process occurs on carbonyl (B) or (C).

### Spectroelectrochemical Study

**Figure 6:**
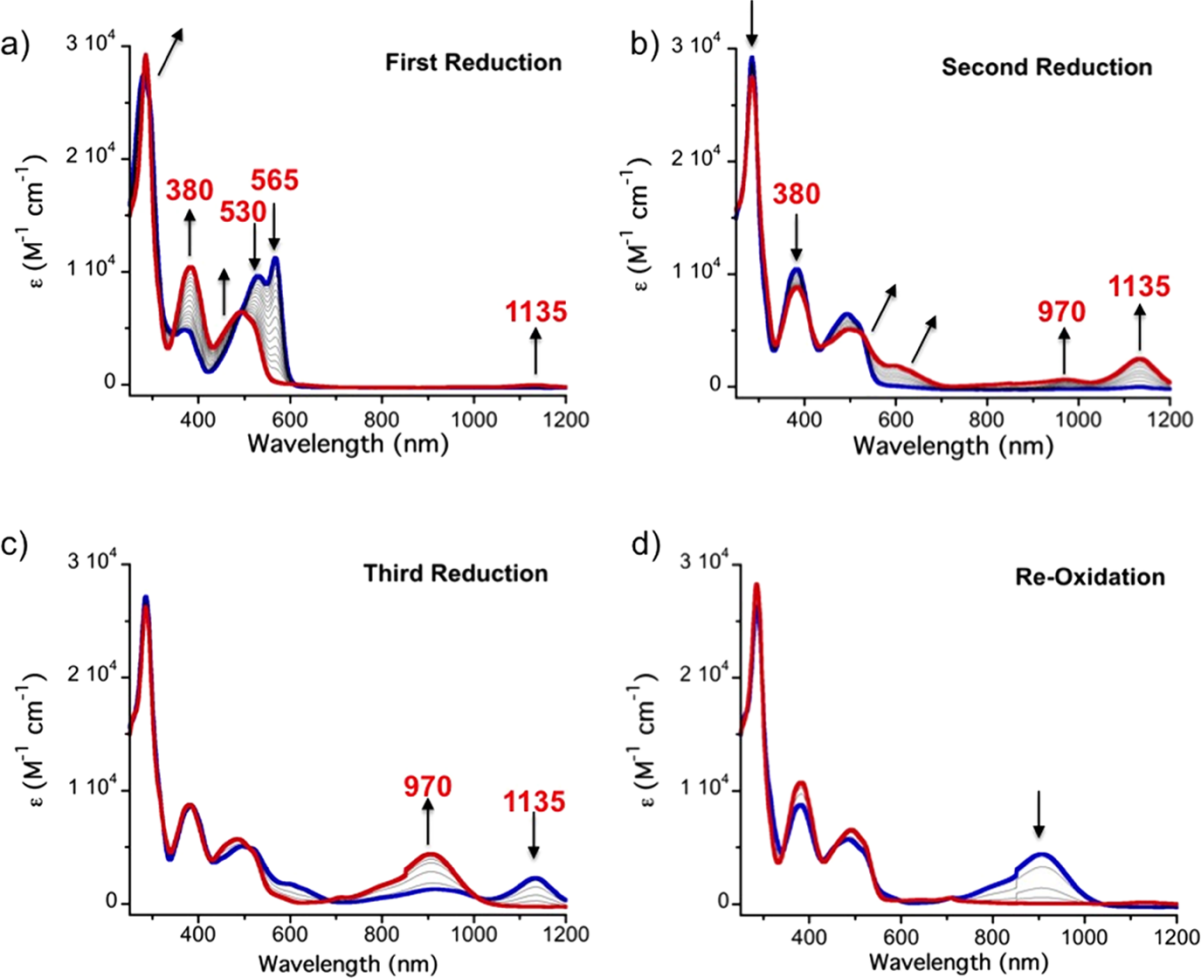
(a–d) UV–vis–NIR spectroelectrochemistry of KuQEt in CH_2_Cl_2_/TBAP (0.3 M). Platinum mesh as a working electrode and Ag/Ag^+^ as a pseudo-reference electrode. Initial spectrum (blue); final spectrum (red).

To further investigate the redox-active species generated upon the stepwise reduction of KuQuinone, UV–vis–NIR spectroelectrochemical experiments have been carried out in CH_2_Cl_2_/TBAP (0.3 M) (Figure 6).

At the beginning of the experiment, the first applied potential is slightly higher than *E*^(1)^_1/2_ and then it is gradually increased to allow the complete formation of the KuQ^·^^–^ species at the electrode surface. The experiment has been interrupted when no changes in the absorption spectra are observed. During the first reductive process (Figure 6a), a new broad band in the NIR region (ca. 1000 nm) appears, which can be ascribed to the KuQ^·^^–^ first excited state (HOMO → LUMO). Simultaneously, a decrease in the intensity of the bands at 530 and 565 nm is detected, together with an enhancement of a new wide band around 450 nm. In addition, the peak at 285 nm is gradually redshifted and increased in intensity. The obtained absorption spectrum is ascribed to monoanion radical species, i.e., KuQ^·^^–^. The shape of such a spectrum is comparable to the one of the deprotonated KuQuinone.^18^ Indeed, in Figure 6a, an isosbestic point is clearly visible, indicating that two species are involved in the process, namely, KuQ and KuQ^·^^–^. Similarly, an isosbestic point is observed in Figure 6b due to further reduction of KuQ^·^^–^ to KuQ^2–^. To observe this process, *E*^(2)^_1/2_ is used as the initial applied potential and it is gradually increased to obtain KuQ^2–^. Throughout the second reduction (Figure 6b), the new band at 1135 nm progressively increases its intensity, and a new broad band centered at 970 nm is detected. In the visible region, a red shift of the spectrum is observed, together with the formation of a broad band at 600 nm. Afterward, to analyze the third reduction process, the *E*^(3)^_1/2_ potential is initially applied and slowly increased. Interestingly, during the third reductive process (Figure 6c), the band at 1135 nm completely disappears, while the band at 970 nm is significantly enhanced. The UV region of the spectrum remains almost unchanged during the whole process. However, during the latter reduction step, broader spectra have been obtained and two isosbestic points can be detected, around 1000 and 700 nm. Therefore, the presence of more species involved in the process can be assumed.

To evaluate the reversibility of such processes, stepwise re-oxidation has been carried out (Figure 6d). However, since the final spectrum is that of KuQ^·^^–^ (Figure S10), the total re-oxidation to KuQ is not achieved. As already observed for CV measurements, such a result is due to the loss of reversibility of the first reduction process when using a Pt working electrode.

### Electrochemical and Spectroelectrochemical Study with Sc^3+^ Ions

As discussed above, quinone reduction potentials are strongly affected by several factors that can facilitate their reduction processes. Besides hydrogen bond donor additives, Lewis acids, such as Mg^2+^, Y^3+^, and Sc^3+^ ions, can also coordinate quinone carbonyl oxygens.^26,27^ Among these, Sc^3+^ ions demonstrated the strongest effect, decreasing the energetic level of the first reduction, thus enabling electron transfer processes that would not take place in their absence.^26−28^ Accordingly, the effect of Sc^3+^ on the KuQuinone redox behavior has been investigated. In the case of KuQuinones, DFT calculations performed using the B3LYP functional and LANL2DZ basis set revealed that in CH_2_Cl_2_, the exclusive coordination site for scandium ions is carbonyl oxygen (C).

**Figure 7:**
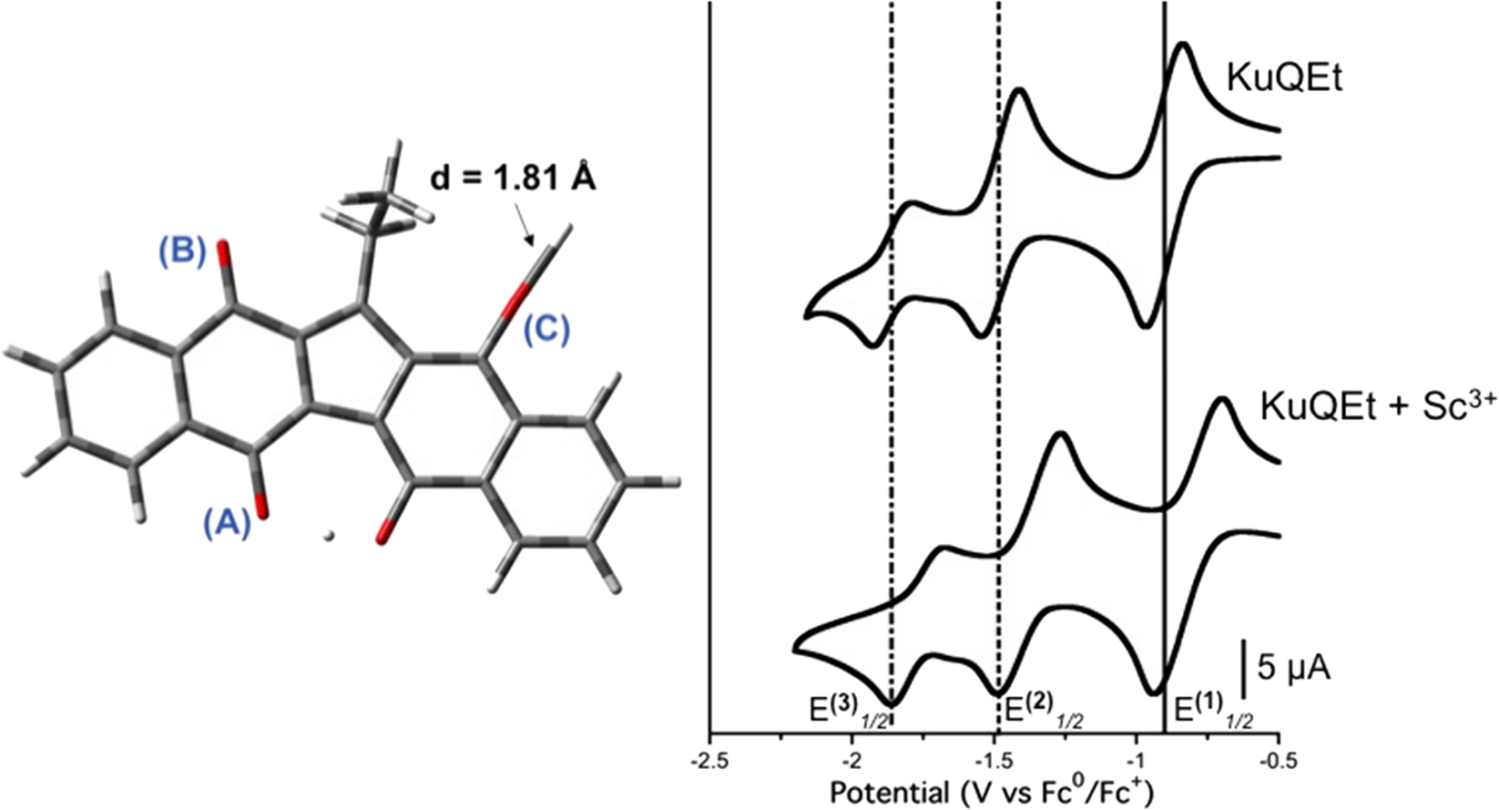
(Left) Optimized geometry in CH_2_Cl_2_ of the KuQ–Sc^3+^ complex with the B3LYP functional and LANL2DZ basis set. Color legend: gray, C; white (small), H; red, O; white (big), Sc. (Right) Cyclic voltammetry of KuQEt (1 mM) in the absence (top) and in the presence (bottom) of Sc^3+^ ions. CV recorded in CH_2_Cl_2_/TBAP (0.1 M), with glassy carbon as a working electrode.

Cyclic voltammetry experiments of KuQEt upon addition of Sc(OTf)_3_ have been carried out in CH_2_Cl_2_/TBAP (0.1 M). With respect to the standard system with no Sc^3+^, electrochemical data shows an important shift toward more positive potentials for all the reduction processes, without losing reversibility (Figure 7). Redox potentials are reported in Table 2.

**Table 2:** Redox Potential of KuQEt and KuQEt + Sc^3+^^a^

compound	*E*^(1)^_1/2_ (V)	Δ*E*^(1)^*_p_* (mV)	*E*^(2)^_1/2_ (V)	Δ*E*^(2)^*_p_* (mV)	*E*^(3)^_1/2_ (V)	Δ*E*^(3)^*_p_* (mV)
KuQEt	–0.90	100	–1.48	130	–1.86	150
KuQEt + Sc^3+^	–0.76	240	–1.37	220	–1.77	180

a Potential values are reported using the Fc^0^/Fc^+^ couple as an internal reference in CH_2_Cl_2_/TBAP (0.1 M).

**Figure 8:**
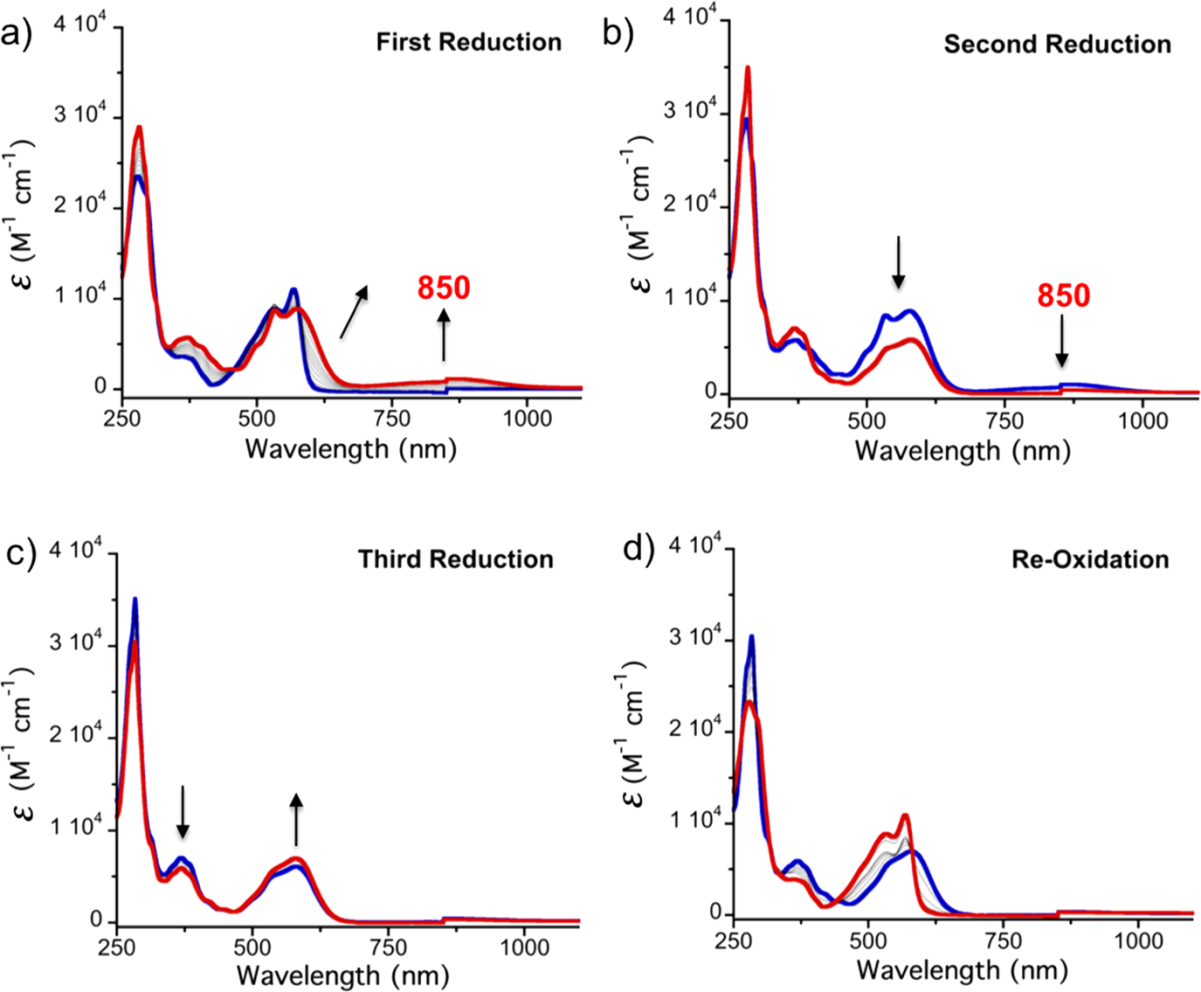
(a–d) UV–vis–NIR spectroelectrochemistry of KuQEt + Sc^3+^ in CH_2_Cl_2_/TBAP (0.3 M). Initial spectrum (blue); final spectrum (red).

The *E*_1/2_ shifts were 140, 110, and 90 mV for the first, second, and third reduction processes, respectively. Different from hydrogen bond donor interactions, in which the first reductive process is only slightly affected by TFE addition, Sc^3+^ coordination significantly promotes all the reduction processes. In particular, the first one is shifted about 140 mV, so KuQuinone radical anion formation is even more favorable in the presence of scandium. Such a strong effect is probably due to the direct Sc^3+^ coordination to carbonyl oxygen (C), as revealed by DFT calculations. Therefore, carbonyl group (C) is likely involved in the first reduction process. On the other hand, DFT calculations showed that Sc^3+^ coordination on oxygens (A) and (B) is prevented in CH_2_Cl_2_. Therefore, the presence of Sc^3+^ ions is still significant but less effective for the second and third reduction processes. UV–vis–NIR spectroelectrochemical experiments were carried out to evaluate the effect of Sc^3+^ ions on the spectroscopic features of the redox species during the stepwise reduction of KuQEt (Figure 8).

During the first reductive process (Figure 8a), a new band at 850 nm is observed, together with a shoulder at about 600 nm, probably due the interaction of the KuQ^·^^–^ with Sc^3+^ (Figure 8a, red trace). Such a spectroscopic profile is quite different from the one of KuQ^·^^–^ due to the scandium ion interactions. During the second reduction (Figure 8b), a general decrease in the intensity of the spectrum above 450 nm is observed. At the end of the third reduction step (Figure 8c), the band at 550 nm is increased, together with a decrease in the peak at 380 nm. In all the steps, one isosbestic point is observed, indicating that two species are involved in each reduction process, namely, scandium complexes of KuQ/KuQ^·^^–^, KuQ^·^^–^/KuQ^2–^, and KuQ^2–^/KuQ^3^^·^^–^. Remarkably, in comparison with the same experiments performed in the absence of Sc^3+^, well-defined spectra have been achieved; thus, scandium ions in solution promote KuQ’s full reduction to KuQ^3^^·^^–^, also overcoming diffusion issues previously observed.

The most interesting feature of this process is that upon re-oxidation, the distinctive absorption spectrum of KuQuinone in its enol form is obtained (Figure 8d). Such an effect is not observed in the absence of Sc^3+^, indicating the key role of such ions in promoting electroactive processes.

Therefore, scandium coordination to KuQuinone is a very efficient tool for favoring the three KuQs’ reduction processes, also warranting the reversibility of each redox step.

## Conclusions

In this work, a detailed investigation of KuQEt electrochemical and spectroelectrochemical features has been accomplished. Results showed that KuQuinones are characterized by three reversible reduction processes in CH_2_Cl_2_ using a glassy carbon working electrode. Such processes are attributed to the reduction of the three carbonyl groups in the structure. Remarkably, the first one is highly favored, occurring at potentials very close to 0 V. On the other hand, cyclic voltammetry measurements performed in DMF showed a quite different electrochemical profile. In fact, in DMF, deprotonation of the distinctive enol proton of KuQs occurs, thus generating the enolate species. The latter, being anionic, presents two broad reduction peaks negatively shifted in half-wave potentials with respect to the protonated compound. However, in the presence of selected additives in solution, it is possible to tune the redox behavior of KuQuinones. In fact, upon addition of TFA, enolate protonation occurs, thus leading to the classical voltammogram of KuQs, characterized by the three processes. Also, with TFA excess, reductions occur at even lower potentials than those in CH_2_Cl_2_, likely because of the established hydrogen bond between TFA excess and carbonyl oxygens. As a matter of fact, also cyclic voltammetry measurements performed in CH_2_Cl_2_ in the presence of TFE as a non-acid hydrogen bond donor show a positive shift of the half-wave potentials due to the TFE H-bond interaction with KuQ carbonyl moieties. Through the global equilibrium model, it was possible to estimate that one or two TFE molecules are hydrogen bonded to the KuQ^2–^ with *K*^(2)^_eq_ = 4 × 10^5^ M^–*m*^ with *m* = 1.5. DFT calculations allowed identifying the preferred interaction sites of TFE with KuQuinones. Also, taking into account the charge distribution on the KuQEt skeleton, it was possible to assign the first and the second reduction processes, which possibly occur on carbonyls (B) and (C). Such groups are also the preferred TFE interaction sites in KuQ^·^^–^, where the carbonyl (A) interaction is likely prevented because of the internal hydrogen bond occurring with the enol proton. Conversely, upon the second reduction step, the TFE interaction with carbonyls (A) and (C) occurs.

Sc^3+^ ions as Lewis Acid additives result crucial to observe an intense shift of the half-wave potential for the three processes. In particular, the most intense shift (+140 mV) has been observed for the first reduction process, which leads to a KuQuinone radical anion. Such an important effect is probably due to the preferred Sc^3+^ coordination to carbonyl oxygen (C) in CH_2_Cl_2_, meaning that carbonyl group (C) is the one involved in the first reduction process. The UV–vis–NIR spectroelectrochemical study allowed the characterization of the spectroscopic features of the KuQ reduced species.

In conclusion, a customizable redox chemistry of KuQs has been disclosed; in fact, it is feasible to specifically control their electrochemical behavior, thus allowing applications in sensors as well as in photoelectrochemical devices and donor–acceptor systems.

## Experimental Section

### Materials and Instruments

All commercial chemicals were purchased from Merck, with the highest degree of purity, and they were used without any further purification, with the exception of the supporting electrolytes used for electrochemistry. TFAB, TBAPF_6_, and TBAP were crystallized twice from ethyl acetate prior to use. Also, non-anhydrous dichloromethane was distilled and dried over calcium hydride. The electrochemical measurements were performed using a PalmSens potentiostat with PS-Trace software or a CH Instruments electrochemical analyzer. The UV–vis–NIR spectroelectrochemistry experiments were carried out with a Jasco-720 spectrophotometer coupled with a CH Instruments electrochemical analyzer. The absorption spectra were recorded with a UV–Vis 2450 Shimadzu spectrophotometer. ^1^H NMR experiments were carried out using a Bruker AM400 spectrometer operating at 400 MHz.

### Synthesis of 1-EthylKuQuinone (KuQEt)^14^

DMSO was kept over anhydrous K_2_CO_3_ overnight before use. In the general synthetic procedure, in a 50 mL round-bottom flask, 1 g (5.75 mmol) of 2-hydroxy-1,4-naphthoquinone, 2.5 g (8 mmol) of Cs_2_CO_3_, and 62 mg of sublimated ferrocene were added to 22 mL of DMSO. Then, 1.64 mL (12 mmol) of 1-bromobutane was added. The mixture was kept under stirring at 114 °C for 41 h in an oil bath, then diluted with 150 mL of dichloromethane, and filtered. The filtered solution was extracted with brine solution (2 × 300 mL), dried over Na_2_SO_4_, and filtered. The brownish powder obtained after solvent evaporation was purified by a chromatography column (SiO_2_ and CH_2_Cl_2_ as eluents). The isolated purple powder was repetitively crystallized from dichloromethane-hexane (yield = 15%).

^1^H NMR (CDCl_3_, 400 MHz): 18.141 (s, 1H), 8.268–8.222 (m, 4H), 7.790–7.689 (m, 4H), 3.468 (q, *J* = 7.50 Hz, 2H), 1.297 (t, *J* = 7.50 Hz, 3H).

### Cyclic Voltammetry

All the electrochemical experiments were carried out by cyclic voltammetry. Analyzed solutions were degassed by bubbling nitrogen before the measurements. Cyclic voltammetries were recorded from −1.5 to 0 V at a scan rate of 100 mV/s.

KuQuinones’ redox behavior has been evaluated by changing the supporting electrolyte and solvent nature. Tetrabutylammonium perchlorate (TBAP), tetrabutylammonium hexafluorophosphate (TBAPF_6_), and tetrabutylammonium tetrakis(pentafluorophenyl)borate (TFAB) have been used, with CH_2_Cl_2_ as the solvent.

Experiments performed changing supporting electrolytes were carried out using a three-electrode scheme, with a calomel electrode as a reference (SCE, Amel electrochemistry 303/SCG/6 electrode), a platinum disk as a working electrode, and a platinum wire as a counter electrode, dissolving a small amount of KuQ in a 0.1 M solution of TFAB, TBAPF_6_, or TBAP in anhydrous CH_2_Cl_2_. SCE was calibrated before and after each set of experiment with ferrocene (*E*_ox_ vs SCE = 0.40 V).

Cyclic voltammetry experiments in spectroscopic-grade DMF were carried out with an aged solution of KuQ (1 mM)/TBAP (0.1 M), with subsequent additions of a concentrated TFA solution. After each TFA addition, a voltammogram was recorded using a calomel electrode as a reference, a glassy carbon disk as a working electrode, and a platinum wire as a counter electrode.

Hydrogen bond donor experiments were conducted in KuQ (2 mM)/TBAP (0.1 M) in anhydrous CH_2_Cl_2_, with stepwise addition of a concentrated TFE solution. After each TFE addition, a voltammogram was recorded using a calomel electrode as a reference, a glassy carbon disk as a working electrode, and a platinum wire as a counter electrode.

Cyclic voltammetry in the presence of Sc^3+^ ions was carried out using a three-electrode scheme with a platinum wire working electrode, counter electrode, and Ag/Ag^+^ pseudo-reference electrode. Before analysis with the pseudo-reference electrode, the electrochemical cell was calibrated using an Fc^0^/Fc^+^ couple. Measurements were performed adding a known amount of Sc(OTf)_3_ in KuQ (1 mM)/TBAP (0.1 M) solution in CH_2_Cl_2_.

### UV–Vis Absorption Spectra

Spectroscopic-grade dichloromethane and DMF have been used to record UV–vis absorption spectra in quartz cuvettes. To perform the analysis in the presence of TFA or TFE, different aliquots of such additives were added to a solution of KuQEt in dichloromethane.

### Spectroelectrochemical Experiments

UV–vis–NIR spectroelectrochemical experiments both in the presence and absence of Sc^3+^ ions have been carried in a three-electrode configuration with Ag/Ag^+^ as a pseudo-reference electrode, platinum mesh as a working electrode, and a platinum wire as a counter electrode. Before analysis, the solution was degassed by bubbling nitrogen. Measurements were performed by dissolving a small amount of KuQ in a 0.3 M TBAP solution in CH_2_Cl_2_.

In UV–vis–NIR spectroelectrochemistry in the presence of Sc^3+^ ions, a small amount of KuQ was dissolved in a 0.3 M TBAP solution in dichloromethane and then an amount of Sc(OTf)_3_ was added.

### DFT Calculations

DFT calculations for geometry optimization were performed using Gaussian 16 rev. A.03.^29^ The B3LYP functional has been used with the 6-31G+(d,p) basis set for geometry optimization of KuQ, KuQ^·^^–^, and KuQ^2–^ in the vacuum and then in dichloromethane using the polarizable continuum model in this latter case. The interaction of KuQuinone (neutral or reduced species) with one or two TFE molecules has been modeled, applying correction for basis set superposition error (BSSE) and using the counterpoise. DFT calculations using the B3LYP functional and LANL2DZ basis set have been performed for geometry optimization of KuQ–Sc^3+^ complexes.

## Supporting Information Available

^1^H NMR, cyclic voltammetries, UV–vis spectra, and DFT calculations (PDF)

## References

[ref1] Lambreva M.; Russo D.; Polticelli F.; Scognamiglio V.; Antonacci A.; Zobnina V.; Campi G.; Rea G. Structure/Function/Dynamics of Photosystem II Plastoquinone Binding Sites. Curr. Protein Pept. Sci. 2014, 15, 285–295. 10.2174/1389203715666140327104802. 24678671 PMC4030317

[ref2] Han C.; Li H.; Shi R.; Zhang T.; Tong J.; Li J.; Li B. Organic Quinones towards Advanced Electrochemical Energy Storage: Recent Advances and Challenges. J. Mater. Chem. A 2019, 7, 23378–23415. 10.1039/C9TA05252F.

[ref3] Gurkan B.; Simeon F.; Hatton T. A. Quinone Reduction in Ionic Liquids for Electrochemical CO_2_ Separation. ACS Sustainable Chem. Eng. 2015, 3, 1394–1405. 10.1021/acssuschemeng.5b00116.

[ref4] Yin W.; Grimaud A.; Azcarate I.; Yang C.; Tarascon J.-M. Electrochemical Reduction of CO_2_ Mediated by Quinone Derivatives: Implication for Li–CO_2_ Battery. J. Phys. Chem. C 2018, 122, 6546–6554. 10.1021/acs.jpcc.8b00109.

[ref5] Liu Y.; Ye H.-Z.; Diederichsen K. M.; Van Voorhis T.; Hatton T. A. Electrochemically Mediated Carbon Dioxide Separation with Quinone Chemistry in Salt-Concentrated Aqueous Media. Nat. Commun. 2020, 11, 2278 10.1038/s41467-020-16150-7. 32385274 PMC7211026

[ref6] Silva T. L.; de Azevedo M. d. L. S. G.; Ferreira F. R.; Santos D. C.; Amatore C.; Goulart M. O. F. Quinone-Based Molecular Electrochemistry and Their Contributions to Medicinal Chemistry: A Look at the Present and Future. Curr. Opin. Electrochem. 2020, 24, 79–87. 10.1016/j.coelec.2020.06.011.

[ref7] Hui Y.; Chng E. L. K.; Chng C. Y. L.; Poh H. L.; Webster R. D. Hydrogen-Bonding Interactions between Water and the One- and Two-Electron-Reduced Forms of Vitamin K1: Applying Quinone Electrochemistry To Determine the Moisture Content of Non-Aqueous Solvents. J. Am. Chem. Soc. 2009, 131, 1523–1534. 10.1021/ja8080428. 19132833

[ref8] Guin P. S.; Das S.; Mandal P. C. Electrochemical Reduction of Quinones in Different Media: A Review. Int. J. Electrochem. 2011, 2011, 1–22. 10.4061/2011/816202.

[ref9] Cramer W. A., Knaff D. B. Energy Transduction in Biological Membranes: A Textbook of Bioenergetics; Eds. Cramer W. A.; Knaff D. B., Springer Advanced Texts in Chemistry; Springer-Verlag: New York, 1990**;** 10.1007/978-1-4612-3220-9.

[ref10] Gupta N.; Linschitz H. Hydrogen-Bonding and Protonation Effects in Electrochemistry of Quinones in Aprotic Solvents. J. Am. Chem. Soc. 1997, 119, 6384–6391. 10.1021/ja970028j.

[ref11] Gómez M.; González F. J.; González I. A Model for Characterization of Successive Hydrogen Bonding Interactions with Electrochemically Generated Charged Species. The Quinone Electroreduction in the Presence of Donor Protons. Electroanalysis 2003, 15, 635–645. 10.1002/elan.200390080.

[ref12] Tessensohn M. E.; Lee M.; Hirao H.; Webster R. D. Measuring the Relative Hydrogen-Bonding Strengths of Alcohols in Aprotic Organic Solvents. ChemPhysChem 2015, 16, 160–168. 10.1002/cphc.201402693. 25418984

[ref13] Hui Y.; Chng E. L. K.; Chua L. P.-L.; Liu W. Z.; Webster R. D. Voltammetric Method for Determining the Trace Moisture Content of Organic Solvents Based on Hydrogen-Bonding Interactions with Quinones. Anal. Chem. 2010, 82, 1928–1934. 10.1021/ac9026719. 20143888

[ref14] Coletti A.; Lentini S.; Conte V.; Floris B.; Bortolini O.; Sforza F.; Grepioni F.; Galloni P. Unexpected One-Pot Synthesis of Highly Conjugated Pentacyclic Diquinoid Compounds. J. Org. Chem. 2012, 77, 6873–6879. 10.1021/jo300985x. 22834705

[ref15] Arnò B.; Coletta A.; Tesauro C.; Zuccaro L.; Fiorani P.; Lentini S.; Galloni P.; Conte V.; Floris B.; Desideri A. A Small Organic Compound Enhances the Religation Reaction of Human Topoisomerase I and Identifies Crucial Elements for the Religation Mechanism. Biosci. Rep. 2013, 33, e00025 10.1042/BSR20120118. 23368812 PMC3590572

[ref16] Sabuzi F.; Armuzza V.; Conte V.; Floris B.; Venanzi M.; Galloni P.; Gatto E. KuQuinones: A New Class of Quinoid Compounds as Photoactive Species on ITO. J. Mater. Chem. C 2016, 4, 622–629. 10.1039/C5TC03363B.

[ref17] Bonomo M.; Sabuzi F.; Di Carlo A.; Conte V.; Dini D.; Galloni P. KuQuinones as Sensitizers for NiO Based P-Type Dye-Sensitized Solar Cells. New J. Chem. 2017, 41, 2769–2779. 10.1039/C6NJ03466G.

[ref18] Sabuzi F.; Lentini S.; Sforza F.; Pezzola S.; Fratelli S.; Bortolini O.; Floris B.; Conte V.; Galloni P. KuQuinones Equilibria Assessment for Biomedical Applications. J. Org. Chem. 2017, 82, 10129–10138. 10.1021/acs.joc.7b01602. 28872314

[ref19] Volpato G. A.; Marasi M.; Gobbato T.; Valentini F.; Sabuzi F.; Gagliardi V.; Bonetto A.; Marcomini A.; Berardi S.; Conte V.; Bonchio M.; Caramori S.; Galloni P.; Sartorel A. Photoanodes for Water Oxidation with Visible Light Based on a Pentacyclic Quinoid Organic Dye Enabling Proton-Coupled Electron Transfer. Chem. Commun. 2020, 56, 2248–2251. 10.1039/C9CC09805D. 31993616

[ref20] Onuki M.; Ota M.; Otokozawa S.; Kamo S.; Tomoshige S.; Tsubaki K.; Kuramochi K. Dimerizations of 2-Bromo-3-Methyl-1,4-Naphthoquinone and 2-Methyl-1,4-Naphthoquinone in Tetra-n-Butylammonium Bromide. Tetrahedron 2020, 76, 130899 10.1016/j.tet.2019.130899.

[ref21] Frontana C. E.; Gómez M.; González I. Intra vs Intermolecular Association Processes in the Radical Anions of B-Hydroxyquinones. Influence on the Structural Properties of the Radical Anion of Julgone. ECS Trans. 2007, 3, 37–44. 10.1149/1.2753289.

[ref22] Chen B.; Neumann R. On the effect of ion pairing of Keggin type polyanions with quaternary ammonium cations on redox potentials in organic solvents. Phys. Chem. Chem. Phys. 2016, 18, 22487–22493. 10.1039/C6CP03315F. 27465599

[ref23] Gómez-Gil J. M.; Laborda E.; Gonzalez J.; Molina A.; Compton R. G. Electrochemical and Computational Study of Ion Association in the Electroreduction of PW_12_O_40_^3–^. J. Phys. Chem. C 2017, 121, 26751–26763. 10.1021/acs.jpcc.7b07073.

[ref24] Morris R. H. Brønsted–Lowry Acid Strength of Metal Hydride and Dihydrogen Complexes. Chem. Rev. 2016, 116, 8588–8654. 10.1021/acs.chemrev.5b00695. 26963836

[ref25] Herschlag D.; Pinney M. M. Hydrogen Bonds: Simple After All. Biochemistry 2018, 57, 3338–3352. 10.1021/acs.biochem.8b00217. 29678112

[ref26] Fukuzumi S.; Okamoto K.; Imahori H. Thermal Intramolecular Electron Transfer in a Ferrocene-Naphthoquinone Linked Dyad Promoted by Metal Ions. Angew. Chem., Int. Ed. 2002, 41, 620–622. 10.1002/1521-3773(20020215)41:4<620::AID-ANIE620>3.0.CO;2-D. 12783556

[ref27] Yuasa J.; Yamada S.; Fukuzumi S. Accelerating and Decelerating Effects of Metal Ions on Electron-Transfer Reduction of Quinones as a Function of Temperature and Binding Modes of Metal Ions to Semiquinone Radical Anions. Chem. – Eur. J. 2008, 14, 1866–1874. 10.1002/chem.200701420. 18069714

[ref28] Sabuzi F.; Coletti A.; Pomarico G.; Floris B.; Galloni P.; Conte V. Modulating Electron Transfer in Ferrocene-Naphthoquinone Dyads: New Insights in Parameters Influencing ET Efficiency. J. Organomet. Chem. 2019, 885, 49–58. 10.1016/j.jorganchem.2019.02.001.

[ref29] Frisch M. J.; Trucks G. W.; Schlegel H. B.; Scuseria G. E.; Robb M. A.; Cheeseman J. R.; Scalmani G.; Barone V.; Petersson G. A.; Nakatsuji H.; Gaussian 16, Revision A.03. *Gaussian 16, Revision A.03* 2016, Gaussian, Inc.: Wallingford, CT, USA.

