# Supplementary material for: Unveiling KuQuinone Redox Species: An Electrochemical and Computational Cross Study
Source: J Org Chem. 2021 Apr 7;86(8):5680–9. doi: 10.1021/acs.joc.1c00165 (PMC8154575; doi:10.1021/acs.joc.1c00165)
Supplement: Supplementary file 1 — jo1c00165_si_001.pdf [file jo1c00165_si_001.pdf]

Supporting Information

Unveiling KuQuinone redox species: an electrochemical and computational cross study

Francesca Valentini,[a,c] Federica Sabuzi,[a] Valeria Conte,[a] Victor N. Nemykin,[b,c] Pierluca Galloni*[a]

[a] Department of Chemical Science and Technologies, University of Rome Tor Vergata, Via della Ricerca Scientifica, 00133, Rome (Italy)

[b] Department of Chemistry, University of Tennessee, Knoxville, Tennessee 37996, (United States)

[c] Department of Chemistry, University of Manitoba, R3T 2N2, Winnipeg, Manitoba, (Canada)

Corresponding author's email address: galloni@scienze.uniroma2.it (P.G.)

Table of contents

Figure S1.	^{1}H NMR of 1-ethylKuQuinone (KuQEt)	S3
Figure S2.	UV-vis absorption spectrum of KuQEt in different solvents	S3
Figure S3.	UV-vis absorption spectra of one-day aged solution of KuQEt in DMF and with the addition of 1.5 eq of TFA	S4
Figure S4.	Cyclic Voltammetry titration for KuQEt in DMF at different TFA concentrations	S4
Figure S5.	Comparison between Platinum disk and glassy carbon working electrodes in Cyclic voltammetry of KuQEt 2 mM in CH_2Cl_2/TBAP 0.1 M	S5
Figure S6.	ΔE1/2 vs. [TFE] plot	S5
Figure S7.	UV-vis absorption spectra titration for KuQEt in CH_2Cl_2 at different TFE concentrations	S6
Figure S8.	Charge density distribution in KuQ, $KuQ^{\bullet-}$ and KuQ^{2-}	S7
Figure S9.	Spin-delocalization in $KuQ^{\bullet-}$ predicted with DFT calculations	S8
Figure S10.	UV-vis-NIR absorption spectra of KuQEt in CH_2Cl_2 after the first reduction and the re-oxidation	S8
Table S1	Calculated total energies with B3LYP functional, 6-31G+(d,p) basis set	S9
	Optimized cartesian coordinates (in Angstroms)	S10

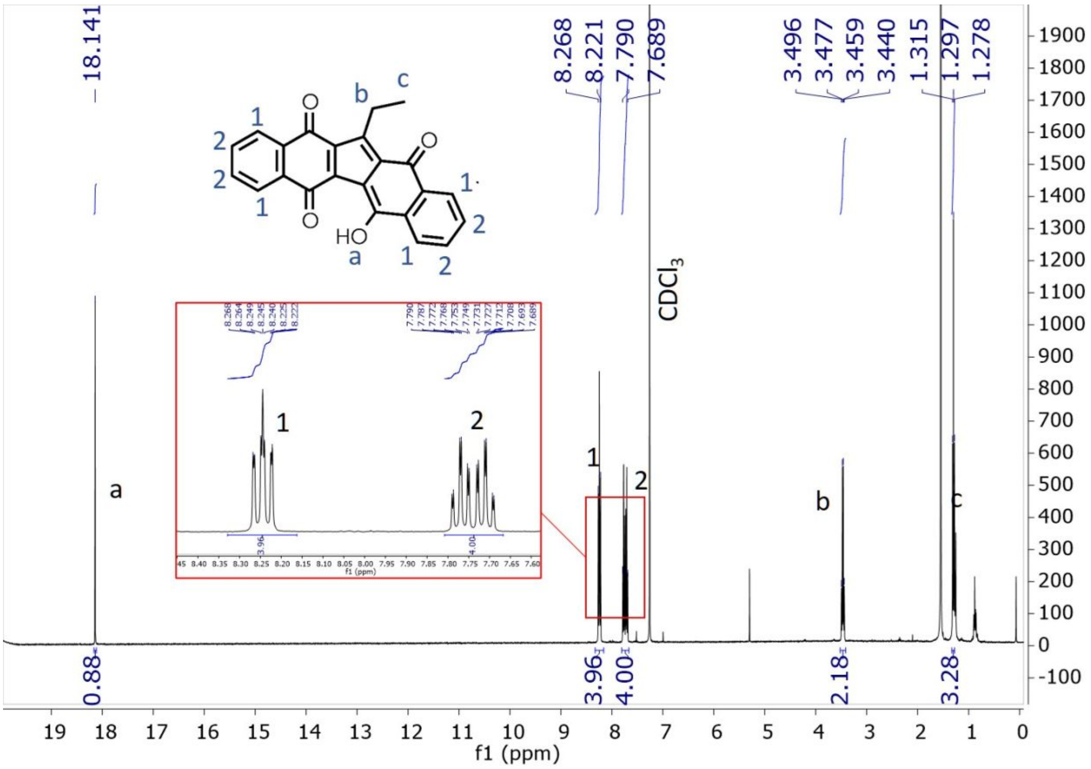

Figure S1. ^{1}H-NMR spectrum of 1-ethylKuQuinone in $CDCl_3$ (400 MHz).

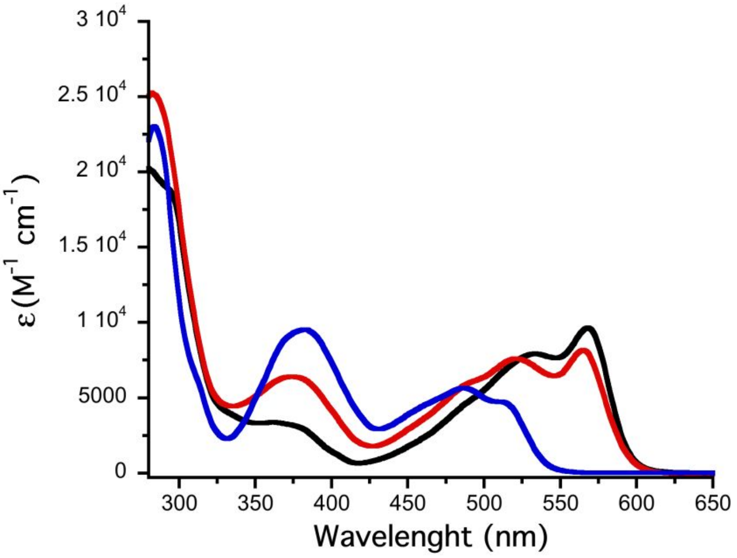

Figure S2. Absorption spectra of KuQEt 1 mM in: CH_2Cl_2 (black), anhydrous DMF (red) and one-day aged KuQEt solution in DMF (blue).

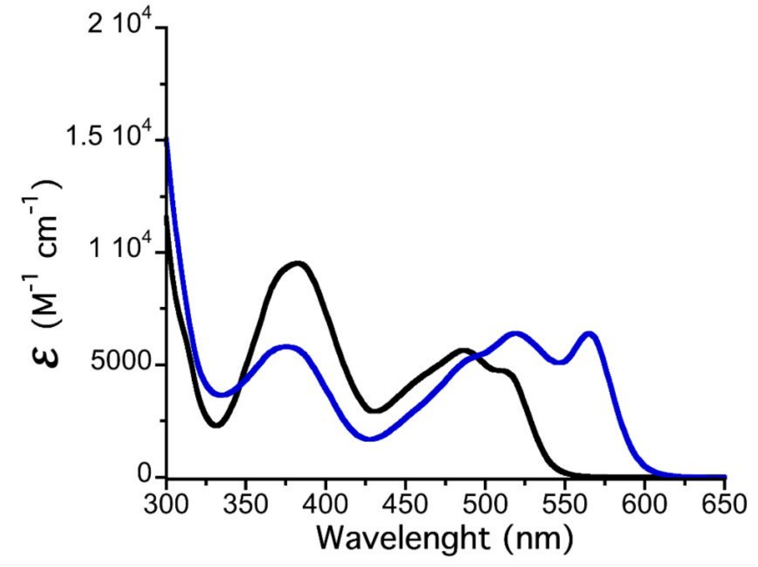

Figure S3. Absorption spectra of one-day aged solution of KuQEt 1 mM in DMF (black) and with the addition of 1.5 eq. of TFA (blue).

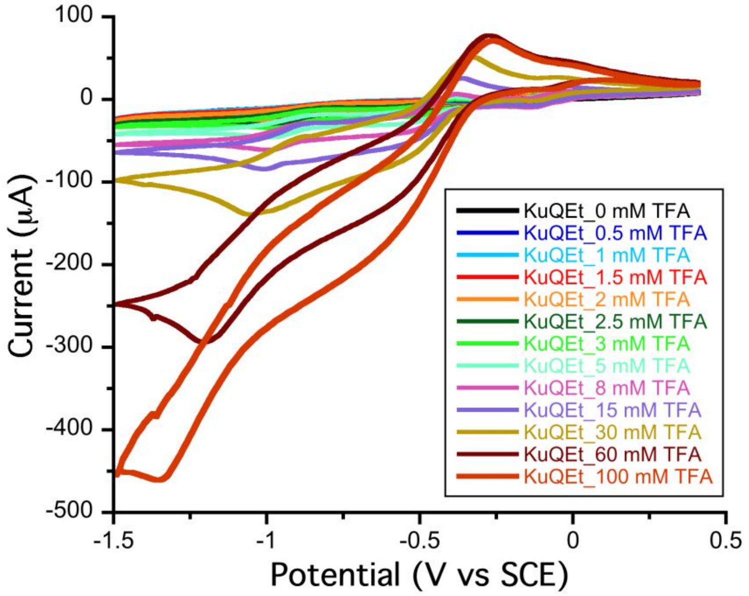

Figure S4. Cyclic voltammetry of KuQEt 1mM with stepwise addition of TFA (up to 100mM) in DMF/TBAP 0.1 M.

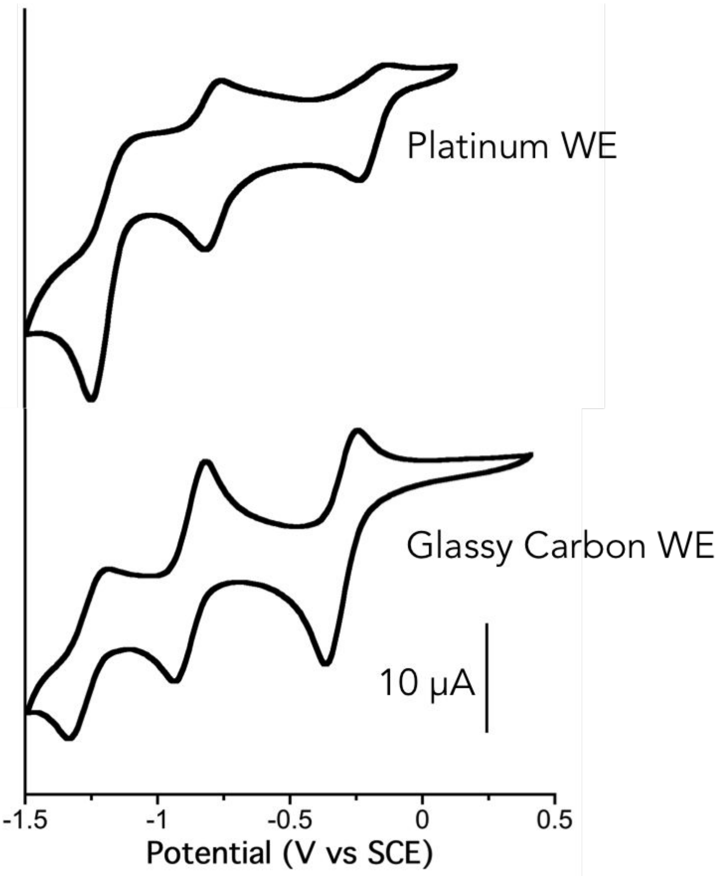

Figure S5. Cyclic Voltammetry of KuQEt 2 mM/TBAP 0.1 M in CH_2Cl_2 using as working electrode (top) Platinum disk (bottom) Glassy carbon.

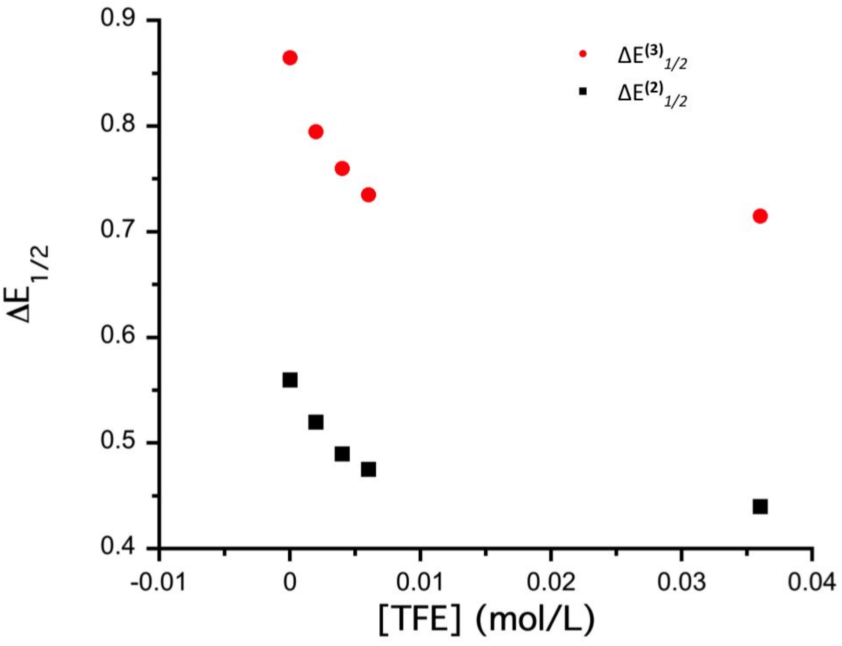

Figure S6. $\Delta E_{1/2}$ *vs*. [TFE] plot: [$\Delta E^{(3)}_{1/2} = E^{(3)}_{1/2} - E^{(1)}_{1/2}$] (red); [$\Delta E^{(2)}_{1/2} = E^{(2)}_{1/2} - E^{(1)}_{1/2}$] (black).

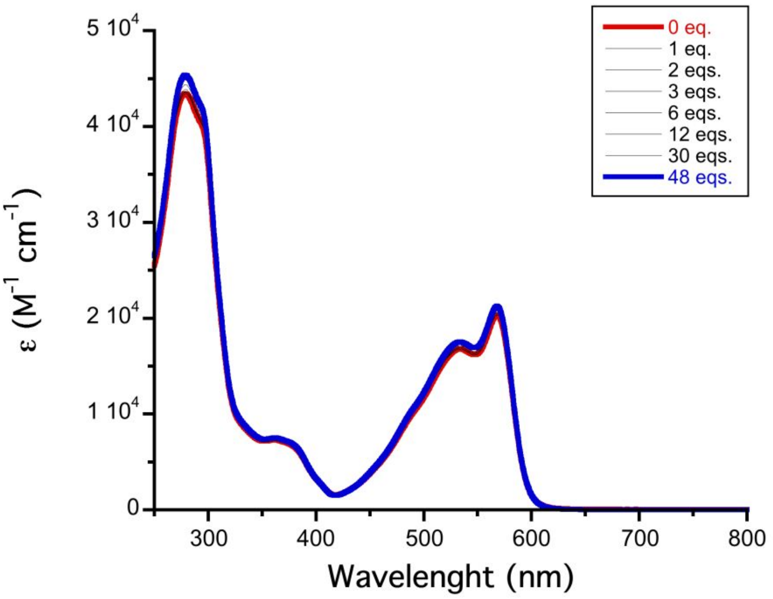

Figure S7. UV-vis Absorption Spectra of KuQEt 0.5 mM in CH_2Cl_2 with stepwise addition of TFE up to 24 mM. Cuvette length 1 mm.

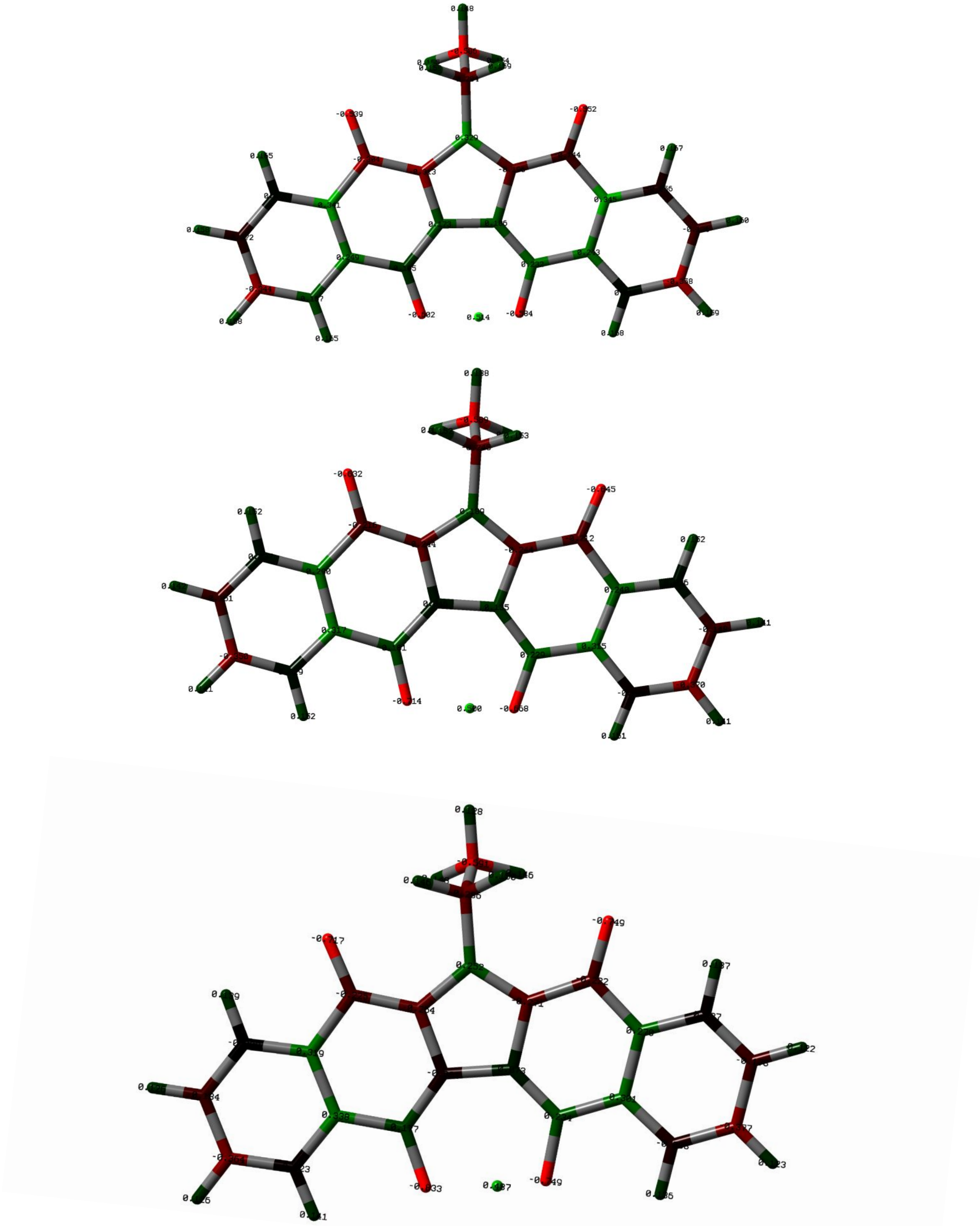

Figure S8. Charge density distribution in KuQ (top), $KuQ^{\cdot -}$ (centre) and KuQ^{2-} (bottom). DFT calculations performed with B3LYP functional and 6-31G+(d,p) basis set (solvent: DCM). Color legend: green: positive; red: negative.

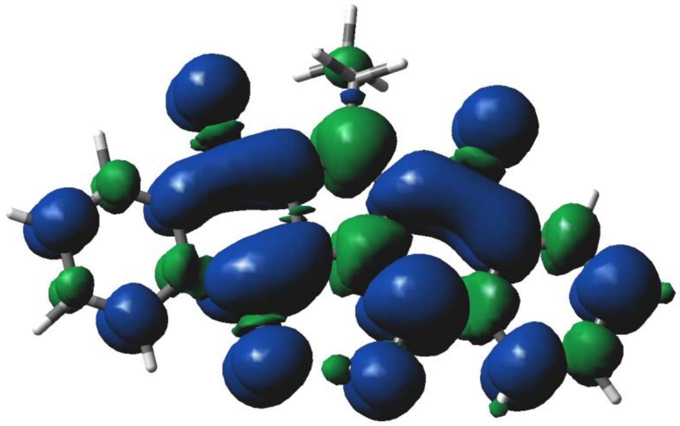

Figure S9. Spin-delocalization in $KuQ^{•-}$ predicted with DFT calculations (B3LYP functional and 6-31G+(d,p) basis set; solvent: DCM).

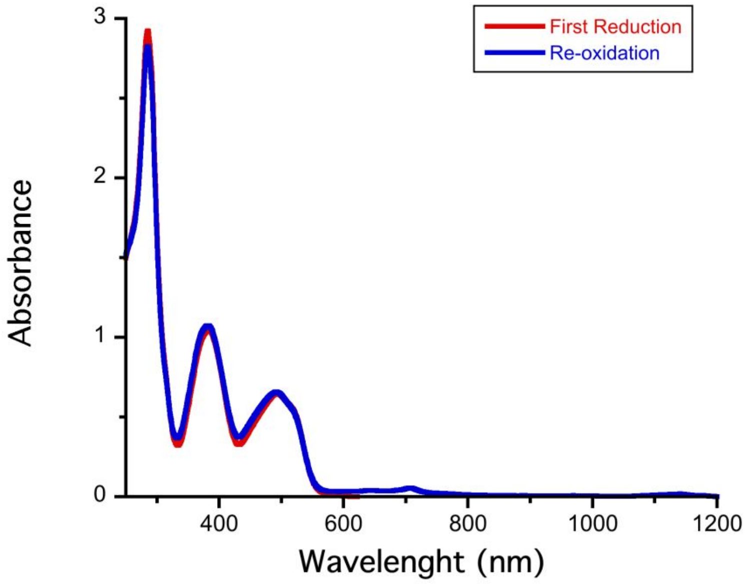

Figure S10. KuQEt Uv-vis-NIR spectra comparison between the final spectrum at the end of the first reduction (red trace) and after the re-oxidation (blue trace) in CH_2Cl_2

Table S1: Calculated total energies with B3LYP functional, 6-31G+(d,p) basis set.

	**solvent**	**Total energy (Hartree)**	**ΔE (kcal/mol)**
KuQ	*vacuum*	-1185.880516	-
KuQ	DCM	-1185.890212	-
[KuQ][TFE_A]	*vacuum*	-1638.685281	2.22
[KuQ][TFE_B]	*vacuum*	-1638.688536	0.18
[KuQ][TFE_C]	*vacuum*	-1638.688822	0
[KuQ][TFE_A]	DCM	-1638.699606	1.59
[KuQ][TFE_B]	DCM	-1638.701895	0.15
[KuQ][TFE_C]	DCM	-1638.702141	0
[KuQ][$2TFE_{BC}$]	*vacuum*	-2091.496547	-
[KuQ][$2TFE_{BC}$]	DCM	-2091.513639	-

	**solvent**	**Total energy (Hartree)**	**ΔE (kcal/mol)**
$KuQ^{\bullet-}$	*vacuum*	-1185.977692	-
$KuQ^{\bullet-}$	DCM	-1186.037045	-
[$KuQ^{\bullet-}$][TFE_A]	*vacuum*	-1638.794009	1.70
[$KuQ^{\bullet-}$][TFE_B]	*vacuum*	-1638.796092	0.40
[$KuQ^{\bullet-}$][TFE_C]	*vacuum*	-1638.796726	0
[$KuQ^{\bullet-}$][TFE_A]	DCM	-1638.851358	1.07
[$KuQ^{\bullet-}$][TFE_B]	DCM	-1638.852521	0.34
[$KuQ^{\bullet-}$][TFE_C]	DCM	-1638.853069	0
[$KuQ^{\bullet-}$][$2TFE_{BC}$]	*vacuum*	-2091.613441	-
[$KuQ^{\bullet-}$][$2TFE_{BC}$]	DCM	-2091.667937	-

	**solvent**	**Total energy (Hartree)**	**ΔE (kcal/mol)**
KuQ^{2-}	*vacuum*	-1185.937687	-
KuQ^{2-}	DCM	-1186.142285	-
[KuQ^{2-}][TFE_A]	*vacuum*	-1638.772153	0.51
[KuQ^{2-}][TFE_B]	*vacuum*	-1638.771095	1.17
[KuQ^{2-}][TFE_C]	*vacuum*	-1638.772964	0
[KuQ^{2-}][TFE_A]	DCM	immaginary freq.	-
[KuQ^{2-}][TFE_B]	DCM	-1638.962066	0.86
[KuQ^{2-}][TFE_C]	DCM	-1638.963439	0
[KuQ^{2-}][$2TFE_{BC}$]	*vacuum*	-2091.602391	1.52
[KuQ^{2-}][$2TFE_{AC}$]	*vacuum*	-2091.604811	0
[KuQ^{2-}][$2TFE_{BC}$]	DCM	immaginary freq	-
[KuQ^{2-}][$2TFE_{BC}$]	DCM	-2091.784411	0

Optimized cartesian coordinates (in Angstroms)

- Geometry optimization of **KuQ** in the *vacuum* (B3LYP functional 6-31G+(d,p) basis set)

```
Energy -1185.880516 Hartree
Stoichiometry    C23H14O4
 Framework group  C1[X(C23H14O4)]
 Deg. of freedom   117
 Full point group                C1      NOp   1
 Largest Abelian subgroup        C1      NOp   1
 Largest concise Abelian subgroup C1      NOp   1
```

Standard orientation:

Center Number	Atomic Number	Atomic Type	Coordinates (Angstroms) X	Y	Z
1	6	0	0.716012	-0.375433	-0.019919
2	6	0	1.135129	0.965033	-0.129820
3	6	0	-0.719042	-0.378354	-0.018523
4	6	0	2.559628	1.315405	-0.159394
5	6	0	1.627506	-1.486807	0.058563
6	6	0	-1.143865	0.987060	-0.128692
7	6	0	3.523777	0.159693	-0.089562
8	8	0	2.973600	2.472401	-0.231904
9	6	0	-0.012213	1.822195	-0.204135
10	6	0	3.077123	-1.178016	0.017926
11	8	0	1.233087	-2.679216	0.154773
12	6	0	4.895442	0.423419	-0.126815
13	6	0	4.008711	-2.222090	0.085533
14	6	0	5.818073	-0.622746	-0.060377
15	1	0	5.218388	1.456010	-0.207823
16	6	0	5.375299	-1.945442	0.046125
17	1	0	3.644422	-3.240384	0.168300
18	1	0	6.881929	-0.406706	-0.091180
19	1	0	6.093427	-2.758179	0.098406
20	6	0	-0.001714	3.317252	-0.325223
21	1	0	-0.907064	3.634302	-0.848106
22	1	0	0.873356	3.618966	-0.906406
23	6	0	-3.522301	0.173875	-0.087457
24	6	0	-3.066387	-1.163335	0.018031
25	1	0	-5.221908	1.458841	-0.202384
26	6	0	-4.894350	0.427548	-0.123424
27	6	0	-3.994175	-2.214158	0.084733
28	6	0	-5.361697	-1.943809	0.046051
29	6	0	-5.811915	-0.623875	-0.058478
30	1	0	-3.632524	-3.232938	0.166134
31	1	0	-6.074528	-2.761087	0.097515
32	1	0	-6.876912	-0.413770	-0.088770
33	6	0	-1.626433	-1.443296	0.055033
34	6	0	-2.564075	1.336507	-0.154670
35	8	0	-2.990650	2.490550	-0.220870
36	8	0	-1.264168	-2.695101	0.152803
37	1	0	-0.223873	-2.777301	0.164389
38	6	0	0.051140	4.019980	1.049219
39	1	0	0.957659	3.744339	1.596340
40	1	0	0.056060	5.106040	0.910962

41	1	0	-0.821418	3.759148	1.655868

Rotational constants (GHZ): 0.3808465 0.1306587 0.0983127

- Geometry optimization of **KuQ** in DCM (B3LYP functional 6-31G+(d,p) basis set)

Energy -1185.890212 Hartree
Stoichiometry C23H14O4
Framework group C1[X(C23H14O4)]
Deg. of freedom 117
Full point group C1 NOp 1
Largest Abelian subgroup C1 NOp 1
Largest concise Abelian subgroup C1 NOp 1

Standard orientation:

Center Number	Atomic Number	Atomic Type	Coordinates (Angstroms) X	Y	Z
1	6	0	0.714709	-0.375690	-0.020878
2	6	0	1.136183	0.965481	-0.130166
3	6	0	-0.718125	-0.377896	-0.019644
4	6	0	2.559096	1.313282	-0.158666
5	6	0	1.628550	-1.486766	0.057349
6	6	0	-1.144271	0.986391	-0.129178
7	6	0	3.523469	0.160804	-0.089300
8	8	0	2.972367	2.474849	-0.230349
9	6	0	-0.010920	1.821355	-0.204206
10	6	0	3.076559	-1.178216	0.017666
11	8	0	1.231552	-2.679340	0.152731
12	6	0	4.895728	0.422361	-0.126018
13	6	0	4.009084	-2.221505	0.085298
14	6	0	5.819530	-0.623797	-0.059548
15	1	0	5.226736	1.451936	-0.206844
16	6	0	5.376629	-1.946101	0.046564
17	1	0	3.650505	-3.241567	0.167709
18	1	0	6.882835	-0.407112	-0.090026
19	1	0	6.093787	-2.759145	0.098958
20	6	0	-0.000504	3.316522	-0.325280
21	1	0	-0.902774	3.633671	-0.853108
22	1	0	0.873852	3.618539	-0.907042
23	6	0	-3.522401	0.175442	-0.087241
24	6	0	-3.066683	-1.163246	0.018149
25	1	0	-5.231612	1.454575	-0.201768
26	6	0	-4.894952	0.426772	-0.122564
27	6	0	-3.994642	-2.213743	0.085489
28	6	0	-5.363090	-1.944641	0.047651
29	6	0	-5.813334	-0.625149	-0.056945
30	1	0	-3.637185	-3.233765	0.166830
31	1	0	-6.075017	-2.762094	0.099711
32	1	0	-6.877786	-0.414462	-0.086765
33	6	0	-1.628766	-1.443423	0.054122
34	6	0	-2.563240	1.333834	-0.154386
35	8	0	-2.987218	2.493348	-0.220279
36	8	0	-1.262757	-2.693175	0.151058
37	1	0	-0.222035	-2.771010	0.161761
38	6	0	0.048756	4.020782	1.048250

39	1	0	0.951475	3.741854	1.600185
40	1	0	0.055250	5.107069	0.911240
41	1	0	-0.823772	3.758940	1.654782

Rotational constants (GHZ): 0.3808372 0.1306376 0.0982983

- Geometry optimization of **KuQ + 1 TFE** (on carbonyl oxygen A) in the *vacuum* (B3LYP functional 6-31G+(d,p) basis set, BSSE and counterpoise correction)

Energy -1638.685281 Hartree
Stoichiometry C25H17F3O5
Framework group C1[X(C25H17F3O5)]
Deg. of freedom 144
Full point group C1 NOp 1
Largest Abelian subgroup C1 NOp 1
Largest concise Abelian subgroup C1 NOp 1
Standard orientation:

Center Number	Atomic Number	Atomic Type	Coordinates (Angstroms) X	Y	Z
1	6	0	-0.478387	-0.835889	-0.165954
2	6	0	-1.064191	-2.068256	0.186458
3	6	0	-1.522072	0.154907	-0.136288
4	6	0	-0.266842	-3.298595	0.251000
5	6	0	0.919380	-0.687084	-0.468135
6	6	0	-2.730966	-0.518111	0.238974
7	6	0	1.198387	-3.149623	-0.055250
8	8	0	-0.742486	-4.398776	0.529735
9	6	0	-2.460777	-1.884624	0.446748
10	6	0	1.767205	-1.899844	-0.404754
11	8	0	1.413822	0.438147	-0.776810
12	6	0	2.009671	-4.284474	0.002888
13	6	0	3.138189	-1.820078	-0.689554
14	6	0	3.374457	-4.193825	-0.279324
15	1	0	1.550640	-5.229990	0.271633
16	6	0	3.937045	-2.962361	-0.625677
17	1	0	3.580938	-0.868531	-0.958181
18	1	0	3.996825	-5.082298	-0.229156
19	1	0	4.997383	-2.887286	-0.845666
20	6	0	-3.440608	-2.946453	0.849120
21	1	0	-4.232860	-2.486671	1.444311
22	1	0	-2.922966	-3.693866	1.455370
23	6	0	-3.928541	1.688909	0.099589
24	6	0	-2.716117	2.322625	-0.267272
25	1	0	-6.009815	1.945085	0.495154
26	6	0	-5.091915	2.450985	0.214862
27	6	0	-2.696920	3.704831	-0.510415
28	6	0	-3.867704	4.452691	-0.389446
29	6	0	-5.064455	3.826817	-0.026492
30	1	0	-1.763829	4.179480	-0.791759
31	1	0	-3.846324	5.521591	-0.578041
32	1	0	-5.975658	4.409849	0.067923
33	6	0	-1.487562	1.531829	-0.388800
34	6	0	-3.997255	0.206944	0.361792
35	8	0	-5.069262	-0.325169	0.652427

36	8	0	-0.399772	2.174740	-0.731969
37	1	0	0.398321	1.521030	-0.779242
38	6	0	-4.078160	-3.651823	-0.368290
39	1	0	-3.316491	-4.144833	-0.979659
40	1	0	-4.784169	-4.415299	-0.026182
41	1	0	-4.625666	-2.938530	-0.991800
42	6	0	4.813349	2.422803	0.715742
43	6	0	4.345786	2.710481	-0.707900
44	1	0	3.526548	3.438132	-0.636797
45	1	0	5.181306	3.178240	-1.235734
46	8	0	3.988460	1.546043	-1.403952
47	1	0	3.106538	1.236728	-1.125891
48	9	0	5.151751	3.579653	1.342718
49	9	0	5.891475	1.609896	0.755580
50	9	0	3.839798	1.832740	1.455761

Rotational constants (GHZ): 0.1484981 0.0897911 0.0587115

- Geometry optimization of **KuQ + 1 TFE** (on carbonyl oxygen B) in the *vacuum* (B3LYP functional 6-31G+(d,p) basis set, BSSE and counterpoise correction)

Energy -1638.688536 Hartree
Stoichiometry C25H17F3O5
Framework group C1[X(C25H17F3O5)]
Deg. of freedom 144
Full point group C1 NOp 1
Largest Abelian subgroup C1 NOp 1
Largest concise Abelian subgroup C1 NOp 1

Standard orientation:

Center Number	Atomic Number	Atomic Type	Coordinates (Angstroms) X	Y	Z
1	6	0	1.044022	1.032203	-0.113174
2	6	0	0.125495	0.019176	-0.455788
3	6	0	2.338511	0.425970	-0.022873
4	6	0	-1.296266	0.314100	-0.625068
5	6	0	0.667016	2.407260	0.079550
6	6	0	2.175608	-0.967357	-0.317331
7	6	0	-1.708887	1.747318	-0.441182
8	8	0	-2.124972	-0.563477	-0.901839
9	6	0	0.819304	-1.229693	-0.591858
10	6	0	-0.764160	2.747000	-0.099376
11	8	0	1.501970	3.300070	0.383555
12	6	0	-3.050900	2.106037	-0.602717
13	6	0	-1.178586	4.072788	0.072222
14	6	0	-3.454572	3.432712	-0.429850
15	1	0	-3.781023	1.347641	-0.860652
16	6	0	-2.520995	4.416021	-0.092755
17	1	0	-0.436815	4.819226	0.334706
18	1	0	-4.500583	3.693839	-0.557702
19	1	0	-2.836909	5.445971	0.042141
20	6	0	0.217116	-2.557174	-0.945043
21	1	0	0.973155	-3.161769	-1.451230
22	1	0	-0.624128	-2.395796	-1.623754
23	6	0	4.654010	-1.248251	-0.001202

24	6	0	4.778609	0.134444	0.282704
25	1	0	5.676798	-3.109590	-0.208117
26	6	0	5.794005	-2.053289	0.009874
27	6	0	6.040275	0.678019	0.570522
28	6	0	7.169435	-0.139954	0.576067
29	6	0	7.046701	-1.504604	0.295452
30	1	0	6.121869	1.737195	0.786928
31	1	0	8.142814	0.285966	0.798971
32	1	0	7.926402	-2.141241	0.299702
33	6	0	3.588901	0.991606	0.272653
34	6	0	3.319415	-1.879701	-0.308617
35	8	0	3.239438	-3.089372	-0.526935
36	8	0	3.759794	2.257166	0.540011
37	1	0	2.849403	2.771522	0.498763
38	6	0	-0.283613	-3.326789	0.297123
39	1	0	-1.054885	-2.760802	0.828153
40	1	0	-0.714320	-4.285848	-0.008580
41	1	0	0.540069	-3.531258	0.987608
42	6	0	-5.826449	-1.531341	0.757939
43	6	0	-5.470268	-1.914470	-0.675658
44	1	0	-4.835295	-2.808289	-0.621083
45	1	0	-6.404961	-2.181619	-1.177575
46	8	0	-4.874109	-0.860011	-1.380109
47	1	0	-3.918656	-0.813762	-1.178068
48	9	0	-6.411060	-2.580735	1.395168
49	9	0	-6.689244	-0.491907	0.819354
50	9	0	-4.731059	-1.182755	1.479104

Rotational constants (GHZ): 0.2225391 0.0657103 0.0530323

- Geometry optimization of **KuQ + 1 TFE** (on carbonyl oxygen C) in the *vacuum* (B3LYP functional 6-31G+(d,p) basis set, BSSE and counterpoise correction)

Energy -1638.688822 Hartree
Stoichiometry C25H17F3O5
Framework group C1[X(C25H17F3O5)]
Deg. of freedom 144
Full point group C1 NOp 1
Largest Abelian subgroup C1 NOp 1
Largest concise Abelian subgroup C1 NOp 1

Standard orientation:

Center Number	Atomic Number	Atomic Type	Coordinates (Angstroms) X	Y	Z
1	6	0	-2.318333	0.427846	-0.005769
2	6	0	-2.161458	-0.964497	0.137077
3	6	0	-1.021921	1.024381	0.126568
4	6	0	-3.312452	-1.871648	0.054324
5	6	0	-3.590418	1.061143	-0.252804
6	6	0	-0.088396	-0.040210	0.352938
7	6	0	-4.644349	-1.222331	-0.216541
8	8	0	-3.227403	-3.090967	0.197832
9	6	0	-0.778328	-1.270870	0.354688
10	6	0	-4.776048	0.179001	-0.359400
11	8	0	-3.708434	2.307590	-0.378260

12	6	0	-5.778464	-2.031375	-0.322766
13	6	0	-6.035669	0.741054	-0.604943
14	6	0	-7.030444	-1.463813	-0.569328
15	1	0	-5.658045	-3.103545	-0.208821
16	6	0	-7.159686	-0.078050	-0.710075
17	1	0	-6.114255	1.817698	-0.710425
18	1	0	-7.905179	-2.102110	-0.651291
19	1	0	-8.133644	0.362145	-0.901055
20	6	0	-0.193709	-2.637031	0.554721
21	1	0	0.829445	-2.642322	0.171621
22	1	0	-0.789407	-3.361220	-0.006004
23	6	0	1.749089	1.670264	0.437495
24	6	0	0.797156	2.698271	0.208619
25	1	0	3.830129	1.232996	0.747419
26	6	0	3.096491	2.012011	0.575898
27	6	0	1.215470	4.034167	0.127047
28	6	0	2.565350	4.356667	0.267165
29	6	0	3.503987	3.346480	0.489762
30	1	0	0.476148	4.807577	-0.047098
31	1	0	2.882342	5.392863	0.201425
32	1	0	4.556027	3.592191	0.596285
33	6	0	-0.622589	2.365038	0.052905
34	6	0	1.331112	0.229117	0.537803
35	8	0	2.159145	-0.663462	0.772802
36	8	0	-1.450087	3.354403	-0.154172
37	1	0	-2.425494	3.002266	-0.256486
38	6	0	-0.173223	-3.057675	2.041078
39	1	0	-1.186170	-3.087473	2.453298
40	1	0	0.260392	-4.058262	2.137642
41	1	0	0.430268	-2.366286	2.636847
42	6	0	5.798512	-1.499353	-0.960622
43	6	0	5.390801	-2.040709	0.406727
44	1	0	4.673187	-2.852204	0.230247
45	1	0	6.290477	-2.464305	0.863454
46	8	0	4.890240	-1.040199	1.250260
47	1	0	3.935452	-0.908482	1.083325
48	9	0	6.286679	-2.501966	-1.738761
49	9	0	6.758416	-0.550175	-0.878552
50	9	0	4.751099	-0.948716	-1.625295

Rotational constants (GHZ): 0.2201106 0.0654044 0.0534537

- Geometry optimization of **KuQ + 1 TFE** (on carbonyl oxygen A) in DCM (B3LYP functional 6-31G+(d,p) basis set)

Energy -1638.699606 Hartree
Stoichiometry C25H17F3O5
Framework group C1[X(C25H17F3O5)]
Deg. of freedom 144
Full point group C1 NOp 1
Largest Abelian subgroup C1 NOp 1
Largest concise Abelian subgroup C1 NOp 1
Standard orientation:

Center Number	Atomic Number	Atomic Type	Coordinates (Angstroms) X	Y	Z

1	6	0	0.486412	0.855381	-0.142126
2	6	0	1.110776	2.078206	0.183877
3	6	0	1.500652	-0.161456	-0.116554
4	6	0	0.348305	3.327932	0.243425
5	6	0	-0.919674	0.743186	-0.421007
6	6	0	2.733853	0.479897	0.228789
7	6	0	-1.124831	3.217023	-0.032712
8	8	0	0.860249	4.422042	0.497565
9	6	0	2.504571	1.857666	0.422051
10	6	0	-1.732525	1.977820	-0.354340
11	8	0	-1.450000	-0.369885	-0.712458
12	6	0	-1.908119	4.371803	0.025222
13	6	0	-3.110488	1.929402	-0.608130
14	6	0	-3.280844	4.312747	-0.228615
15	1	0	-1.427690	5.312410	0.271116
16	6	0	-3.881341	3.091193	-0.545597
17	1	0	-3.579248	0.983951	-0.852671
18	1	0	-3.878907	5.217269	-0.178962
19	1	0	-4.947183	3.040950	-0.743449
20	6	0	3.521915	2.896041	0.791835
21	1	0	4.299202	2.427116	1.399714
22	1	0	3.034241	3.675800	1.381476
23	6	0	3.867352	-1.760090	0.100548
24	6	0	2.630613	-2.365390	-0.236737
25	1	0	5.949680	-2.079846	0.463642
26	6	0	5.009417	-2.555026	0.206488
27	6	0	2.568440	-3.749672	-0.459398
28	6	0	3.719513	-4.530162	-0.347865
29	6	0	4.939275	-3.933792	-0.014528
30	1	0	1.619572	-4.205069	-0.718188
31	1	0	3.664006	-5.600076	-0.520656
32	1	0	5.835185	-4.540356	0.072935
33	6	0	1.423924	-1.543619	-0.349549
34	6	0	3.978937	-0.279449	0.339537
35	8	0	5.072143	0.231874	0.604479
36	8	0	0.313583	-2.156571	-0.666300
37	1	0	-0.466805	-1.482197	-0.712405
38	6	0	4.179816	3.544831	-0.445705
39	1	0	3.433199	4.042596	-1.072031
40	1	0	4.912622	4.293964	-0.128786
41	1	0	4.698354	2.795755	-1.051913
42	6	0	-4.837464	-2.406785	0.652866
43	6	0	-4.128105	-2.743051	-0.653760
44	1	0	-3.207306	-3.280062	-0.397708
45	1	0	-4.782419	-3.417333	-1.212602
46	8	0	-3.894651	-1.596521	-1.435258
47	1	0	-3.074158	-1.155694	-1.142042
48	9	0	-5.068576	-3.533574	1.376693
49	9	0	-6.036058	-1.806096	0.455292
50	9	0	-4.105947	-1.571940	1.435817

Rotational constants (GHZ): 0.1464643 0.0907777 0.0585563

- Geometry optimization of **KuQ + 1 TFE** (on carbonyl oxygen B) in DCM (B3LYP functional 6-31G+(d,p) basis set)

Energy -1638.701895 Hartree

Stoichiometry   C25H17F3O5
Framework group C1[X(C25H17F3O5)]
Deg. of freedom   144
Full point group   C1   NOp   1
Largest Abelian subgroup   C1   NOp   1
Largest concise Abelian subgroup C1   NOp   1

Standard orientation:

Center Number	Atomic Number	Atomic Type	Coordinates (Angstroms) X	Y	Z
1	6	0	1.078557	1.052246	-0.082152
2	6	0	0.122776	0.055583	-0.373770
3	6	0	2.357109	0.414809	-0.014913
4	6	0	-1.291630	0.385549	-0.515468
5	6	0	0.740522	2.441511	0.087107
6	6	0	2.151685	-0.980166	-0.269569
7	6	0	-1.663406	1.829704	-0.348987
8	8	0	-2.152250	-0.475192	-0.758938
9	6	0	0.780123	-1.212703	-0.498209
10	6	0	-0.685554	2.813702	-0.058005
11	8	0	1.607304	3.319186	0.345508
12	6	0	-3.000558	2.217695	-0.477148
13	6	0	-1.065306	4.152250	0.095044
14	6	0	-3.370383	3.556847	-0.324133
15	1	0	-3.753482	1.469925	-0.696103
16	6	0	-2.404031	4.524722	-0.038125
17	1	0	-0.303453	4.890829	0.318097
18	1	0	-4.412783	3.840996	-0.427805
19	1	0	-2.690808	5.564614	0.081307
20	6	0	0.131505	-2.531235	-0.799451
21	1	0	0.851707	-3.166774	-1.319400
22	1	0	-0.727169	-2.363572	-1.453921
23	6	0	4.629681	-1.319148	-0.021561
24	6	0	4.798397	0.067349	0.224740
25	1	0	5.609026	-3.209842	-0.212179
26	6	0	5.750207	-2.151163	-0.024344
27	6	0	6.081545	0.584427	0.461150
28	6	0	7.190732	-0.261476	0.453398
29	6	0	7.025437	-1.628415	0.210314
30	1	0	6.200376	1.645229	0.649258
31	1	0	8.180132	0.144964	0.636495
32	1	0	7.887882	-2.287509	0.203783
33	6	0	3.632829	0.953772	0.229506
34	6	0	3.271876	-1.918860	-0.272670
35	8	0	3.150838	-3.134377	-0.460820
36	8	0	3.839987	2.220282	0.460200
37	1	0	2.940798	2.753518	0.432634
38	6	0	-0.346720	-3.259770	0.475833
39	1	0	-1.084545	-2.661076	1.018598
40	1	0	-0.811718	-4.214228	0.208293
41	1	0	0.492737	-3.466441	1.146740
42	6	0	-5.912438	-1.567613	0.624688
43	6	0	-5.333549	-1.979430	-0.724045
44	1	0	-4.586910	-2.759227	-0.534179
45	1	0	-6.151811	-2.413315	-1.305628
46	8	0	-4.810724	-0.880734	-1.427548
47	1	0	-3.884247	-0.719145	-1.151956
48	9	0	-6.429411	-2.644326	1.274633
49	9	0	-6.910171	-0.657018	0.507437

50	9	0	-4.977464	-1.019032	1.442749

Rotational constants (GHZ): 0.2197195 0.0654244 0.0522959

- Geometry optimization of **KuQ + 1 TFE** (on carbonyl oxygen C) in DCM (B3LYP functional 6-31G+(d,p) basis set)

Energy -1638.702141 Hartree
Stoichiometry C25H17F3O5
Framework group C1[X(C25H17F3O5)]
Deg. of freedom 144
Full point group C1 NOp 1
Largest Abelian subgroup C1 NOp 1
Largest concise Abelian subgroup C1 NOp 1

Standard orientation:

Center Number	Atomic Number	Atomic Type	Coordinates (Angstroms) X	Y	Z
1	6	0	-2.343216	0.414413	-0.014711
2	6	0	-2.134418	-0.974223	0.110966
3	6	0	-1.068276	1.055198	0.095973
4	6	0	-3.253183	-1.918609	0.042201
5	6	0	-3.643841	1.002282	-0.227078
6	6	0	-0.091779	0.024434	0.292470
7	6	0	-4.612225	-1.318161	-0.191980
8	8	0	-3.119065	-3.139189	0.170536
9	6	0	-0.737427	-1.230963	0.296853
10	6	0	-4.797867	0.079943	-0.316771
11	8	0	-3.807381	2.246290	-0.336584
12	6	0	-5.719991	-2.165106	-0.282563
13	6	0	-6.082628	0.597314	-0.528197
14	6	0	-6.997849	-1.641468	-0.495275
15	1	0	-5.566525	-3.234202	-0.184531
16	6	0	-7.179793	-0.260189	-0.617658
17	1	0	-6.207760	1.670440	-0.620765
18	1	0	-7.849979	-2.310383	-0.564887
19	1	0	-8.172342	0.147007	-0.782040
20	6	0	-0.096326	-2.575208	0.472057
21	1	0	0.917568	-2.538941	0.067022
22	1	0	-0.672744	-3.316811	-0.085691
23	6	0	1.682943	1.797941	0.352475
24	6	0	0.691278	2.794601	0.154250
25	1	0	3.781416	1.429586	0.614144
26	6	0	3.019945	2.185683	0.464536
27	6	0	1.062225	4.144638	0.076971
28	6	0	2.403362	4.513167	0.191339
29	6	0	3.381075	3.534201	0.383609
30	1	0	0.296785	4.897105	-0.073040
31	1	0	2.682263	5.560060	0.129940
32	1	0	4.425405	3.816382	0.471241
33	6	0	-0.717545	2.412018	0.026716
34	6	0	1.318469	0.344906	0.449509
35	8	0	2.185560	-0.520409	0.659708
36	8	0	-1.586479	3.369230	-0.152780
37	1	0	-2.549983	2.978159	-0.236283
38	6	0	-0.027838	-3.008367	1.953260
39	1	0	-1.029442	-3.077310	2.388369
40	1	0	0.446126	-3.992121	2.032383

41	1	0	0.559286	-2.297991	2.543210
42	6	0	5.898921	-1.584088	-0.842723
43	6	0	5.348297	-2.021003	0.510055
44	1	0	4.589840	-2.789508	0.320776
45	1	0	6.176155	-2.475763	1.061432
46	8	0	4.852838	-0.933759	1.249874
47	1	0	3.918108	-0.764512	1.006708
48	9	0	6.391473	-2.650129	-1.528281
49	9	0	6.907049	-0.684394	-0.728743
50	9	0	4.950163	-1.009767	-1.626499

Rotational constants (GHZ): 0.2148808 0.0651477 0.0525329

- Geometry optimization of **KuQ + 2 TFE** (on carbonyl oxygens B-C) in the *vacuum* (B3LYP functional 6-31G+(d,p) basis set, BSSE and counterpoise correction)

Energy -2091.496547 Hartree
Stoichiometry C27H20F6O6
Framework group C1[X(C27H20F6O6)]
Deg. of freedom 171
Full point group C1 NOp 1
Largest Abelian subgroup C1 NOp 1
Largest concise Abelian subgroup C1 NOp 1
Standard orientation:

Center Number	Atomic Number	Atomic Type	Coordinates (Angstroms) X	Y	Z
1	6	0	0.697928	-1.917110	-0.152079
2	6	0	1.101151	-0.573757	-0.291342
3	6	0	-0.697617	-1.918572	0.162030
4	6	0	2.486221	-0.228988	-0.610262
5	6	0	1.594159	-3.032627	-0.325551
6	6	0	-1.123433	-0.550920	0.208993
7	6	0	3.432280	-1.378889	-0.810866
8	8	0	2.869136	0.944247	-0.707516
9	6	0	-0.025262	0.287586	-0.075847
10	6	0	3.000563	-2.721638	-0.669074
11	8	0	1.214621	-4.225746	-0.198767
12	6	0	4.768136	-1.124820	-1.137370
13	6	0	3.906609	-3.772359	-0.855013
14	6	0	5.664586	-2.180942	-1.321801
15	1	0	5.108764	-0.101472	-1.244521
16	6	0	5.236263	-3.503433	-1.181117
17	1	0	3.552311	-4.790928	-0.739848
18	1	0	6.698457	-1.965934	-1.573779
19	1	0	5.934879	-4.322118	-1.323836
20	6	0	-0.026968	1.785843	-0.133232
21	1	0	-1.014879	2.124243	-0.453776
22	1	0	0.715386	2.112712	-0.865126
23	6	0	-3.444641	-1.355901	0.734993
24	6	0	-2.988544	-2.699394	0.676661
25	1	0	-5.148641	-0.086127	1.056993
26	6	0	-4.791418	-1.108392	1.011149
27	6	0	-3.886324	-3.754401	0.895453
28	6	0	-5.228004	-3.488342	1.168242

29	6	0	-5.678908	-2.167457	1.224723
30	1	0	-3.522053	-4.774309	0.848484
31	1	0	-5.918046	-4.309526	1.335271
32	1	0	-6.722711	-1.955454	1.434588
33	6	0	-1.581076	-2.986111	0.385664
34	6	0	-2.505679	-0.202412	0.512008
35	8	0	-2.900260	0.969690	0.593069
36	8	0	-1.219027	-4.238535	0.345308
37	1	0	-0.201219	-4.323235	0.124525
38	6	0	0.301883	2.428168	1.232849
39	1	0	1.293800	2.125671	1.581458
40	1	0	0.291330	3.519231	1.142579
41	1	0	-0.437035	2.143459	1.987724
42	6	0	-6.165807	2.916033	-1.109729
43	6	0	-5.528370	3.426830	0.179267
44	1	0	-4.630515	3.989797	-0.107111
45	1	0	-6.241287	4.118566	0.637589
46	8	0	-5.262285	2.396006	1.090671
47	1	0	-4.418095	1.954827	0.869657
48	9	0	-6.415443	3.950926	-1.955093
49	9	0	-7.339790	2.280813	-0.892623
50	9	0	-5.357986	2.045640	-1.766738
51	6	0	6.135900	2.926339	0.958871
52	6	0	5.490449	3.431069	-0.328509
53	1	0	4.594383	3.995395	-0.039076
54	1	0	6.200285	4.120418	-0.794944
55	8	0	5.218793	2.396476	-1.234070
56	1	0	4.383453	1.947382	-0.998066
57	9	0	6.389106	3.965160	1.798368
58	9	0	7.308844	2.291074	0.738312
59	9	0	5.331841	2.058375	1.624106

Rotational constants (GHZ): 0.1144218 0.0437141 0.0339828

- Geometry optimization of **KuQ + 2 TFE** (on carbonyl oxygens B-C) in DCM (B3LYP functional 6-31G+(d,p) basis set)

Energy -2091.513639 Hartree
Stoichiometry C27H20F6O6
Framework group C1[X(C27H20F6O6)]
Deg. of freedom 171
Full point group C1 NOp 1
Largest Abelian subgroup C1 NOp 1
Largest concise Abelian subgroup C1 NOp 1

Standard orientation:

Center Number	Atomic Number	Atomic Type	Coordinates (Angstroms) X	Y	Z
1	6	0	0.687709	1.947924	0.156356
2	6	0	1.094832	0.603826	0.291498
3	6	0	-0.703331	1.946721	-0.169531
4	6	0	2.475653	0.263114	0.620636
5	6	0	1.580966	3.064371	0.344847
6	6	0	-1.125693	0.578662	-0.227651
7	6	0	3.416092	1.411788	0.836784
8	8	0	2.863268	-0.911872	0.715304

9	6	0	-0.026625	-0.258613	0.061409
10	6	0	2.984135	2.755072	0.700013
11	8	0	1.198645	4.257694	0.221062
12	6	0	4.748452	1.157960	1.175918
13	6	0	3.888400	3.804253	0.903316
14	6	0	5.643714	2.211857	1.379154
15	1	0	5.085506	0.133496	1.279803
16	6	0	5.215243	3.534862	1.242703
17	1	0	3.540163	4.825157	0.793191
18	1	0	6.674480	1.997291	1.642823
19	1	0	5.910580	4.353208	1.399589
20	6	0	-0.024046	-1.757281	0.108974
21	1	0	-1.013381	-2.102225	0.417261
22	1	0	0.709875	-2.087107	0.847828
23	6	0	-3.442076	1.379402	-0.770024
24	6	0	-2.992080	2.724669	-0.703503
25	1	0	-5.133719	0.104222	-1.114272
26	6	0	-4.784207	1.128416	-1.062643
27	6	0	-3.892152	3.775897	-0.930629
28	6	0	-5.229895	3.505485	-1.220959
29	6	0	-5.675097	2.182985	-1.286019
30	1	0	-3.537249	4.798586	-0.878268
31	1	0	-5.920424	4.324157	-1.395267
32	1	0	-6.715050	1.968594	-1.510571
33	6	0	-1.589754	3.013679	-0.396303
34	6	0	-2.503494	0.230352	-0.540752
35	8	0	-2.897466	-0.944947	-0.624505
36	8	0	-1.229252	4.265359	-0.345053
37	1	0	-0.213839	4.347883	-0.114186
38	6	0	0.322608	-2.390696	-1.256804
39	1	0	1.317063	-2.081545	-1.592459
40	1	0	0.314184	-3.482311	-1.173784
41	1	0	-0.406317	-2.101261	-2.019809
42	6	0	-6.039721	-2.959139	1.153655
43	6	0	-5.213087	-3.508784	-0.003468
44	1	0	-4.245175	-3.820396	0.406440
45	1	0	-5.734720	-4.393720	-0.378878
46	8	0	-5.090559	-2.577776	-1.049063
47	1	0	-4.335804	-1.977428	-0.873446
48	9	0	-6.160807	-3.886121	2.141011
49	9	0	-7.295343	-2.611461	0.778117
50	9	0	-5.479008	-1.854538	1.710569
51	6	0	6.036021	-2.955332	-0.986937
52	6	0	5.191912	-3.494951	0.162212
53	1	0	4.232408	-3.815204	-0.260464
54	1	0	5.710424	-4.373197	0.556996
55	8	0	5.047981	-2.552893	1.195287
56	1	0	4.299697	-1.952407	0.996660
57	9	0	6.176413	-3.892990	-1.961549
58	9	0	7.283977	-2.599055	-0.594658
59	9	0	5.480710	-1.859091	-1.565373

Rotational constants (GHZ): 0.1115989 0.0446943 0.0344311

- Geometry optimization of **KuQ**$^{\cdot -}$ in the *vacuum* (B3LYP functional 6-31G+(d,p) basis set)

Energy -1185.977692 Hartree
Stoichiometry C23H14O4(1-,2)
Framework group C1[X(C23H14O4)]
Deg. of freedom 117
Full point group C1 NOp 1
Largest Abelian subgroup C1 NOp 1
Largest concise Abelian subgroup C1 NOp 1

Standard orientation:

Center Number	Atomic Number	Atomic Type	Coordinates (Angstroms) X	Y	Z
1	6	0	0.722295	-0.403013	-0.017784
2	6	0	1.147145	0.962965	-0.125749
3	6	0	-0.720341	-0.401315	-0.018510
4	6	0	2.548809	1.326513	-0.154590
5	6	0	1.617015	-1.496908	0.058028
6	6	0	-1.146624	0.972638	-0.127108
7	6	0	3.509107	0.172979	-0.087638
8	8	0	2.981690	2.494370	-0.224722
9	6	0	0.000369	1.802595	-0.199154
10	6	0	3.062707	-1.168735	0.017213
11	8	0	1.251801	-2.720609	0.153793
12	6	0	4.886130	0.437008	-0.124505
13	6	0	4.009455	-2.205939	0.081858
14	6	0	5.814881	-0.599247	-0.061390
15	1	0	5.197087	1.474156	-0.203065
16	6	0	5.372791	-1.927287	0.042786
17	1	0	3.641898	-3.223743	0.162357
18	1	0	6.879251	-0.379041	-0.092169
19	1	0	6.094120	-2.739495	0.093194
20	6	0	0.004249	3.299120	-0.324995
21	1	0	-0.894533	3.608254	-0.865360
22	1	0	0.894616	3.603629	-0.882067
23	6	0	-3.503046	0.184758	-0.088206
24	6	0	-3.051498	-1.162216	0.017004
25	1	0	-5.191907	1.480845	-0.201841
26	6	0	-4.881143	0.443589	-0.123232
27	6	0	-4.004864	-2.202190	0.082941
28	6	0	-5.365007	-1.921141	0.045225
29	6	0	-5.809797	-0.591416	-0.058879
30	1	0	-3.649107	-3.223597	0.163323
31	1	0	-6.085663	-2.733851	0.096671
32	1	0	-6.874268	-0.372357	-0.088408
33	6	0	-1.630158	-1.457696	0.054016
34	6	0	-2.548609	1.335451	-0.156513
35	8	0	-2.979343	2.506772	-0.228401
36	8	0	-1.273751	-2.740920	0.153304
37	1	0	-0.240622	-2.812499	0.162572
38	6	0	0.019130	4.005806	1.046918
39	1	0	0.914289	3.731200	1.614087
40	1	0	0.021576	5.094646	0.913017
41	1	0	-0.867879	3.737042	1.629490

Rotational constants (GHZ): 0.3799631 0.1309389 0.0984049

- Geometry optimization of **KuQ**$^{\cdot-}$ in DCM (B3LYP functional 6-31G+(d,p) basis set)

Energy -1186.037045 Hartree
Stoichiometry C23H14O4(1-,2)
Framework group C1[X(C23H14O4)]
Deg. of freedom 117
Full point group C1 NOp 1
Largest Abelian subgroup C1 NOp 1
Largest concise Abelian subgroup C1 NOp 1
Standard orientation:

Center Number	Atomic Number	Atomic Type	Coordinates (Angstroms) X	Y	Z
1	6	0	0.721646	-0.402890	-0.016737
2	6	0	1.147512	0.963266	-0.125093
3	6	0	-0.721040	-0.400490	-0.017374
4	6	0	2.549869	1.321596	-0.157554
5	6	0	1.618936	-1.493943	0.059190
6	6	0	-1.147500	0.973539	-0.126205
7	6	0	3.510199	0.172660	-0.089880
8	8	0	2.978628	2.494679	-0.232622
9	6	0	0.001045	1.804019	-0.196978
10	6	0	3.063013	-1.170305	0.017241
11	8	0	1.245577	-2.719078	0.156656
12	6	0	4.888382	0.433711	-0.127881
13	6	0	4.010065	-2.207340	0.082707
14	6	0	5.816759	-0.603319	-0.063379
15	1	0	5.212219	1.466005	-0.208478
16	6	0	5.374277	-1.930207	0.042833
17	1	0	3.652413	-3.228058	0.164867
18	1	0	6.880088	-0.383601	-0.094948
19	1	0	6.094110	-2.742195	0.094176
20	6	0	0.006392	3.300595	-0.317862
21	1	0	-0.891196	3.613398	-0.856980
22	1	0	0.891523	3.606141	-0.881716
23	6	0	-3.505513	0.186817	-0.089796
24	6	0	-3.054951	-1.160966	0.017204
25	1	0	-5.209156	1.475478	-0.205905
26	6	0	-4.885096	0.443199	-0.125720
27	6	0	-4.007228	-2.201301	0.083738
28	6	0	-5.368295	-1.921662	0.045371
29	6	0	-5.812789	-0.592888	-0.060237
30	1	0	-3.658396	-3.224712	0.165321
31	1	0	-6.087935	-2.733751	0.097410
32	1	0	-6.876281	-0.374265	-0.090421
33	6	0	-1.633487	-1.454193	0.055175
34	6	0	-2.549314	1.332205	-0.158893
35	8	0	-2.976593	2.509115	-0.235665
36	8	0	-1.271253	-2.737227	0.155379
37	1	0	-0.236276	-2.801148	0.164796
38	6	0	0.029286	4.005749	1.055080
39	1	0	0.924540	3.729586	1.621095
40	1	0	0.031504	5.093739	0.923778
41	1	0	-0.851058	3.736369	1.647340

Rotational constants (GHZ): 0.3799331 0.1308277
0.0983561

- Geometry optimization of **KuQ·⁻ + 1 TFE** (on carbonyl oxygen A) in the *vacuum* (B3LYP functional 6-31G+(d,p) basis set, BSSE and counterpoise correction)

Energy -1638.794009 Hartree
Stoichiometry C25H17F3O5(1-,2)
Framework group C1[X(C25H17F3O5)]
Deg. of freedom 144
Full point group C1 NOp 1
Largest Abelian subgroup C1 NOp 1
Largest concise Abelian subgroup C1 NOp 1

Standard orientation:

Center Number	Atomic Number	Atomic Type	Coordinates (Angstroms) X	Y	Z
1	6	0	0.560720	0.842073	-0.072343
2	6	0	1.305776	2.044815	0.164761
3	6	0	1.510969	-0.251995	-0.056475
4	6	0	0.664895	3.343347	0.211531
5	6	0	-0.836429	0.824076	-0.264542
6	6	0	2.811932	0.317273	0.191702
7	6	0	-0.823681	3.327003	0.030771
8	8	0	1.263527	4.423071	0.384201
9	6	0	2.680037	1.721434	0.332376
10	6	0	-1.540769	2.122477	-0.197126
11	8	0	-1.501352	-0.264321	-0.486293
12	6	0	-1.517492	4.543657	0.088694
13	6	0	-2.938252	2.182268	-0.353693
14	6	0	-2.900247	4.587084	-0.069507
15	1	0	-0.936804	5.444264	0.261580
16	6	0	-3.611515	3.398991	-0.290539
17	1	0	-3.492531	1.266382	-0.520783
18	1	0	-3.425257	5.537716	-0.020422
19	1	0	-4.691233	3.422083	-0.412499
20	6	0	3.792145	2.692698	0.604567
21	1	0	4.566974	2.185340	1.185249
22	1	0	3.392246	3.534711	1.175762
23	6	0	3.777040	-1.973163	0.106855
24	6	0	2.481078	-2.507478	-0.136034
25	1	0	5.848728	-2.403962	0.370115
26	6	0	4.873465	-2.843534	0.185721
27	6	0	2.331081	-3.902227	-0.292117
28	6	0	3.432048	-4.746346	-0.208665
29	6	0	4.711482	-4.217668	0.032001
30	1	0	1.339285	-4.299173	-0.478397
31	1	0	3.300031	-5.818546	-0.330649
32	1	0	5.571734	-4.878924	0.097232
33	6	0	1.329970	-1.624386	-0.220246
34	6	0	4.003772	-0.503591	0.274091
35	8	0	5.156356	-0.062968	0.464388
36	8	0	0.143477	-2.195741	-0.451007
37	1	0	-0.573535	-1.463969	-0.488275
38	6	0	4.427698	3.237307	-0.692208
39	1	0	3.683357	3.765991	-1.295991
40	1	0	5.233233	3.941562	-0.451551
41	1	0	4.854674	2.422669	-1.285728

42	6	0	-5.215572	-2.226181	0.422810
43	6	0	-4.037821	-2.380929	-0.532906
44	1	0	-3.157480	-2.607678	0.080761
45	1	0	-4.260562	-3.256909	-1.156391
46	8	0	-3.867653	-1.245722	-1.329646
47	1	0	-3.043348	-0.779554	-1.048554
48	9	0	-5.379437	-3.367291	1.157580
49	9	0	-6.387566	-2.009808	-0.224658
50	9	0	-5.055034	-1.210522	1.301359

Rotational constants (GHZ): 0.1451098 0.0878692 0.0563877

- Geometry optimization of **KuQ**$^{\cdot-}$ **+ 1 TFE** (on carbonyl oxygen B) in the *vacuum* (B3LYP functional 6-31G+(d,p) basis set, BSSE and counterpoise correction)

Energy -1638.796092 Hartree
Stoichiometry C25H17F3O5(1-,2)
Framework group C1[X(C25H17F3O5)]
Deg. of freedom 144
Full point group C1 NOp 1
Largest Abelian subgroup C1 NOp 1
Largest concise Abelian subgroup C1 NOp 1

Standard orientation:

Center Number	Atomic Number	Atomic Type	Coordinates (Angstroms) X	Y	Z
1	6	0	1.156401	1.110759	-0.046376
2	6	0	0.130263	0.138582	-0.282656
3	6	0	2.411996	0.406537	-0.006008
4	6	0	-1.255209	0.509473	-0.385730
5	6	0	0.898850	2.495824	0.094541
6	6	0	2.125447	-0.992786	-0.218882
7	6	0	-1.551939	1.965586	-0.242498
8	8	0	-2.182869	-0.323713	-0.578881
9	6	0	0.733638	-1.153632	-0.395737
10	6	0	-0.517099	2.912574	-0.012197
11	8	0	1.806159	3.375192	0.301789
12	6	0	-2.879856	2.417297	-0.326781
13	6	0	-0.842330	4.273402	0.119669
14	6	0	-3.186720	3.768779	-0.194541
15	1	0	-3.670463	1.694463	-0.493225
16	6	0	-2.162817	4.701637	0.028825
17	1	0	-0.031325	4.972532	0.294793
18	1	0	-4.220660	4.096245	-0.263200
19	1	0	-2.399938	5.757512	0.132957
20	6	0	0.020144	-2.449373	-0.651860
21	1	0	0.703007	-3.129295	-1.167529
22	1	0	-0.850603	-2.253894	-1.283701
23	6	0	4.564556	-1.456392	-0.044759
24	6	0	4.813504	-0.070175	0.159766
25	1	0	5.419695	-3.401795	-0.218578
26	6	0	5.643471	-2.351112	-0.062370
27	6	0	6.142321	0.369279	0.339681
28	6	0	7.197061	-0.535947	0.317117
29	6	0	6.950372	-1.904032	0.114798

30	1	0	6.319838	1.427732	0.495330
31	1	0	8.215241	-0.181246	0.456939
32	1	0	7.775810	-2.611042	0.097187
33	6	0	3.711119	0.879210	0.180987
34	6	0	3.177999	-1.991383	-0.236065
35	8	0	2.992823	-3.215066	-0.389976
36	8	0	4.013870	2.162138	0.376823
37	1	0	3.144903	2.730320	0.361727
38	6	0	-0.455669	-3.126468	0.651005
39	1	0	-1.147427	-2.477538	1.197583
40	1	0	-0.974127	-4.065797	0.423255
41	1	0	0.395878	-3.358496	1.298358
42	6	0	-6.105160	-1.643152	0.440995
43	6	0	-5.054383	-1.859721	-0.642058
44	1	0	-4.188187	-2.325969	-0.157019
45	1	0	-5.488193	-2.580198	-1.348981
46	8	0	-4.729053	-0.667231	-1.291280
47	1	0	-3.801216	-0.423173	-1.045772
48	9	0	-6.431022	-2.835291	1.027059
49	9	0	-7.259423	-1.123900	-0.047536
50	9	0	-5.687936	-0.819642	1.429241

Rotational constants (GHZ): 0.2122606 0.0644787 0.0508167

- Geometry optimization of **KuQ·⁻ + 1 TFE** (on carbonyl oxygen C) in the *vacuum* (B3LYP functional 6-31G+(d,p) basis set, BSSE and counterpoise correction)

Energy -1638.796726 Hartree
Stoichiometry C25H17F3O5(1-,2)
Framework group C1[X(C25H17F3O5)]
Deg. of freedom 144
Full point group C1 NOp 1
Largest Abelian subgroup C1 NOp 1
Largest concise Abelian subgroup C1 NOp 1

Standard orientation:

Center Number	Atomic Number	Atomic Type	Coordinates (Angstroms) X	Y	Z
1	6	0	-2.414071	0.400216	-0.028889
2	6	0	-2.101803	-0.998879	0.042154
3	6	0	-1.167620	1.117860	0.037879
4	6	0	-3.138116	-2.014520	0.002550
5	6	0	-3.732732	0.902209	-0.153645
6	6	0	-0.114342	0.139790	0.149505
7	6	0	-4.540932	-1.493599	-0.144938
8	8	0	-2.935321	-3.239577	0.086014
9	6	0	-0.696498	-1.159659	0.144423
10	6	0	-4.820658	-0.106442	-0.214713
11	8	0	-4.024118	2.144568	-0.218165
12	6	0	-5.598387	-2.411792	-0.207983
13	6	0	-6.152328	0.321816	-0.345673
14	6	0	-6.915180	-1.975836	-0.339921
15	1	0	-5.353412	-3.467691	-0.149960
16	6	0	-7.192017	-0.602402	-0.408417
17	1	0	-6.340435	1.389222	-0.396147

18	1	0	-7.725380	-2.699056	-0.388892
19	1	0	-8.218156	-0.257810	-0.510386
20	6	0	0.039510	-2.465233	0.233775
21	1	0	1.015669	-2.351878	-0.245488
22	1	0	-0.538467	-3.231403	-0.289347
23	6	0	1.538738	1.985718	0.185816
24	6	0	0.476827	2.933515	0.073899
25	1	0	3.668869	1.734382	0.316283
26	6	0	2.862687	2.455047	0.239161
27	6	0	0.783730	4.310971	0.027819
28	6	0	2.100329	4.747398	0.085434
29	6	0	3.147498	3.815526	0.189489
30	1	0	-0.034508	5.017707	-0.055345
31	1	0	2.319490	5.811536	0.047869
32	1	0	4.179326	4.153547	0.230860
33	6	0	-0.901243	2.488527	0.002922
34	6	0	1.267990	0.525257	0.246621
35	8	0	2.209544	-0.308354	0.379294
36	8	0	-1.845946	3.425432	-0.098437
37	1	0	-2.774416	2.975794	-0.149925
38	6	0	0.255463	-2.922133	1.692111
39	1	0	-0.704542	-3.063215	2.198739
40	1	0	0.794449	-3.877142	1.714854
41	1	0	0.843984	-2.184712	2.246913
42	6	0	6.125117	-1.689944	-0.575292
43	6	0	5.078168	-1.832132	0.523688
44	1	0	4.211150	-2.331566	0.074349
45	1	0	5.515137	-2.503018	1.276294
46	8	0	4.753433	-0.598644	1.089879
47	1	0	3.819894	-0.376541	0.838491
48	9	0	6.450187	-2.919798	-1.078996
49	9	0	7.280847	-1.137854	-0.127818
50	9	0	5.704503	-0.937151	-1.617017

Rotational constants (GHZ): 0.2071279 0.0643277 0.0506751

- Geometry optimization of **KuQ$^{\cdot-}$ + 1 TFE** (on carbonyl oxygen A) in DCM (B3LYP functional 6-31G+(d,p) basis set)

Energy -1638.851358 Hartree
Stoichiometry C25H17F3O5(1-,2)
Framework group C1[X(C25H17F3O5)]
Deg. of freedom 144
Full point group C1 NOp 1
Largest Abelian subgroup C1 NOp 1
Largest concise Abelian subgroup C1 NOp 1

Standard orientation:

Center Number	Atomic Number	Atomic Type	Coordinates (Angstroms) X	Y	Z
1	6	0	0.498258	0.823033	-0.084441
2	6	0	1.165089	2.070136	0.160596
3	6	0	1.512928	-0.209305	-0.065804
4	6	0	0.441233	3.322769	0.207040
5	6	0	-0.894881	0.720196	-0.284547
6	6	0	2.775773	0.438075	0.190236

7	6	0	-1.039516	3.217438	0.017642
8	8	0	0.974797	4.439698	0.387051
9	6	0	2.556697	1.831693	0.335715
10	6	0	-1.678816	1.970542	-0.220008
11	8	0	-1.486641	-0.409324	-0.510145
12	6	0	-1.811391	4.387190	0.074476
13	6	0	-3.075814	1.943858	-0.388526
14	6	0	-3.193238	4.344492	-0.094990
15	1	0	-1.300139	5.326880	0.254896
16	6	0	-3.826228	3.115256	-0.327160
17	1	0	-3.570399	0.996843	-0.566677
18	1	0	-3.776701	5.259314	-0.046834
19	1	0	-4.903440	3.072327	-0.459360
20	6	0	3.605009	2.867620	0.622033
21	1	0	4.394006	2.411597	1.225496
22	1	0	3.148180	3.685855	1.184053
23	6	0	3.881682	-1.788267	0.109220
24	6	0	2.621907	-2.402208	-0.140464
25	1	0	5.978504	-2.105359	0.387998
26	6	0	5.027919	-2.592474	0.197613
27	6	0	2.557699	-3.803418	-0.294045
28	6	0	3.707230	-4.580302	-0.201301
29	6	0	4.949944	-3.974601	0.046500
30	1	0	1.595862	-4.265619	-0.485415
31	1	0	3.641243	-5.657857	-0.321096
32	1	0	5.847481	-4.581735	0.119643
33	6	0	1.419715	-1.590754	-0.232087
34	6	0	4.012875	-0.309824	0.273308
35	8	0	5.137798	0.208014	0.463178
36	8	0	0.270581	-2.230926	-0.470315
37	1	0	-0.492656	-1.544795	-0.504427
38	6	0	4.237751	3.441850	-0.663477
39	1	0	3.480651	3.922254	-1.291329
40	1	0	4.995894	4.191614	-0.410906
41	1	0	4.721733	2.651435	-1.245498
42	6	0	-5.018635	-2.314499	0.473100
43	6	0	-4.062417	-2.615449	-0.675272
44	1	0	-3.160708	-3.059518	-0.237473
45	1	0	-4.549509	-3.366933	-1.304569
46	8	0	-3.792017	-1.472308	-1.443100
47	1	0	-2.989354	-1.017995	-1.090136
48	9	0	-5.287351	-3.443985	1.184062
49	9	0	-6.209660	-1.825882	0.043673
50	9	0	-4.516483	-1.405445	1.346282

Rotational constants (GHZ): 0.1492353 0.0887870 0.0575433

- Geometry optimization of **KuQ$^{\cdot -}$ + 1 TFE** (on carbonyl oxygen B) in DCM (B3LYP functional 6-31G+(d,p) basis set)

```
Energy -1638.852521 Hartree
Stoichiometry    C25H17F3O5(1-,2)
 Framework group  C1[X(C25H17F3O5)]
 Deg. of freedom   144
 Full point group                C1      NOp   1
 Largest Abelian subgroup        C1      NOp   1
 Largest concise Abelian subgroup C1     NOp   1
```

Standard orientation:

Center Number	Atomic Number	Atomic Type	Coordinates (Angstroms) X	Y	Z
1	6	0	1.048099	1.063959	-0.094656
2	6	0	0.106326	0.032313	-0.418078
3	6	0	2.347225	0.445889	-0.012426
4	6	0	-1.295434	0.313791	-0.582582
5	6	0	0.683013	2.419823	0.078534
6	6	0	2.171908	-0.960090	-0.290321
7	6	0	-1.700029	1.739469	-0.416609
8	8	0	-2.149416	-0.576232	-0.849997
9	6	0	0.802276	-1.209960	-0.542682
10	6	0	-0.747686	2.746347	-0.097273
11	8	0	1.519845	3.350503	0.368683
12	6	0	-3.047845	2.104887	-0.573654
13	6	0	-1.174867	4.077216	0.051541
14	6	0	-3.455461	3.428223	-0.424342
15	1	0	-3.775972	1.338998	-0.813758
16	6	0	-2.514289	4.419452	-0.109962
17	1	0	-0.433299	4.830312	0.294668
18	1	0	-4.502054	3.689133	-0.550991
19	1	0	-2.828659	5.452498	0.007690
20	6	0	0.191285	-2.540661	-0.873275
21	1	0	0.934506	-3.149054	-1.393781
22	1	0	-0.668563	-2.382941	-1.528955
23	6	0	4.626617	-1.261536	-0.007664
24	6	0	4.766955	0.129302	0.264324
25	1	0	5.639863	-3.134425	-0.207452
26	6	0	5.767844	-2.077257	0.000509
27	6	0	6.049616	0.651143	0.534411
28	6	0	7.166721	-0.177257	0.535947
29	6	0	7.028440	-1.548645	0.267694
30	1	0	6.150735	1.710656	0.741035
31	1	0	8.147408	0.240110	0.745543
32	1	0	7.900938	-2.195349	0.268845
33	6	0	3.599515	0.996440	0.260478
34	6	0	3.294686	-1.876694	-0.295697
35	8	0	3.201709	-3.105074	-0.518081
36	8	0	3.793882	2.291113	0.519117
37	1	0	2.883829	2.791933	0.476305
38	6	0	-0.279265	-3.304419	0.383447
39	1	0	-1.039663	-2.734555	0.926593
40	1	0	-0.714144	-4.269295	0.099514
41	1	0	0.559264	-3.495928	1.060553
42	6	0	-5.844236	-1.503091	0.708377
43	6	0	-5.133501	-2.082475	-0.509569
44	1	0	-4.303828	-2.695467	-0.137884
45	1	0	-5.852398	-2.737333	-1.012746
46	8	0	-4.714394	-1.079327	-1.395099
47	1	0	-3.784569	-0.816720	-1.177764
48	9	0	-6.264901	-2.497092	1.538978
49	9	0	-6.942128	-0.777742	0.374819
50	9	0	-5.045622	-0.685992	1.440287

Rotational constants (GHZ): 0.2232545 0.0658309
0.0529853

- Geometry optimization of **KuQ$^{\cdot-}$ + 1 TFE** (on carbonyl oxygen C) in DCM (B3LYP functional 6-31G+(d,p) basis set)

```
Energy -1638.853069 Hartree
Stoichiometry    C25H17F3O5(1-,2)
 Framework group  C1[X(C25H17F3O5)]
 Deg. of freedom   144
 Full point group                C1      NOp   1
 Largest Abelian subgroup        C1      NOp   1
 Largest concise Abelian subgroup C1      NOp   1
                         Standard orientation:
```

Center Number	Atomic Number	Atomic Type	Coordinates (Angstroms) X	Y	Z
1	6	0	-2.335127	0.441716	-0.014865
2	6	0	-2.107159	-0.966579	0.145940
3	6	0	-1.056530	1.095318	0.099608
4	6	0	-3.195171	-1.923476	0.081949
5	6	0	-3.613423	1.001500	-0.252972
6	6	0	-0.069083	0.070145	0.329004
7	6	0	-4.551968	-1.342450	-0.184575
8	8	0	-3.060324	-3.155398	0.235607
9	6	0	-0.721166	-1.195724	0.350740
10	6	0	-4.749573	0.053878	-0.339547
11	8	0	-3.823849	2.258013	-0.396879
12	6	0	-5.654283	-2.204702	-0.276680
13	6	0	-6.044695	0.542441	-0.581286
14	6	0	-6.933843	-1.707966	-0.518665
15	1	0	-5.484225	-3.269179	-0.154158
16	6	0	-7.128821	-0.327856	-0.670888
17	1	0	-6.179943	1.612353	-0.696547
18	1	0	-7.776980	-2.389122	-0.588286
19	1	0	-8.124212	0.064402	-0.858616
20	6	0	-0.065877	-2.530665	0.557472
21	1	0	0.947564	-2.494383	0.150532
22	1	0	-0.640329	-3.291210	0.023188
23	6	0	1.681186	1.826726	0.380132
24	6	0	0.683899	2.822569	0.152313
25	1	0	3.781404	1.473954	0.675696
26	6	0	3.022572	2.228851	0.505682
27	6	0	1.069913	4.177529	0.061788
28	6	0	2.402739	4.547362	0.189694
29	6	0	3.386057	3.568657	0.412129
30	1	0	0.305184	4.926431	-0.110558
31	1	0	2.682824	5.594266	0.116797
32	1	0	4.428879	3.855085	0.510868
33	6	0	-0.709020	2.445038	0.012395
34	6	0	1.324913	0.388709	0.489508
35	8	0	2.208503	-0.488136	0.716032
36	8	0	-1.596453	3.421265	-0.199179
37	1	0	-2.542640	3.010454	-0.287329
38	6	0	0.013808	-2.928137	2.047200
39	1	0	-0.986063	-2.993009	2.487930
40	1	0	0.496218	-3.906295	2.153513
41	1	0	0.597203	-2.197267	2.615804
42	6	0	5.805257	-1.647289	-0.883535
43	6	0	5.140874	-2.046889	0.429071

44	1	0	4.280689	-2.678155	0.176252
45	1	0	5.868696	-2.653404	0.978347
46	8	0	4.786594	-0.927853	1.195512
47	1	0	3.845537	-0.682503	1.001133
48	9	0	6.167238	-2.750332	-1.596044
49	9	0	6.932132	-0.914189	-0.693976
50	9	0	4.990802	-0.910789	-1.680310

Rotational constants (GHZ): 0.2097408 0.0663224 0.0531759

- Geometry optimization of **KuQ**$^{\cdot-}$ **+ 2 TFE** (on carbonyl oxygens B-C) in the *vacuum* (B3LYP functional 6-31G+(d,p) basis set, BSSE and counterpoise correction)

Energy -2091.613441 Hartree
Stoichiometry C27H20F6O6(1-,2)
Framework group C1[X(C27H20F6O6)]
Deg. of freedom 171
Full point group C1 NOp 1
Largest Abelian subgroup C1 NOp 1
Largest concise Abelian subgroup C1 NOp 1

Standard orientation:

Center Number	Atomic Number	Atomic Type	Coordinates (Angstroms) X	Y	Z
1	6	0	0.716223	2.202600	0.107724
2	6	0	1.127300	0.832664	0.216008
3	6	0	-0.706922	2.206780	-0.094746
4	6	0	2.506156	0.470197	0.427365
5	6	0	1.612948	3.294620	0.208770
6	6	0	-1.143702	0.831585	-0.107609
7	6	0	3.469802	1.607068	0.542919
8	8	0	2.902234	-0.721228	0.507000
9	6	0	-0.016949	-0.007423	0.091899
10	6	0	3.037543	2.955905	0.436673
11	8	0	1.259959	4.519221	0.117417
12	6	0	4.833293	1.343624	0.751616
13	6	0	3.977365	3.993706	0.547149
14	6	0	5.754125	2.382784	0.860080
15	1	0	5.165095	0.314190	0.824011
16	6	0	5.324120	3.713469	0.758237
17	1	0	3.618270	5.013867	0.461688
18	1	0	6.804694	2.157388	1.021658
19	1	0	6.041077	4.526225	0.841875
20	6	0	-0.028327	-1.506631	0.159104
21	1	0	-0.990782	-1.832248	0.561652
22	1	0	0.774437	-1.835618	0.823886
23	6	0	-3.471903	1.621000	-0.446998
24	6	0	-3.011810	2.971286	-0.427697
25	1	0	-5.195036	0.348208	-0.614748
26	6	0	-4.845559	1.374316	-0.607227
27	6	0	-3.944200	4.019783	-0.576402
28	6	0	-5.296992	3.748594	-0.735894
29	6	0	-5.752406	2.419967	-0.749578
30	1	0	-3.578380	5.040392	-0.561361
31	1	0	-6.002712	4.567634	-0.848289
32	1	0	-6.810412	2.204762	-0.870377

33	6	0	-1.601080	3.267252	-0.252179
34	6	0	-2.527363	0.479582	-0.302492
35	8	0	-2.933952	-0.713113	-0.355375
36	8	0	-1.238819	4.549106	-0.245824
37	1	0	-0.216095	4.620420	-0.102930
38	6	0	0.174131	-2.159445	-1.225010
39	1	0	1.134281	-1.862195	-1.658482
40	1	0	0.163903	-3.252284	-1.134318
41	1	0	-0.626654	-1.868235	-1.911680
42	6	0	-5.891408	-3.523667	0.797891
43	6	0	-4.801847	-3.411986	-0.263283
44	1	0	-3.841269	-3.410611	0.266412
45	1	0	-4.866190	-4.327527	-0.866171
46	8	0	-4.974524	-2.285094	-1.069080
47	1	0	-4.279774	-1.620488	-0.833866
48	9	0	-5.709689	-4.655989	1.542547
49	9	0	-7.134687	-3.606526	0.262527
50	9	0	-5.899895	-2.481669	1.660132
51	6	0	5.871199	-3.504385	-0.693218
52	6	0	4.758663	-3.436104	0.347584
53	1	0	3.809833	-3.406560	-0.201941
54	1	0	4.806863	-4.377341	0.910718
55	8	0	4.917616	-2.347397	1.207998
56	1	0	4.238511	-1.666201	0.981638
57	9	0	5.702576	-4.601004	-1.491761
58	9	0	7.101835	-3.615924	-0.134534
59	9	0	5.901619	-2.424288	-1.506753

Rotational constants (GHZ): 0.0975683 0.0465500 0.0329427

- Geometry optimization of **KuQ**$^{\cdot-}$ **+ 2 TFE** (on carbonyl oxygens B-C) in DCM (B3LYP functional 6-31G+(d,p) basis set)

Energy -2091.667937 Hartree
Stoichiometry C27H20F6O6(1-,2)
Framework group C1[X(C27H20F6O6)]
Deg. of freedom 171
Full point group C1 NOp 1
Largest Abelian subgroup C1 NOp 1
Largest concise Abelian subgroup C1 NOp 1
Standard orientation:

Center Number	Atomic Number	Atomic Type	Coordinates (Angstroms) X	Y	Z
1	6	0	0.729398	2.079121	0.130183
2	6	0	1.140500	0.712158	0.272321
3	6	0	-0.685462	2.073972	-0.131379
4	6	0	2.511187	0.360454	0.547117
5	6	0	1.619176	3.173046	0.254022
6	6	0	-1.115174	0.696647	-0.145700
7	6	0	3.464250	1.500509	0.689991
8	8	0	2.904316	-0.830294	0.660578
9	6	0	0.006713	-0.135784	0.109585
10	6	0	3.032743	2.847201	0.546150
11	8	0	1.259863	4.397720	0.130246
12	6	0	4.817431	1.246015	0.966682

13	6	0	3.965235	3.888888	0.684533
14	6	0	5.730409	2.289414	1.103508
15	1	0	5.149434	0.219896	1.072375
16	6	0	5.302372	3.616783	0.961616
17	1	0	3.617153	4.909668	0.570099
18	1	0	6.772273	2.071273	1.318489
19	1	0	6.011629	4.432640	1.066589
20	6	0	0.002178	-1.634502	0.190641
21	1	0	-0.983127	-1.967177	0.524273
22	1	0	0.753512	-1.950697	0.918728
23	6	0	-3.431805	1.472660	-0.590220
24	6	0	-2.979445	2.825642	-0.568976
25	1	0	-5.141242	0.190596	-0.822958
26	6	0	-4.796079	1.217783	-0.809681
27	6	0	-3.909202	3.867239	-0.772041
28	6	0	-5.253232	3.587774	-0.988326
29	6	0	-5.700968	2.256926	-1.006288
30	1	0	-3.556253	4.892143	-0.755383
31	1	0	-5.956331	4.401103	-1.142456
32	1	0	-6.751038	2.036249	-1.172853
33	6	0	-1.578219	3.126726	-0.337599
34	6	0	-2.489884	0.339357	-0.389102
35	8	0	-2.890312	-0.856954	-0.437223
36	8	0	-1.215532	4.410743	-0.329962
37	1	0	-0.197240	4.479797	-0.146767
38	6	0	0.318720	-2.301991	-1.165371
39	1	0	1.309368	-2.006158	-1.524030
40	1	0	0.304795	-3.392733	-1.062085
41	1	0	-0.421972	-2.023037	-1.921467
42	6	0	-5.990723	-3.156719	0.963101
43	6	0	-4.980341	-3.468211	-0.135078
44	1	0	-4.014060	-3.639073	0.354176
45	1	0	-5.302834	-4.402940	-0.605207
46	8	0	-4.929833	-2.452270	-1.100275
47	1	0	-4.225159	-1.801078	-0.855547
48	9	0	-6.043103	-4.169816	1.871666
49	9	0	-7.249273	-2.993809	0.481599
50	9	0	-5.686443	-2.025352	1.647648
51	6	0	5.846477	-3.260541	-0.860460
52	6	0	4.939949	-3.495178	0.342611
53	1	0	3.932141	-3.686499	-0.044483
54	1	0	5.300891	-4.402096	0.838299
55	8	0	4.981969	-2.422191	1.245018
56	1	0	4.266092	-1.777331	1.022315
57	9	0	5.804826	-4.324511	-1.709429
58	9	0	7.145775	-3.088527	-0.507796
59	9	0	5.489440	-2.165865	-1.578156

Rotational constants (GHZ): 0.1058467 0.0460058 0.0340169

- Geometry optimization of **KuQ**$^{2-}$ in the *vacuum* (B3LYP functional 6-31G+(d,p) basis set)

Energy -1185.937687 Hartree
Stoichiometry C23H14O4(2-)
Framework group C1[X(C23H14O4)]
Deg. of freedom 117

Full point group   C1   NOp   1
Largest Abelian subgroup   C1   NOp   1
Largest concise Abelian subgroup C1   NOp   1

Standard orientation:

Center Number	Atomic Number	Atomic Type	Coordinates (Angstroms) X	Y	Z
1	6	0	0.727901	-0.433298	-0.012360
2	6	0	1.155641	0.952376	-0.118111
3	6	0	-0.727218	-0.428116	-0.014417
4	6	0	2.542710	1.334606	-0.149039
5	6	0	1.615166	-1.515765	0.058557
6	6	0	-1.153010	0.959347	-0.122836
7	6	0	3.497503	0.188410	-0.087022
8	8	0	2.980607	2.519367	-0.216744
9	6	0	0.004972	1.780667	-0.191665
10	6	0	3.048195	-1.163588	0.015693
11	8	0	1.274242	-2.767097	0.151974
12	6	0	4.878327	0.450293	-0.125395
13	6	0	4.014068	-2.192511	0.075354
14	6	0	5.816697	-0.577192	-0.067355
15	1	0	5.179422	1.491809	-0.201701
16	6	0	5.375219	-1.912091	0.034750
17	1	0	3.643544	-3.210452	0.153746
18	1	0	6.881794	-0.351131	-0.099819
19	1	0	6.100020	-2.724519	0.081998
20	6	0	0.008649	3.278331	-0.325328
21	1	0	-0.897445	3.583157	-0.857274
22	1	0	0.901227	3.578913	-0.883093
23	6	0	-3.491796	0.204788	-0.089285
24	6	0	-3.038550	-1.158611	0.017230
25	1	0	-5.171562	1.505721	-0.203925
26	6	0	-4.873881	0.463136	-0.125080
27	6	0	-4.020127	-2.187501	0.081106
28	6	0	-5.374746	-1.898057	0.042217
29	6	0	-5.817010	-0.557916	-0.062515
30	1	0	-3.671775	-3.212504	0.161380
31	1	0	-6.101623	-2.708757	0.092814
32	1	0	-6.881492	-0.329792	-0.092966
33	6	0	-1.640711	-1.471285	0.056365
34	6	0	-2.543967	1.341933	-0.156720
35	8	0	-2.967338	2.535551	-0.233666
36	8	0	-1.291242	-2.786745	0.159001
37	1	0	-0.265409	-2.850693	0.163909
38	6	0	0.030435	3.991809	1.042675
39	1	0	0.928769	3.714017	1.604351
40	1	0	0.034426	5.083552	0.909097
41	1	0	-0.856860	3.723485	1.626042

Rotational constants (GHZ): 0.3784319 0.1309486 0.0982974

- Geometry optimization of **KuQ**$^{2-}$ in DCM (B3LYP functional 6-31G+(d,p) basis set)

Energy -1186.142285 Hartree
Stoichiometry   C23H14O4(2-)

```
 Framework group  C1[X(C23H14O4)]
 Deg. of freedom   117
 Full point group                 C1      NOp   1
 Largest Abelian subgroup         C1      NOp   1
 Largest concise Abelian subgroup C1      NOp   1
                         Standard orientation:
```

Center Number	Atomic Number	Atomic Type	Coordinates (Angstroms) X	Y	Z
1	6	0	0.727001	-0.432676	-0.021087
2	6	0	1.154661	0.953927	-0.126535
3	6	0	-0.730326	-0.426031	-0.021716
4	6	0	2.543114	1.326301	-0.164603
5	6	0	1.613253	-1.508145	0.051795
6	6	0	-1.154898	0.963062	-0.125573
7	6	0	3.497312	0.184223	-0.091120
8	8	0	2.977705	2.513960	-0.249952
9	6	0	0.006494	1.784944	-0.193235
10	6	0	3.046798	-1.166471	0.014620
11	8	0	1.258798	-2.765509	0.146609
12	6	0	4.880396	0.443081	-0.119829
13	6	0	4.010673	-2.195901	0.085097
14	6	0	5.814867	-0.585405	-0.048736
15	1	0	5.199144	1.477767	-0.198031
16	6	0	5.372361	-1.917020	0.054544
17	1	0	3.653398	-3.217207	0.164816
18	1	0	6.878180	-0.361236	-0.072491
19	1	0	6.094230	-2.728211	0.111234
20	6	0	0.014216	3.283197	-0.312860
21	1	0	-0.893087	3.597348	-0.834755
22	1	0	0.895064	3.585854	-0.885904
23	6	0	-3.493695	0.207179	-0.086911
24	6	0	-3.044228	-1.154790	0.014447
25	1	0	-5.194796	1.500330	-0.188039
26	6	0	-4.879871	0.463998	-0.113372
27	6	0	-4.021932	-2.184811	0.081656
28	6	0	-5.376926	-1.896596	0.052501
29	6	0	-5.817651	-0.558142	-0.045748
30	1	0	-3.681055	-3.211885	0.157279
31	1	0	-6.102449	-2.705001	0.106055
32	1	0	-6.880397	-0.331377	-0.068335
33	6	0	-1.642898	-1.463947	0.046650
34	6	0	-2.544087	1.338506	-0.158939
35	8	0	-2.968308	2.537480	-0.240765
36	8	0	-1.285876	-2.782213	0.141246
37	1	0	-0.254333	-2.834725	0.149101
38	6	0	0.060603	3.989554	1.058446
39	1	0	0.965190	3.711765	1.609043
40	1	0	0.061128	5.078995	0.930853
41	1	0	-0.810164	3.719618	1.665114

```
 Rotational constants (GHZ):           0.3785550           0.1309074
0.0983085
```

- Geometry optimization of **KuQ^{2-} + 1 TFE** (on carbonyl oxygen A) in the *vacuum* (B3LYP functional 6-31G+(d,p) basis set, BSSE and counterpoise correction)

Energy -1638.772153 Hartree
Stoichiometry C25H17F3O5(2-)
Framework group C1[X(C25H17F3O5)]
Deg. of freedom 144
Full point group C1 NOp 1
Largest Abelian subgroup C1 NOp 1
Largest concise Abelian subgroup C1 NOp 1
Standard orientation:

Center Number	Atomic Number	Atomic Type	Coordinates (Angstroms) X	Y	Z
1	6	0	0.589089	0.965003	0.349222
2	6	0	1.587976	1.953595	-0.029116
3	6	0	1.259729	-0.334844	0.340817
4	6	0	1.287841	3.357872	-0.121138
5	6	0	-0.731531	1.291661	0.641996
6	6	0	2.639653	-0.102169	-0.057330
7	6	0	-0.117128	3.709773	0.233702
8	8	0	2.101039	4.260950	-0.467220
9	6	0	2.818817	1.291690	-0.267324
10	6	0	-1.078204	2.715345	0.596525
11	8	0	-1.661810	0.407182	0.973226
12	6	0	-0.502535	5.061283	0.207497
13	6	0	-2.388024	3.136533	0.924834
14	6	0	-1.796925	5.455484	0.532544
15	1	0	0.257939	5.783045	-0.077872
16	6	0	-2.745133	4.478841	0.895498
17	1	0	-3.111384	2.378066	1.204613
18	1	0	-2.074224	6.508142	0.506451
19	1	0	-3.761759	4.772788	1.152167
20	6	0	4.105626	1.954048	-0.672789
21	1	0	4.939900	1.363282	-0.283202
22	1	0	4.124093	2.966575	-0.258783
23	6	0	3.071351	-2.512776	0.166600
24	6	0	1.707392	-2.710443	0.567949
25	1	0	4.953194	-3.430682	-0.215412
26	6	0	3.929557	-3.624213	0.094058
27	6	0	1.280995	-4.029103	0.879731
28	6	0	2.151890	-5.105251	0.798758
29	6	0	3.493121	-4.908561	0.402237
30	1	0	0.249131	-4.170761	1.184454
31	1	0	1.796983	-6.105929	1.042357
32	1	0	4.176941	-5.753362	0.338592
33	6	0	0.797965	-1.600278	0.656291
34	6	0	3.601887	-1.168013	-0.175152
35	8	0	4.806702	-1.025165	-0.537231
36	8	0	-0.476385	-1.871652	1.065392
37	1	0	-0.993773	-0.992852	1.081580
38	6	0	4.255791	2.052740	-2.205284
39	1	0	3.438674	2.643480	-2.632690
40	1	0	5.204698	2.541580	-2.467895
41	1	0	4.251845	1.053829	-2.654000
42	6	0	-4.932797	-2.173869	-0.793617
43	6	0	-3.674745	-1.323370	-0.813005
44	1	0	-2.882691	-1.912586	-0.330657
45	1	0	-3.406542	-1.210065	-1.877668
46	8	0	-3.913054	-0.106123	-0.180843

47	1	0	-3.049351	0.186792	0.269156
48	9	0	-4.728939	-3.354679	-1.459875
49	9	0	-5.992115	-1.576344	-1.407530
50	9	0	-5.349564	-2.505504	0.453094

Rotational constants (GHZ): 0.1224626 0.1019383 0.0595638

- Geometry optimization of **KuQ^{2-} + 1 TFE** (on carbonyl oxygen B) in the *vacuum* (B3LYP functional 6-31G+(d,p) basis set, BSSE and counterpoise correction)

Energy -1638.771095 Hartree
Stoichiometry C25H17F3O5(2-)
Framework group C1[X(C25H17F3O5)]
Deg. of freedom 144
Full point group C1 NOp 1
Largest Abelian subgroup C1 NOp 1
Largest concise Abelian subgroup C1 NOp 1

Standard orientation:

Center Number	Atomic Number	Atomic Type	Coordinates (Angstroms) X	Y	Z
1	6	0	1.260871	1.177200	0.001666
2	6	0	0.142720	0.257472	-0.087411
3	6	0	2.473590	0.377385	-0.006464
4	6	0	-1.212670	0.699046	-0.111965
5	6	0	1.112454	2.568564	0.063832
6	6	0	2.067141	-1.018343	-0.098980
7	6	0	-1.398647	2.161676	-0.043124
8	8	0	-2.221801	-0.099736	-0.175880
9	6	0	0.656537	-1.076121	-0.156100
10	6	0	-0.277845	3.051924	0.039849
11	8	0	2.089055	3.424264	0.138154
12	6	0	-2.698953	2.710396	-0.046704
13	6	0	-0.518184	4.441958	0.104212
14	6	0	-2.911231	4.082419	0.018608
15	1	0	-3.545532	2.033835	-0.097126
16	6	0	-1.807426	4.957027	0.092972
17	1	0	0.353496	5.086802	0.164602
18	1	0	-3.926091	4.475815	0.013343
19	1	0	-1.965870	6.033531	0.143991
20	6	0	-0.162136	-2.330849	-0.273444
21	1	0	0.441572	-3.096332	-0.768566
22	1	0	-1.057005	-2.111936	-0.864101
23	6	0	4.446936	-1.666091	-0.084010
24	6	0	4.809686	-0.278699	0.004038
25	1	0	5.141221	-3.675531	-0.186816
26	6	0	5.460242	-2.638592	-0.122346
27	6	0	6.191651	0.050684	0.048007
28	6	0	7.168750	-0.932407	0.006927
29	6	0	6.808616	-2.295600	-0.079575
30	1	0	6.458753	1.100430	0.114596
31	1	0	8.220274	-0.649023	0.041868
32	1	0	7.575855	-3.067204	-0.111707
33	6	0	3.806278	0.751032	0.045445
34	6	0	3.027295	-2.101258	-0.129812

35	8	0	2.731183	-3.327630	-0.185911
36	8	0	4.234355	2.039284	0.130182
37	1	0	3.406723	2.659272	0.139674
38	6	0	-0.606733	-2.873251	1.101044
39	1	0	-1.214686	-2.130701	1.628390
40	1	0	-1.206685	-3.786271	0.980013
41	1	0	0.266408	-3.120116	1.714334
42	6	0	-6.306593	-1.763431	0.073198
43	6	0	-4.898420	-1.591370	-0.471282
44	1	0	-4.209312	-1.923530	0.316873
45	1	0	-4.814983	-2.301008	-1.312930
46	8	0	-4.678989	-0.272760	-0.850090
47	1	0	-3.711333	-0.058040	-0.617748
48	9	0	-6.537195	-3.076195	0.402057
49	9	0	-7.271691	-1.427553	-0.825575
50	9	0	-6.551869	-1.040920	1.192207

Rotational constants (GHZ): 0.1998166 0.0638267
0.0491871

- Geometry optimization of **KuQ^{2-} + 1 TFE** (on carbonyl oxygen C) in the *vacuum* (B3LYP functional 6-31G+(d,p) basis set, BSSE and counterpoise correction)

Energy -1638.772964 Hartree
Stoichiometry C25H17F3O5(2-)
Framework group C1[X(C25H17F3O5)]
Deg. of freedom 144
Full point group C1 NOp 1
Largest Abelian subgroup C1 NOp 1
Largest concise Abelian subgroup C1 NOp 1
Standard orientation:

Center Number	Atomic Number	Atomic Type	Coordinates (Angstroms) X	Y	Z
1	6	0	-2.476280	0.384977	-0.024780
2	6	0	-2.070170	-1.010260	-0.027042
3	6	0	-1.257625	1.176359	0.005872
4	6	0	-3.026133	-2.092529	-0.054979
5	6	0	-3.812127	0.804260	-0.061584
6	6	0	-0.138113	0.249797	0.019321
7	6	0	-4.457099	-1.656859	-0.110177
8	8	0	-2.744876	-3.318888	-0.031231
9	6	0	-0.659214	-1.082751	-0.008222
10	6	0	-4.820760	-0.279685	-0.107724
11	8	0	-4.217936	2.034875	-0.063191
12	6	0	-5.463507	-2.635174	-0.158076
13	6	0	-6.190361	0.056486	-0.153442
14	6	0	-6.811699	-2.286433	-0.205208
15	1	0	-5.144979	-3.674005	-0.155862
16	6	0	-7.173648	-0.926231	-0.201862
17	1	0	-6.439023	1.113461	-0.149556
18	1	0	-7.577706	-3.059300	-0.243293
19	1	0	-8.224695	-0.641915	-0.237054
20	6	0	0.153940	-2.346952	-0.019893
21	1	0	1.096920	-2.151277	-0.538259
22	1	0	-0.411122	-3.123619	-0.543678

23	6	0	1.408306	2.143115	0.044090
24	6	0	0.279235	3.045061	0.031827
25	1	0	3.555144	2.014405	0.042665
26	6	0	2.710183	2.695000	0.043716
27	6	0	0.532422	4.445785	0.031096
28	6	0	1.821012	4.947693	0.035201
29	6	0	2.928503	4.064490	0.038726
30	1	0	-0.325213	5.110864	0.024983
31	1	0	1.983408	6.024973	0.033487
32	1	0	3.944165	4.455269	0.037441
33	6	0	-1.061928	2.550806	0.016501
34	6	0	1.220800	0.692750	0.053179
35	8	0	2.221858	-0.126591	0.086146
36	8	0	-2.075584	3.463789	0.009458
37	1	0	-2.966055	2.960910	-0.017176
38	6	0	0.475981	-2.854460	1.401350
39	1	0	-0.447621	-3.070583	1.948923
40	1	0	1.069685	-3.778582	1.356320
41	1	0	1.051849	-2.104426	1.953344
42	6	0	6.306357	-1.769939	-0.298433
43	6	0	4.901333	-1.606820	0.256751
44	1	0	4.209305	-1.920073	-0.537409
45	1	0	4.819610	-2.338418	1.080127
46	8	0	4.685779	-0.298661	0.666303
47	1	0	3.702632	-0.086640	0.469711
48	9	0	6.531011	-3.075321	-0.662872
49	9	0	7.278149	-1.461701	0.603093
50	9	0	6.550097	-1.020550	-1.400426

Rotational constants (GHZ): 0.1988282 0.0637156 0.0491835

- Geometry optimization of **KuQ^{2-} + 1 TFE** (on carbonyl oxygen B) in DCM (B3LYP functional 6-31G+(d,p) basis set)

Energy -1638.962066 Hartree
Stoichiometry C25H17F3O5(2-)
Framework group C1[X(C25H17F3O5)]
Deg. of freedom 144
Full point group C1 NOp 1
Largest Abelian subgroup C1 NOp 1
Largest concise Abelian subgroup C1 NOp 1
Standard orientation:

Center Number	Atomic Number	Atomic Type	Coordinates (Angstroms) X	Y	Z
1	6	0	1.141449	1.121738	0.013350
2	6	0	0.105638	0.106141	-0.053228
3	6	0	2.420183	0.425225	-0.001744
4	6	0	-1.281798	0.436550	-0.075101
5	6	0	0.872226	2.490041	0.060547
6	6	0	2.131849	-1.001336	-0.069626
7	6	0	-1.589652	1.879557	-0.008403
8	8	0	-2.223149	-0.437520	-0.134008
9	6	0	0.726600	-1.178683	-0.109013
10	6	0	-0.549520	2.861589	0.055115
11	8	0	1.786143	3.428795	0.109980

12	6	0	-2.933051	2.312230	0.008986
13	6	0	-0.909038	4.226127	0.118807
14	6	0	-3.262475	3.660645	0.072978
15	1	0	-3.718201	1.564771	-0.024403
16	6	0	-2.238661	4.626415	0.126464
17	1	0	-0.106082	4.954233	0.166497
18	1	0	-4.305341	3.966301	0.084795
19	1	0	-2.488738	5.683298	0.177477
20	6	0	0.009346	-2.496507	-0.186050
21	1	0	0.671028	-3.231243	-0.650366
22	1	0	-0.890533	-2.373993	-0.795894
23	6	0	4.558196	-1.442432	-0.079346
24	6	0	4.805951	-0.030592	-0.017218
25	1	0	5.440119	-3.389780	-0.164193
26	6	0	5.654037	-2.326196	-0.119517
27	6	0	6.152734	0.417930	0.000455
28	6	0	7.210415	-0.478229	-0.041940
29	6	0	6.965430	-1.866141	-0.102704
30	1	0	6.339375	1.485544	0.048328
31	1	0	8.232452	-0.107239	-0.027528
32	1	0	7.794346	-2.568070	-0.135742
33	6	0	3.713924	0.907664	0.025943
34	6	0	3.181677	-1.991676	-0.096695
35	8	0	2.987805	-3.244784	-0.128050
36	8	0	4.025355	2.234945	0.088836
37	1	0	3.139080	2.774734	0.101902
38	6	0	-0.415282	-3.015794	1.203797
39	1	0	-1.100735	-2.311169	1.684443
40	1	0	-0.925410	-3.982521	1.114514
41	1	0	0.455409	-3.150913	1.854194
42	6	0	-6.086724	-1.619491	0.101114
43	6	0	-5.019029	-1.823911	-0.967888
44	1	0	-4.264852	-2.496663	-0.541261
45	1	0	-5.510291	-2.338600	-1.802170
46	8	0	-4.475723	-0.609978	-1.399275
47	1	0	-3.609773	-0.450451	-0.908275
48	9	0	-6.614785	-2.814527	0.493338
49	9	0	-7.125734	-0.860282	-0.336584
50	9	0	-5.608575	-1.014647	1.216948

Rotational constants (GHZ): 0.2185994 0.0648961 0.0511622

- Geometry optimization of **KuQ^{2-} + 1 TFE** (on carbonyl oxygen C) in DCM (B3LYP functional 6-31G+(d,p) basis set)

Energy -1638.963439 Hartree
Stoichiometry C25H17F3O5(2-)
Framework group C1[X(C25H17F3O5)]
Deg. of freedom 144
Full point group C1 NOp 1
Largest Abelian subgroup C1 NOp 1
Largest concise Abelian subgroup C1 NOp 1

Standard orientation:

Center Number	Atomic Number	Atomic Type	Coordinates (Angstroms) X	Y	Z

1	6	0	-2.420714	0.429990	-0.021059
2	6	0	-2.126134	-0.993458	-0.077408
3	6	0	-1.140776	1.123278	-0.000222
4	6	0	-3.171892	-1.987626	-0.114101
5	6	0	-3.715732	0.949378	-0.012232
6	6	0	-0.099859	0.110026	-0.050152
7	6	0	-4.561269	-1.440957	-0.115356
8	8	0	-2.985129	-3.235148	-0.138598
9	6	0	-0.725240	-1.177669	-0.101381
10	6	0	-4.813641	-0.039722	-0.063332
11	8	0	-4.011489	2.219725	0.031630
12	6	0	-5.647008	-2.332911	-0.163059
13	6	0	-6.151604	0.406231	-0.062814
14	6	0	-6.961734	-1.874667	-0.163475
15	1	0	-5.429128	-3.395689	-0.199744
16	6	0	-7.212480	-0.492593	-0.112598
17	1	0	-6.329028	1.475911	-0.021470
18	1	0	-7.787075	-2.580576	-0.202394
19	1	0	-8.235494	-0.124545	-0.111376
20	6	0	-0.007851	-2.496678	-0.166803
21	1	0	0.905057	-2.372494	-0.756178
22	1	0	-0.658207	-3.229579	-0.650372
23	6	0	1.589996	1.879087	-0.024989
24	6	0	0.541462	2.866502	0.032260
25	1	0	3.717644	1.576423	-0.124270
26	6	0	2.932900	2.323246	-0.068158
27	6	0	0.905734	4.241468	0.062333
28	6	0	2.230750	4.637572	0.026320
29	6	0	3.260254	3.669869	-0.044954
30	1	0	0.112145	4.979437	0.111761
31	1	0	2.480541	5.695666	0.048977
32	1	0	4.301143	3.980130	-0.081755
33	6	0	-0.834543	2.473272	0.049847
34	6	0	1.287575	0.446927	-0.047019
35	8	0	2.229579	-0.439771	-0.071915
36	8	0	-1.780167	3.461271	0.110540
37	1	0	-2.707331	3.017905	0.085510
38	6	0	0.386656	-3.025185	1.228370
39	1	0	-0.496089	-3.157540	1.863084
40	1	0	0.892511	-3.994827	1.144466
41	1	0	1.067519	-2.326493	1.724015
42	6	0	6.075775	-1.629475	-0.290017
43	6	0	4.993690	-1.851659	0.760981
44	1	0	4.245118	-2.515124	0.309932
45	1	0	5.474212	-2.385497	1.590040
46	8	0	4.445942	-0.647141	1.208095
47	1	0	3.578656	-0.477864	0.708256
48	9	0	6.607844	-2.818219	-0.697808
49	9	0	7.110870	-0.880439	0.174342
50	9	0	5.613733	-1.003809	-1.401245

Rotational constants (GHZ): 0.2173669 0.0650776 0.0512086

- Geometry optimization of **KuQ^{2-} + 2 TFE** (on carbonyl oxygens B-C) in the *vacuum* (B3LYP functional 6-31G+(d,p) basis set, BSSE and counterpoise correction)

Energy -2091.602391 Hartree
Stoichiometry C27H20F6O6(2-)
Framework group C1[X(C27H20F6O6)]
Deg. of freedom 171
Full point group C1 NOp 1
Largest Abelian subgroup C1 NOp 1
Largest concise Abelian subgroup C1 NOp 1

Standard orientation:

Center Number	Atomic Number	Atomic Type	Coordinates (Angstroms) X	Y	Z
1	6	0	0.724242	-2.451599	-0.052965
2	6	0	1.147438	-1.066073	-0.117833
3	6	0	-0.723819	-2.447906	0.043948
4	6	0	2.524534	-0.689925	-0.223737
5	6	0	1.612517	-3.532647	-0.099213
6	6	0	-1.155024	-1.058815	0.035328
7	6	0	3.486592	-1.815152	-0.275132
8	8	0	2.927200	0.524172	-0.262231
9	6	0	-0.003734	-0.230254	-0.072974
10	6	0	3.040531	-3.172093	-0.214778
11	8	0	1.276570	-4.783451	-0.050960
12	6	0	4.868342	-1.556782	-0.374081
13	6	0	4.000391	-4.204690	-0.265039
14	6	0	5.798198	-2.590031	-0.422117
15	1	0	5.203100	-0.525574	-0.408096
16	6	0	5.357435	-3.926285	-0.368278
17	1	0	3.627899	-5.223401	-0.217918
18	1	0	6.859381	-2.362527	-0.499299
19	1	0	6.079453	-4.740675	-0.405167
20	6	0	-0.004269	1.270701	-0.136245
21	1	0	-0.940718	1.600584	-0.593657
22	1	0	0.845065	1.597269	-0.743071
23	6	0	-3.486438	-1.803629	0.190244
24	6	0	-3.029391	-3.171166	0.195806
25	1	0	-5.213567	-0.523553	0.233874
26	6	0	-4.875235	-1.554210	0.247562
27	6	0	-4.000188	-4.207518	0.268497
28	6	0	-5.353566	-3.925277	0.326804
29	6	0	-5.802747	-2.584117	0.313948
30	1	0	-3.642478	-5.231885	0.274616
31	1	0	-6.074454	-4.739921	0.380750
32	1	0	-6.866245	-2.358981	0.355516
33	6	0	-1.633278	-3.489890	0.126283
34	6	0	-2.537968	-0.686852	0.124822
35	8	0	-2.929330	0.538416	0.146995
36	8	0	-1.284197	-4.804943	0.141086
37	1	0	-0.261773	-4.872686	0.068430
38	6	0	0.111999	1.918403	1.259609
39	1	0	1.040935	1.609919	1.750679
40	1	0	0.114810	3.014075	1.177528
41	1	0	-0.735213	1.626059	1.888343
42	6	0	-5.589805	4.042810	-0.461886
43	6	0	-4.438054	3.244460	0.125838
44	1	0	-3.732824	3.054654	-0.694685
45	1	0	-3.943402	3.917511	0.847207
46	8	0	-4.904683	2.072867	0.709850
47	1	0	-4.205618	1.357514	0.537168
48	9	0	-5.131575	5.220621	-0.996616

49	9	0	-6.525806	4.388366	0.462375
50	9	0	-6.245894	3.400414	-1.457288
51	6	0	5.584743	4.033169	0.347175
52	6	0	4.425612	3.256006	-0.254436
53	1	0	3.722152	3.048279	0.562431
54	1	0	3.932781	3.947059	-0.959009
55	8	0	4.885217	2.095906	-0.870719
56	1	0	4.204675	1.374656	-0.686786
57	9	0	5.136333	5.200714	0.909332
58	9	0	6.519031	4.392019	-0.573679
59	9	0	6.239629	3.362763	1.324329

Rotational constants (GHZ): 0.0842007 0.0496563 0.0319946

- Geometry optimization of **KuQ^{2-} + 2 TFE** (on carbonyl oxygens A-C) in the *vacuum* (B3LYP functional 6-31G+(d,p) basis set, BSSE and counterpoise correction)

Energy -2091.604811 Hartree
Stoichiometry C27H20F6O6(2-)
Framework group C1[X(C27H20F6O6)]
Deg. of freedom 171
Full point group C1 NOp 1
Largest Abelian subgroup C1 NOp 1
Largest concise Abelian subgroup C1 NOp 1

Standard orientation:

Center Number	Atomic Number	Atomic Type	Coordinates (Angstroms) X	Y	Z
1	6	0	1.161017	1.078263	-0.417191
2	6	0	0.303494	2.214229	-0.113225
3	6	0	0.295438	-0.097308	-0.464504
4	6	0	0.819750	3.557492	0.010312
5	6	0	2.534096	1.190997	-0.606816
6	6	0	-1.056343	0.350487	-0.177629
7	6	0	2.287822	3.682338	-0.236608
8	8	0	0.139645	4.574963	0.300709
9	6	0	-1.026876	1.766946	0.024791
10	6	0	3.100050	2.546847	-0.529315
11	8	0	3.335242	0.174972	-0.869040
12	6	0	2.876355	4.955818	-0.175371
13	6	0	4.479582	2.749794	-0.756775
14	6	0	4.238276	5.136250	-0.400806
15	1	0	2.222065	5.791950	0.054756
16	6	0	5.041947	4.019625	-0.694861
17	1	0	5.092393	1.884570	-0.984962
18	1	0	4.676638	6.131236	-0.349913
19	1	0	6.108386	4.146049	-0.873182
20	6	0	-2.214691	2.634871	0.330383
21	1	0	-3.095364	2.205494	-0.155640
22	1	0	-2.022050	3.640470	-0.053520
23	6	0	-1.855023	-1.948490	-0.433752
24	6	0	-0.508709	-2.368529	-0.724002
25	1	0	-3.895816	-2.596531	-0.237043
26	6	0	-2.881537	-2.918623	-0.446984
27	6	0	-0.270380	-3.740838	-1.004266

28	6	0	-1.300642	-4.665177	-1.004042
29	6	0	-2.623660	-4.253239	-0.724870
30	1	0	0.748794	-4.043248	-1.221110
31	1	0	-1.089817	-5.711071	-1.221532
32	1	0	-3.436725	-4.976089	-0.727868
33	6	0	0.571383	-1.425196	-0.738345
34	6	0	-2.167140	-0.545401	-0.135469
35	8	0	-3.366970	-0.162921	0.141381
36	8	0	1.815303	-1.900494	-1.037960
37	1	0	2.462808	-1.116730	-1.019384
38	6	0	-2.493956	2.734833	1.844686
39	1	0	-1.634625	3.171291	2.364548
40	1	0	-3.366873	3.375011	2.032854
41	1	0	-2.699944	1.744085	2.262373
42	6	0	-7.797338	-0.057607	0.210734
43	6	0	-6.381389	0.217940	0.688564
44	1	0	-5.918165	0.878194	-0.057318
45	1	0	-6.487803	0.791135	1.625959
46	8	0	-5.682187	-0.970726	0.855660
47	1	0	-4.719130	-0.787850	0.581393
48	9	0	-8.499180	1.116108	0.090660
49	9	0	-8.509683	-0.834502	1.070512
50	9	0	-7.856674	-0.663366	-0.999158
51	6	0	6.146469	-2.883796	0.910410
52	6	0	5.014367	-1.871811	0.956011
53	1	0	4.165820	-2.315931	0.418533
54	1	0	4.731060	-1.785904	2.018806
55	8	0	5.434250	-0.660660	0.410242
56	1	0	4.651442	-0.250077	-0.082285
57	9	0	5.763690	-4.062188	1.495709
58	9	0	7.256052	-2.472655	1.584394
59	9	0	6.551405	-3.192443	-0.345422

Rotational constants (GHZ): 0.1179111 0.0420104 0.0323766

- Geometry optimization of **KuQ^{2-} + 2 TFE** (on carbonyl oxygens A-C) in DCM (B3LYP functional 6-31G+(d,p) basis set)

Energy -2091.784411 Hartree
Stoichiometry C27H20F6O6(2-)
Framework group C1[X(C27H20F6O6)]
Deg. of freedom 171
Full point group C1 NOp 1
Largest Abelian subgroup C1 NOp 1
Largest concise Abelian subgroup C1 NOp 1

Standard orientation:

Center Number	Atomic Number	Atomic Type	Coordinates (Angstroms) X	Y	Z
1	6	0	1.176073	1.029027	-0.400891
2	6	0	0.337304	2.206172	-0.222956
3	6	0	0.277347	-0.121225	-0.459862
4	6	0	0.895749	3.533812	-0.133126
5	6	0	2.560301	1.090223	-0.479594
6	6	0	-1.077008	0.383519	-0.305894

7	6	0	2.379507	3.601402	-0.256823
8	8	0	0.225438	4.589136	0.038056
9	6	0	-1.016169	1.807173	-0.165554
10	6	0	3.171327	2.426186	-0.419296
11	8	0	3.345024	0.031617	-0.617456
12	6	0	3.012604	4.856041	-0.207772
13	6	0	4.571934	2.568341	-0.530865
14	6	0	4.394592	4.975582	-0.318535
15	1	0	2.387586	5.734512	-0.081809
16	6	0	5.177090	3.819287	-0.481876
17	1	0	5.171999	1.674443	-0.660532
18	1	0	4.864330	5.954711	-0.280005
19	1	0	6.257335	3.900876	-0.571626
20	6	0	-2.197151	2.716715	0.022848
21	1	0	-3.051313	2.299466	-0.516371
22	1	0	-1.952777	3.702458	-0.380110
23	6	0	-1.925698	-1.904726	-0.515171
24	6	0	-0.578423	-2.382199	-0.666769
25	1	0	-3.999030	-2.470519	-0.462414
26	6	0	-2.984493	-2.839624	-0.566357
27	6	0	-0.367721	-3.775148	-0.849387
28	6	0	-1.428706	-4.664284	-0.886839
29	6	0	-2.753610	-4.195062	-0.747211
30	1	0	0.650486	-4.131533	-0.962018
31	1	0	-1.240038	-5.725717	-1.027165
32	1	0	-3.587062	-4.891340	-0.782748
33	6	0	0.528620	-1.467797	-0.642296
34	6	0	-2.214107	-0.478261	-0.321898
35	8	0	-3.426512	-0.057111	-0.190008
36	8	0	1.779523	-1.990017	-0.818431
37	1	0	2.451426	-1.223909	-0.765385
38	6	0	-2.591034	2.874859	1.507505
39	1	0	-1.767358	3.300688	2.089662
40	1	0	-3.455082	3.543153	1.604340
41	1	0	-2.857702	1.907199	1.943955
42	6	0	-7.452276	0.046656	0.281200
43	6	0	-6.298158	0.196244	1.266002
44	1	0	-5.805195	1.149056	1.035624
45	1	0	-6.749726	0.272524	2.262665
46	8	0	-5.424002	-0.892738	1.208793
47	1	0	-4.624014	-0.642914	0.643079
48	9	0	-8.309110	1.104485	0.373022
49	9	0	-8.190671	-1.071876	0.506956
50	9	0	-7.040367	-0.014947	-1.009689
51	6	0	5.823006	-2.888248	0.720600
52	6	0	4.819675	-2.021558	1.472431
53	1	0	3.819366	-2.352257	1.166212
54	1	0	4.950057	-2.248563	2.537131
55	8	0	5.036384	-0.658804	1.239251
56	1	0	4.409257	-0.343526	0.514779
57	9	0	5.613339	-4.210845	0.978601
58	9	0	7.109591	-2.623392	1.071958
59	9	0	5.750234	-2.732771	-0.625137

Rotational constants (GHZ): 0.1212444 0.0451606
0.0343027

- Geometry optimization of **KuQ-Sc^{3+}** (Sc^{3+} on carbonyl oxygen A) in the *vacuum* (B3LYP functional LANL2DZ basis set) = do not converge

- Geometry optimization of **KuQ-Sc^{3+}** (Sc^{3+} on carbonyl oxygen B) in the *vacuum* (B3LYP functional LANL2DZ basis set)

```
Energy -1231.067598 Hartree
Stoichiometry    C23H14O4Sc(3+)
 Framework group  C1[X(C23H14O4Sc)]
 Deg. of freedom   120
 Full point group                C1      NOp   1
 Largest Abelian subgroup        C1      NOp   1
 Largest concise Abelian subgroup C1      NOp   1
                         Standard orientation:
```

Center Number	Atomic Number	Atomic Type	Coordinates (Angstroms) X	Y	Z
1	6	0	0.303143	-0.941381	0.016224
2	6	0	0.980809	0.324391	-0.047341
3	6	0	-1.079348	-0.683460	0.010568
4	6	0	2.373011	0.366248	-0.080578
5	6	0	1.055736	-2.199501	0.058056
6	6	0	-1.267373	0.750713	-0.062826
7	6	0	3.197278	-0.811277	-0.048600
8	8	0	2.918386	1.657293	-0.153686
9	6	0	-0.015173	1.386535	-0.094588
10	6	0	2.536462	-2.097738	0.024569
11	8	0	0.443156	-3.311888	0.115503
12	6	0	4.616291	-0.756664	-0.084650
13	6	0	3.308676	-3.264772	0.060147
14	6	0	5.374900	-1.941745	-0.048799
15	1	0	5.127106	0.199991	-0.141641
16	6	0	4.728558	-3.190684	0.023604
17	1	0	2.811280	-4.229341	0.114673
18	1	0	6.458852	-1.893561	-0.077289
19	1	0	5.314298	-4.104933	0.050994
20	6	0	0.206241	2.863671	-0.205083
21	1	0	-0.760477	3.348393	-0.430741
22	1	0	0.812075	3.103658	-1.148053
23	6	0	-3.758562	0.409562	-0.068696
24	6	0	-3.553455	-1.023743	0.002146
25	1	0	-5.220222	1.986315	-0.164751
26	6	0	-5.065398	0.912784	-0.110925
27	6	0	-4.676750	-1.893372	0.028126
28	6	0	-5.980061	-1.367010	-0.015568
29	6	0	-6.175098	0.030104	-0.085256
30	1	0	-4.517968	-2.966069	0.081413
31	1	0	-6.835683	-2.034624	0.004015
32	1	0	-7.183019	0.433718	-0.119807
33	6	0	-2.223445	-1.583015	0.043963
34	6	0	-2.616236	1.359298	-0.095074
35	8	0	-2.737727	2.615849	-0.134360
36	8	0	-2.064718	-2.885448	0.105848
37	1	0	-1.082491	-3.244097	0.124787
38	6	0	0.737075	3.588039	1.110310
39	1	0	1.511580	3.054261	1.713496

40	1	0	0.922199	4.678871	1.000673
41	1	0	-0.104703	3.579558	1.817336
42	21	0	2.677653	3.431406	-0.203329

Rotational constants (GHZ): 0.2507885 0.1205714 0.0821654

- Geometry optimization of **KuQ-Sc^{3+}** (Sc^{3+} on carbonyl oxygen C) in the *vacuum* (B3LYP functional LANL2DZ basis set) = it converges on carbonyl B

- Geometry optimization of **KuQ-Sc^{3+}** (Sc^{3+} on carbonyl oxygen B) in DCM (B3LYP functional LANL2DZ basis set) = it converges on carbonyl C

- Geometry optimization of **KuQ-Sc^{3+}** (Sc^{3+} on carbonyl oxygen C) in DCM (B3LYP functional LANL2DZ basis set)

```
Energy -1231.650596 Hartree
Stoichiometry    C23H14O4Sc(3+)
 Framework group  C1[X(C23H14O4Sc)]
 Deg. of freedom   120
 Full point group                C1      NOp   1
 Largest Abelian subgroup        C1      NOp   1
 Largest concise Abelian subgroup C1     NOp   1
                        Standard orientation:
```

Center Number	Atomic Number	Atomic Type	Coordinates (Angstroms) X	Y	Z
1	6	0	0.159251	-0.863284	-0.074855
2	6	0	0.893989	0.389783	-0.167524
3	6	0	-1.218802	-0.539440	-0.073470
4	6	0	2.285508	0.351970	-0.132291
5	6	0	0.814427	-2.124527	0.005806
6	6	0	-1.362939	0.884490	-0.175149
7	6	0	3.010331	-0.921903	-0.059869
8	8	0	3.038499	1.495928	-0.140190
9	6	0	-0.089196	1.484733	-0.249883
10	6	0	2.274562	-2.151137	0.001244
11	8	0	0.170107	-3.270231	0.086592
12	6	0	4.418600	-0.979390	-0.046827
13	6	0	2.953935	-3.380479	0.067335
14	6	0	5.090700	-2.214864	0.015401
15	1	0	5.006887	-0.072792	-0.089067
16	6	0	4.362167	-3.415833	0.072332
17	1	0	2.378262	-4.297756	0.115776
18	1	0	6.175469	-2.235310	0.020020
19	1	0	4.880465	-4.367413	0.121736
20	6	0	0.163987	2.957427	-0.370522
21	1	0	-0.708939	3.423586	-0.832700
22	1	0	1.021600	3.158892	-1.023323
23	6	0	-3.870200	0.584574	-0.082407
24	6	0	-3.709962	-0.834238	0.016963
25	1	0	-5.283390	2.203201	-0.150070
26	6	0	-5.165416	1.127662	-0.074913
27	6	0	-4.846147	-1.669649	0.123507

28	6	0	-6.132203	-1.111005	0.132131
29	6	0	-6.291155	0.287861	0.031845
30	1	0	-4.705506	-2.742428	0.200476
31	1	0	-7.003317	-1.752400	0.216813
32	1	0	-7.287060	0.719825	0.038658
33	6	0	-2.379536	-1.439649	0.015755
34	6	0	-2.697660	1.514380	-0.183586
35	8	0	-2.857715	2.763680	-0.256718
36	8	0	-2.210979	-2.709907	0.091225
37	1	0	-0.905448	-3.167175	0.093275
38	6	0	0.366636	3.620479	1.028527
39	1	0	1.164532	3.139473	1.606536
40	1	0	0.601706	4.682246	0.898123
41	1	0	-0.554971	3.549419	1.615166
42	21	0	4.091198	2.960720	0.000191

Rotational constants (GHZ): 0.2643157 0.1101406 0.0783915

- Geometry optimization of **KuQ·⁻-Sc³⁺** (Sc^{3+} on carbonyl oxygen B) in the *vacuum* (B3LYP functional LANL2DZ basis set)

Energy -1231.576975 Hartree
Stoichiometry C23H14O4Sc(2+,2)
Framework group C1[X(C23H14O4Sc)]
Deg. of freedom 120
Full point group C1 NOp 1
Largest Abelian subgroup C1 NOp 1
Largest concise Abelian subgroup C1 NOp 1
Standard orientation:

Center Number	Atomic Number	Atomic Type	Coordinates (Angstroms) X	Y	Z
1	6	0	0.288826	-0.929130	-0.013642
2	6	0	0.977140	0.327875	-0.096459
3	6	0	-1.093956	-0.654005	-0.010126
4	6	0	2.380298	0.366086	-0.101172
5	6	0	1.002624	-2.193733	0.045164
6	6	0	-1.269088	0.778943	-0.096853
7	6	0	3.160145	-0.864991	-0.056424
8	8	0	2.998839	1.571668	-0.130696
9	6	0	-0.012342	1.395199	-0.158545
10	6	0	2.482025	-2.131877	0.015326
11	8	0	0.373745	-3.307039	0.115411
12	6	0	4.571887	-0.837989	-0.080078
13	6	0	3.227260	-3.318376	0.060236
14	6	0	5.308665	-2.037987	-0.036957
15	1	0	5.091301	0.113079	-0.134798
16	6	0	4.640829	-3.273977	0.033065
17	1	0	2.703423	-4.267681	0.116201
18	1	0	6.393505	-2.008336	-0.057900
19	1	0	5.209854	-4.197941	0.066804
20	6	0	0.236437	2.878139	-0.317429
21	1	0	-0.682790	3.356710	-0.671821
22	1	0	0.958040	3.035602	-1.164606
23	6	0	-3.763864	0.431583	-0.067091
24	6	0	-3.566978	-0.992231	0.010720

25	1	0	-5.212174	2.014716	-0.155421
26	6	0	-5.069145	0.940286	-0.097357
27	6	0	-4.687990	-1.858881	0.055686
28	6	0	-5.987516	-1.329955	0.022771
29	6	0	-6.177965	0.065744	-0.053715
30	1	0	-4.526239	-2.930247	0.115329
31	1	0	-6.845065	-1.994269	0.056336
32	1	0	-7.184660	0.472450	-0.079375
33	6	0	-2.229080	-1.549398	0.043711
34	6	0	-2.612561	1.386588	-0.112264
35	8	0	-2.770707	2.638021	-0.148023
36	8	0	-2.069645	-2.851945	0.115235
37	1	0	-1.057336	-3.199092	0.127271
38	6	0	0.626300	3.607570	1.011933
39	1	0	1.426003	3.124349	1.621123
40	1	0	0.811462	4.691514	0.871557
41	1	0	-0.231534	3.576541	1.691804
42	21	0	2.871269	3.455907	-0.096235

Rotational constants (GHZ): 0.2485731 0.1200388 0.0815863

- Geometry optimization of **KuQ$^{\cdot-}$-Sc^{3+}** (Sc^{3+} on carbonyl oxygen C) in the *vacuum* (B3LYP functional LANL2DZ basis set) = it converges on carbonyl B

- Geometry optimization of **KuQ$^{\cdot-}$-Sc^{3+}** (Sc^{3+} on carbonyl oxygen B) in DCM (B3LYP functional LANL2DZ basis set) = it converges on carbonyl C

- Geometry optimization of **KuQ$^{\cdot-}$-Sc^{3+}** (Sc^{3+} on carbonyl oxygen C) in DCM (B3LYP functional LANL2DZ basis set)

Energy -1231.880611 Hartree
Stoichiometry C23H14O4Sc(2+,2)
Framework group C1[X(C23H14O4Sc)]
Deg. of freedom 120
Full point group C1 NOp 1
Largest Abelian subgroup C1 NOp 1
Largest concise Abelian subgroup C1 NOp 1

Standard orientation:

Center Number	Atomic Number	Atomic Type	Coordinates (Angstroms) X	Y	Z
1	6	0	0.142229	-0.864475	-0.001539
2	6	0	0.879475	0.382930	-0.047900
3	6	0	-1.256981	-0.537304	-0.012868
4	6	0	2.292413	0.351074	-0.075185
5	6	0	0.792499	-2.113527	0.045319
6	6	0	-1.373510	0.887863	-0.053909
7	6	0	2.999747	-0.927983	-0.018582
8	8	0	3.030777	1.482266	-0.165767
9	6	0	-0.080044	1.472922	-0.067916
10	6	0	2.248364	-2.150890	0.047062
11	8	0	0.138854	-3.280420	0.093022
12	6	0	4.415087	-1.009883	-0.021210
13	6	0	2.920087	-3.393502	0.110419

14	6	0	5.065705	-2.249124	0.043337
15	1	0	5.019330	-0.111446	-0.071407
16	6	0	4.318892	-3.447237	0.110527
17	1	0	2.329297	-4.301077	0.159967
18	1	0	6.150896	-2.287486	0.042997
19	1	0	4.828321	-4.404262	0.161957
20	6	0	0.211930	2.948487	-0.040438
21	1	0	-0.697542	3.493796	-0.299099
22	1	0	0.956815	3.198960	-0.805195
23	6	0	-3.884750	0.623510	-0.082111
24	6	0	-3.742318	-0.794923	-0.038727
25	1	0	-5.271382	2.265460	-0.160178
26	6	0	-5.174092	1.185387	-0.127445
27	6	0	-4.889064	-1.615972	-0.039223
28	6	0	-6.170677	-1.042286	-0.085637
29	6	0	-6.313184	0.360064	-0.130654
30	1	0	-4.762542	-2.692759	-0.003844
31	1	0	-7.050711	-1.678274	-0.087068
32	1	0	-7.303622	0.803922	-0.167573
33	6	0	-2.401755	-1.415615	0.004213
34	6	0	-2.694286	1.538849	-0.078908
35	8	0	-2.848704	2.795742	-0.099059
36	8	0	-2.271387	-2.701316	0.050509
37	1	0	-0.917351	-3.163377	0.082711
38	6	0	0.681722	3.417092	1.367173
39	1	0	1.519349	2.821200	1.748949
40	1	0	0.973574	4.474091	1.339994
41	1	0	-0.139611	3.317379	2.086149
42	21	0	4.097329	2.964808	-0.375847

Rotational constants (GHZ): 0.2644306 0.1093432 0.0783334

- Geometry optimization of **KuQ^{2-}-Sc^{3+}** (Sc^{3+} on carbonyl oxygen B) in the *vacuum* (B3LYP functional LANL2DZ basis set) = it converges on carbonyl C

- Geometry optimization of **KuQ^{2-}-Sc^{3+}** (Sc^{3+} on carbonyl oxygen B) in the *vacuum* (B3LYP functional LANL2DZ basis set)

Energy -1231.893069 Hartree
Stoichiometry C23H14O4Sc(1+)
Framework group C1[X(C23H14O4Sc)]
Deg. of freedom 120
Full point group C1 NOp 1
Largest Abelian subgroup C1 NOp 1
Largest concise Abelian subgroup C1 NOp 1
Standard orientation:

Center Number	Atomic Number	Atomic Type	Coordinates (Angstroms) X	Y	Z
1	6	0	0.321895	-0.937450	-0.001562
2	6	0	0.992968	0.339596	-0.056850
3	6	0	-1.089991	-0.668842	-0.006623
4	6	0	2.396357	0.404196	-0.081751
5	6	0	1.058149	-2.139766	0.033740

6	6	0	-1.276802	0.734158	-0.072978
7	6	0	3.186084	-0.802767	-0.046155
8	8	0	2.996527	1.639081	-0.074435
9	6	0	-0.005559	1.383982	-0.102602
10	6	0	2.512580	-2.075936	0.009493
11	8	0	0.487071	-3.353052	0.087307
12	6	0	4.604651	-0.771792	-0.052384
13	6	0	3.278276	-3.266041	0.045878
14	6	0	5.340072	-1.960627	-0.012698
15	1	0	5.114085	0.185868	-0.088200
16	6	0	4.676684	-3.211548	0.033717
17	1	0	2.756273	-4.215986	0.085276
18	1	0	6.425773	-1.926653	-0.018411
19	1	0	5.255752	-4.129634	0.061781
20	6	0	0.202385	2.847218	-0.266913
21	1	0	-0.745960	3.310454	-0.560805
22	1	0	0.880856	3.068087	-1.169529
23	6	0	-3.771426	0.383894	-0.057522
24	6	0	-3.569652	-1.029017	0.004925
25	1	0	-5.218805	1.970667	-0.132545
26	6	0	-5.081053	0.895039	-0.085275
27	6	0	-4.680507	-1.894191	0.038605
28	6	0	-5.985412	-1.370667	0.009495
29	6	0	-6.185366	0.022729	-0.052579
30	1	0	-4.506461	-2.964392	0.086718
31	1	0	-6.839185	-2.041421	0.034900
32	1	0	-7.193978	0.425233	-0.075326
33	6	0	-2.202580	-1.600943	0.034498
34	6	0	-2.617488	1.336864	-0.091110
35	8	0	-2.784817	2.592876	-0.123173
36	8	0	-2.006322	-2.867016	0.086863
37	1	0	-0.551824	-3.315752	0.096875
38	6	0	0.661479	3.636092	1.026146
39	1	0	1.509634	3.195634	1.616125
40	1	0	0.802483	4.724416	0.834934
41	1	0	-0.158620	3.591360	1.747435
42	21	0	2.682659	3.452473	-0.182320

Rotational constants (GHZ): 0.2501545 0.1207262 0.0820728

- Geometry optimization of **KuQ^{2-}-Sc^{3+}** (Sc^{3+} on carbonyl oxygen B) in DCM (B3LYP functional LANL2DZ basis set) = it converges on carbonyl C

- Geometry optimization of **KuQ^{2-}-Sc^{3+}** (Sc^{3+} on carbonyl oxygen C) in DCM (B3LYP functional LANL2DZ basis set)

```
Energy -1232.061675 Hartree
Stoichiometry    C23H14O4Sc(1+)
 Framework group  C1[X(C23H14O4Sc)]
 Deg. of freedom   120
 Full point group                 C1      NOp   1
 Largest Abelian subgroup         C1      NOp   1
 Largest concise Abelian subgroup C1      NOp   1
                        Standard orientation:
 ---------------------------------------------------------------------
 Center     Atomic      Atomic             Coordinates (Angstroms)
```

Number	Number	Type	X	Y	Z
1	6	0	0.156180	-0.873771	0.008243
2	6	0	0.883240	0.380957	-0.062971
3	6	0	-1.260092	-0.564230	0.002737
4	6	0	2.299770	0.345587	-0.081788
5	6	0	0.816924	-2.113352	0.064369
6	6	0	-1.372779	0.871258	-0.064872
7	6	0	3.003422	-0.907476	-0.035210
8	8	0	3.050355	1.498028	-0.141462
9	6	0	-0.087085	1.462336	-0.106575
10	6	0	2.256073	-2.141863	0.045028
11	8	0	0.150811	-3.299618	0.134334
12	6	0	4.430847	-0.990734	-0.065962
13	6	0	2.953971	-3.384608	0.101524
14	6	0	5.086848	-2.215945	-0.010133
15	1	0	5.018154	-0.082029	-0.141462
16	6	0	4.343398	-3.429364	0.077276
17	1	0	2.367843	-4.295002	0.163697
18	1	0	6.173042	-2.250464	-0.034958
19	1	0	4.864461	-4.381464	0.121750
20	6	0	0.183953	2.946719	-0.172219
21	1	0	-0.701614	3.442331	-0.578681
22	1	0	1.010752	3.155312	-0.864571
23	6	0	-3.877960	0.605171	-0.082613
24	6	0	-3.724080	-0.810624	-0.022228
25	1	0	-5.271923	2.240640	-0.179582
26	6	0	-5.172604	1.160733	-0.135488
27	6	0	-4.869505	-1.633171	-0.017190
28	6	0	-6.155280	-1.068030	-0.072754
29	6	0	-6.308723	0.333293	-0.132159
30	1	0	-4.732732	-2.708353	0.028588
31	1	0	-7.031453	-1.710937	-0.070076
32	1	0	-7.302177	0.771065	-0.175471
33	6	0	-2.372220	-1.430851	0.031826
34	6	0	-2.687679	1.521818	-0.087601
35	8	0	-2.852886	2.784454	-0.105475
36	8	0	-2.273897	-2.740880	0.095112
37	1	0	-0.907328	-3.163158	0.127327
38	6	0	0.480678	3.574535	1.218500
39	1	0	1.367389	3.128660	1.689368
40	1	0	0.638939	4.657597	1.126190
41	1	0	-0.367608	3.415747	1.894495
42	21	0	4.106447	2.953041	-0.255482

Rotational constants (GHZ): 0.2638128 0.1094602 0.0781334
